# A pathway level analysis of PFAS exposure and risk of gestational diabetes mellitus

**DOI:** 10.1186/s12940-021-00740-z

**Published:** 2021-05-22

**Authors:** Rahel L. Birru, Hai-Wei Liang, Fouzia Farooq, Megha Bedi, Maisa Feghali, Catherine L. Haggerty, Dara D. Mendez, Janet M. Catov, Carla A. Ng, Jennifer J. Adibi

**Affiliations:** 1 Department of Epidemiology, Graduate School of Public Health, University of Pittsburgh, Pittsburgh, PA USA; 2 Department of Civil and Environmental Engineering, University of Pittsburgh, Pittsburgh, PA USA; 3 Department of Obstetrics, Gynecology, and Reproductive Sciences, University of Pittsburgh School of Medicine, Pittsburgh, PA USA; 4 Environmental and Occupational Health, Graduate School of Public Health, University of Pittsburgh, Pittsburgh, PA USA

**Keywords:** PFAS, Gestational diabetes mellitus, Thyroid hormones, Glucose metabolism

## Abstract

Per- and polyfluoroalkyl substances (PFAS) have been found to be associated with gestational diabetes mellitus (GDM) development, a maternal health disorder in pregnancy with negative effects that can extend beyond pregnancy. Studies that report on this association are difficult to summarize due to weak associations and wide confidence intervals. One way to advance this field is to sharpen the biologic theory on a causal pathway behind this association, and to measure it directly by way of molecular biomarkers. The aim of this review is to summarize the literature that supports a novel pathway between PFAS exposure and GDM development. Epidemiological studies demonstrate a clear association of biomarkers of thyroid hormones and glucose metabolism with GDM development. We report biologic plausibility and epidemiologic evidence that PFAS dysregulation of maternal thyroid hormones and thyrotropin (TSH) may disrupt glucose homeostasis, increasing the risk of GDM. Overall, epidemiological studies demonstrate that PFAS were positively associated with TSH and negatively with triiodothyronine (T3) and thyroxine (T4). PFAS were generally positively associated with glucose and insulin levels in pregnancy. We propose dysregulation of thyroid function and glucose metabolism may be a critical and missing component in the accurate estimation of PFAS on the risk of GDM.

**Supplementary Information:**

The online version contains supplementary material available at 10.1186/s12940-021-00740-z.

## Introduction

Per- and polyfluoroalkyl substances (PFAS) are synthetic organic chemicals used ubiquitously in numerous industries that contaminate food, water, and air, resulting in human exposure. Some PFAS can be bioaccumulative and toxic. Elevated prenatal PFAS levels have been associated with maternal health disorders, including gestational diabetes mellitus (GDM). GDM is an endocrine disorder that develops during pregnancy and can have lasting impacts on maternal health, partly through conversion to type II diabetes (T2D). The mechanism of GDM development is poorly understood and generally not addressed in epidemiologic investigations on PFAS exposures and their impacts. We review the literature for candidate biomarkers of GDM, specifically highlighting those measuring maternal thyroid and maternal glucose homeostasis. We then review the epidemiologic literature for evidence of PFAS as a putative cause of GDM by way of molecular biomarkers in a pathway that connects PFAS toxicity to GDM. We propose that a systematic approach to examining this pathway through validated biomarker measurement can improve the understanding of the pathophysiology of GDM. Additionally, we suspect that this approach will reveal at which points PFAS have their strongest effects.

### Introduction to perfluoroalkyl and polyfluoroalkyl substances (PFAS)

PFAS are a family of synthetic chemicals diverse in size and structure that contain at least one perfluoroalkyl moiety (C_n_F_2n + 1_-). Frequently studied PFAS include perfluorooctane sulfonate (PFOS), perfluorooctanoic acid (PFOA), perfluorononanoic acid (PFNA), perfluorodecanoic acid (PFDA), perfluoroundecanoic acid (PFUnDA), and perfluorohexane sulfonic acid (PFHxS). PFAS have both hydrophobic and oleophobic characteristics that are used in commercial products to increase stain repellency and non-stick qualities, food processing equipment and packaging to reduce oil transfer, firefighting activities as a component of aqueous film forming foams, and industrial and manufacturing practices for purposes such as liquid cooling [1, 2]. Due to their strong carbon-fluorine bonds, they are resistant to degradation [1]. Regulations have been enacted in many countries to eliminate the most common PFAS, mainly PFOS and PFOA; yet they continue to circulate after production stops due to their ability to persist and accumulate in the environment [1]. Furthermore, new short-chain perfluoroalkyl substances and other structurally diverse PFAS developed by industries as replacements to PFOA and PFOS are increasingly being found to be equally toxic [1]. Therefore, understanding the biological effects of this class of chemicals continues to be a priority.

In a National Health and Nutrition Examination Survey (NHANES) study of 7876 US participants ≥12 years old, four of the 12 PFAS measured (PFOS, PFOA, PFNA, and PFHxS) were detected in 95% of serum samples [3]. Although concentrations of some PFAS, like PFOS, generally declined over the period of 1999 to 2008, others remained constant or increased, including PFOA and PFNA [3]. PFAS levels are highly prevalent globally and measured consistently in US and non-US cohorts [4]. The average half-life of PFAS in the human body varies, with long-chain compounds having increased half-lives and ability to bioaccumulate [1, 5]. Serum half-life measurements have mainly been reported for a selection of long-chain PFAS (PFOS, PFOA, and PFHxS), with a range from 3 to 5 years [1, 6]. Xu et al. found that these legacy PFAS had half-lives of approximately 2 to 3 years in a Swedish cohort and accounted for 90% of the total serum PFAS compared to short-chain PFAS (i.e. perfluorobutane sulfonic acid, perfluoroheptanoic acid), which had half-lives of less than 1.5 years and lower bioavailability [7]. All PFAS, however, have relatively long environmental half-lives due to minimal degradation and can remain bioavailable to humans over long periods of time through exposures such as drinking water [1].

### PFAS impacts on maternal health and pregnancy

Acute hormonal and immune changes underway during pregnancy render women and their fetuses particularly vulnerable to PFAS exposure. As women drink more during pregnancy, water may be a significant exposure source. PFAS plasma levels in pregnant women in Shanghai, a particularly contaminated region, were higher in those drinking tap water compared to purified or bottled water, independent of diet [8]. There is epidemiological evidence that PFAS affects maternal health during pregnancy, including contributing to GDM development. Clinically, GDM is diagnosed as type I diabetes (T1D) or T2D presenting in the second or third trimester of pregnancy that is not preexisting [9]. GDM prevalence in 2015 was 8.8% of pregnancies resulting in live births worldwide [10]. GDM increases the risk of low or high birth weight, preterm birth, and preeclampsia [9]. GDM can also have a long-lasting negative impact on maternal health through its conversion into T2D in approximately 50% or more of women [11] and by initiating vascular changes that increase cardiovascular disease risk [9].

**Fig. 1:**
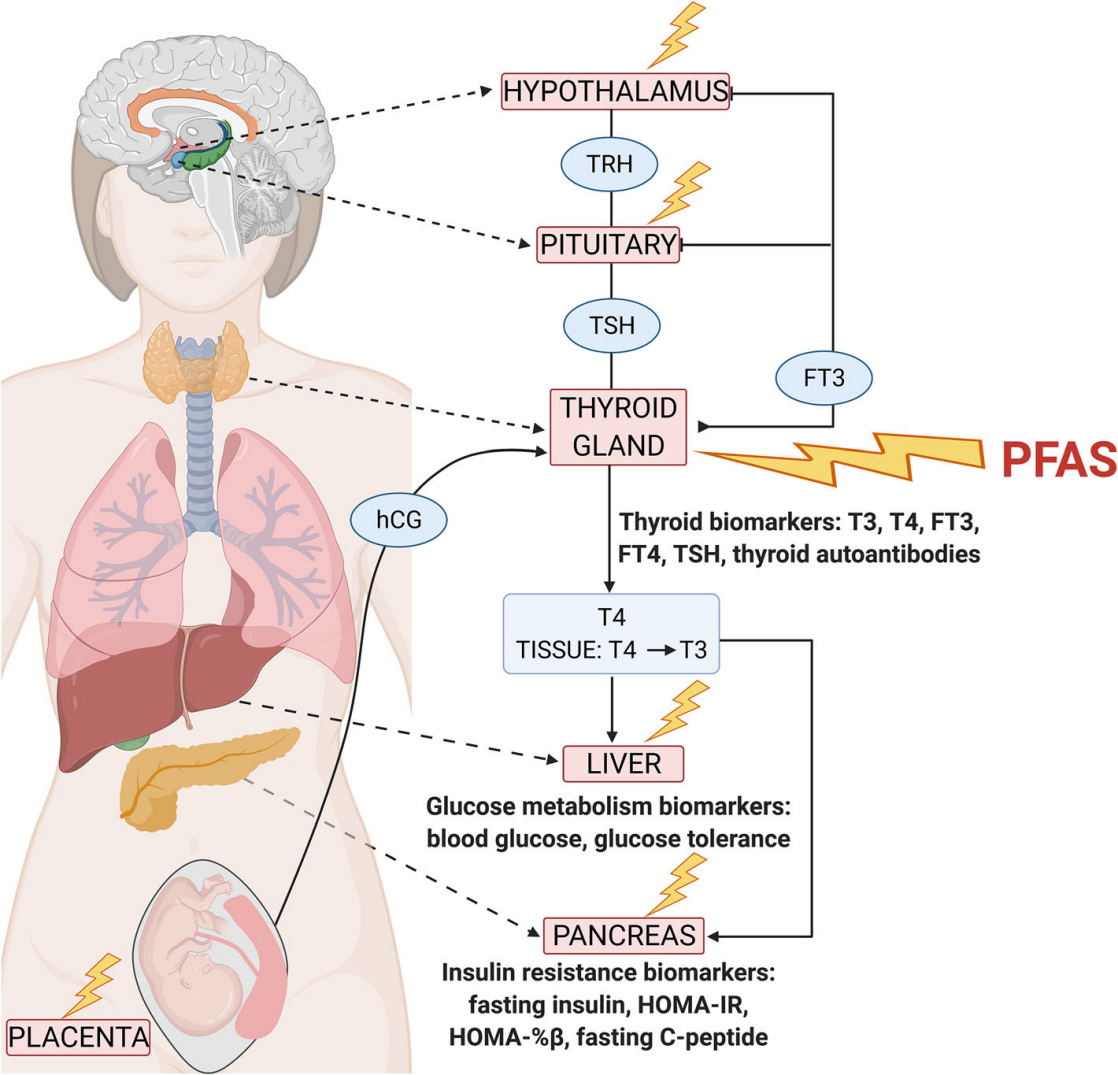
Novel biomarkers in the pathway between PFAS exposure and gestational diabetes mellitus (GDM) development. PFAS targets the hypothalamic–pituitary–thyroid axis, disrupting thyroid hormone homeostasis, which may contribute to GDM development. While the thyroid may be the main target for hormone disruption, PFAS can also exert toxicity to other tissues involved in this regulatory pathway. Abbreviations: FT3, free triiodothyronine; FT4, free thyroxine; hCG, human chorionic gonadotropin; HOMA-%β, homeostatic model assessments of β-cell function; HOMA-IR, homeostatic model of assessment of insulin resistance; T3, triiodothyronine; T4, thyroxine; TRH, thyrotropin-releasing hormone; TSH, thyrotropin

Even though associations of PFAS with diabetes and GDM risk have been reported, there has been minimal work in outlining a detailed and plausible biological mechanism that starts with PFAS and ends with glucose dysfunction that is a proximal cause of GDM [1]. Without a theory on the endocrine mechanism that links this exposure and outcome, associations are difficult to interpret. We narrow the pathway described here to include thyroid hormones (THs) in the maintenance of glucose homeostasis in pregnant and non-pregnant populations. We hypothesize that this is a primary, but not exclusive, pathway relevant to this exposure and outcome. PFAS can disrupt the function of the maternal thyroid, which is a master regulator of glucose homeostasis. Proper thyroid functioning is a known determinant of successful pregnancy outcomes and fetal development, therefore pregnant women are particularly at risk of PFAS thyrotoxicity. This review examines epidemiologic research on circulating biomarkers of thyroid function (hormones, thyroid autoantibodies), glucose metabolism (blood glucose, glucose tolerance), and insulin secretion (insulin resistance) to offer mechanistic insight into subclinical and clinical GDM (Fig. 1). We propose a biologic pathway where PFAS disruption of maternal thyroid function alters circulating TH concentrations and downstream glucose homeostasis, resulting in an increased risk for GDM development.

PFAS encompass thousands of compounds that may elicit different biological responses, particularly new and emerging compounds whose toxicities have yet to be examined. The majority of these compounds, however, have not been studied in relation to the proposed pathway. Therefore, this review is limited to PFAS with the highest circulating concentrations ubiquitously measured in the population and most commonly studied in the literature (Supplemental Table 2), mainly PFOA, PFOS, PFNA, PFDA, PFUnDA, and PFHxS, though there are many PFAS which are not toxic or biologically active in the same way.

### Thyroid hormones regulate normal maternal glucose homeostasis in non-pregnant and pregnant women

The thyroid gland, by way of its hormones, regulates multiple developmental and metabolic pathways involved in energy storage and expenditure, cardiovascular function, and glucose metabolism [12]. TH secretion is controlled by the hypothalamic-pituitary-thyroid axis (Fig. 1) [12, 13]. Briefly, thyrotropin-releasing hormone (TRH) is released by the hypothalamus, which stimulates the release of thyrotropin (TSH) by the pituitary gland [12, 13]. TSH binds to its receptor on the thyroid gland, which results in the production and release of the THs triiodothyronine (T3) and thyroxine (T4) [12]. Circulating THs can be in their biologically active unbound forms free T3 (FT3) or free T4 (FT4) but are primarily bound to transport proteins (over 99%) that facilitate their movement and binding to their receptors across the body [12, 13]. Circulating THs inhibit TRH and TSH release, completing the normal negative feedback loop [12]. Types 1, 2, and 3 iodothyronine deiodinases (D1, D2, and D3, respectively) catalyze the conversion of THs into their biologically active or inactive forms [13]. While circulating TH levels are typically held constant at the ‘global level’ by the hypothalamic-pituitary-thyroid axis, tissue-specific expression and activity of TH receptors, transporters, and deiodinases can modulate TH concentrations to allow for local regulation of TH-controlled processes [14, 15].

Glucose production and storage by the liver is one example of tissue-specific TH action. THs regulate glucose levels by increasing glucose production in the liver. On a transcriptional level, THs regulate several liver enzymes in the gluconeogenesis and glycogenolysis pathways to increase glucose levels, including phosphoenolpyruvate carboxykinase and glucose-6-phosphatase [12, 16, 17]. Additionally, THs regulate glucose transporters. THs increase expression of the hepatic glucose transporter GLUT2, increasing glucose output [17, 18]. T3 increases *GLUT4* gene expression in all cells, which encodes the transporter responsible for the insulin-mediated uptake of glucose [12, 17].

THs also modulate glucose homeostasis by regulating the differentiation and function of the islet cells of the maternal pancreas. Pancreatic islet cells, which produce insulin, express TH receptors [12, 15]. Insulin signals for all cell types in the body to take up glucose and store it as glycogen to maintain physiologically appropriate levels of glucose in the blood. The binding of T3 to the islet transcription factor MAFA results in the maturation of islet cells to become glucose-responsive and insulin-secreting [12, 15].

Disorders in the adult thyroid can disrupt blood glucose control. Hyperthyroidism is defined as low concentrations of TSH and elevated FT3 and/or FT4 concentrations [19]. The thyroid produces excess THs, increasing gluconeogenesis and resulting in elevated blood glucose levels [13, 15] and hepatic insulin resistance [17]. High doses of THs can also impair islet function in the pancreas, resulting in reduced insulin secretion and subsequent glucose intolerance [15]. Hence, hyperthyroidism is a cause of diabetes mellitus both through glucose overproduction and by inhibition of glucose uptake by the cells [13, 15]. Hypothyroidism is clinically defined as elevated TSH and low FT4 concentrations [19] and has also been associated with diabetes mellitus. In this disorder, liver gluconeogenesis is inhibited [17]. The effects of hypothyroidism include altered phosphorylation of insulin signaling proteins, dysregulation of leptin in the hypothalamus, and impaired adipose and skeletal muscle function (such as diminished blood flow and muscle oxidative capacity) [17]. The net result of these changes is increased insulin resistance in the peripheral tissues, resulting in a reduction in glucose utilization and altered glucose homeostasis [17, 18].

Autoimmune thyroid diseases (AITDs) are a main cause of toxicity to the thyroid system and therefore may also indirectly contribute to glucose homeostasis disruption. AITDs are the most common causes of hyperthyroidism and hypothyroidism [20]. These include Graves’ disease (GD), associated with hyperthyroidism, and Hashimoto’s thyroiditis (HT), associated with hypothyroidism. Therefore, markers of AITDs can also be considered as biomarkers for thyroid function. The presence of autoantibodies, namely thyroid peroxidase antibody (TPOAb) and thyroglobulin antibody (TgAb), have been measured in individuals who develop autoimmunity [21]. TPOAb targets the thyroid peroxidase (TPO) enzyme, which is responsible for adding iodine to thyroglobulin (Tg), the precursor of T3 and T4 [13, 22] and TgAb targets Tg [22]. Hence, elevation of either or both TPOAb and TgAb are indicators of potential toxicity to the system. While genetic factors account for approximately 70% of AITDs, environmental toxicant exposures, including PFAS, are also associated with elevated thyroid autoimmunity biomarker levels [23].

### Thyroid and glucose regulation by the fetal placenta during pregnancy

The fetal placenta produces a variety of hormones that enter maternal circulation and stimulate maternal hormone production, including THs [24]. In the first 10 weeks of pregnancy, there is a steady increase in human chorionic gonadotropin (hCG) produced by placental trophoblasts [24]. In the period in which hCG reaches its peak physiological concentrations (3–10 weeks), hCG can bind to the maternal thyroid TSH receptor. Due to its similar structure to TSH, hCG can stimulate TH release, particularly FT4 [24, 25]. The transport protein TBG also rises during this time alongside hCG, stimulated by estrogen, increasing total T4 levels [25]. TSH release by the pituitary gland is suppressed by rising THs and hCG due to the negative feedback relationship [25, 26]. Placental factors other than hCG, such as placental growth factor (PLGF), may also regulate the normal function of maternal thyroid [27, 28].

The fetal thyroid gland does not begins its own hormone production until 16 weeks, yet the fetus requires THs to regulate multiple processes before this point, including successful embryonic implantation and fetal neurodevelopment [29, 30]. The fetus is fully dependent on maternal TH production in the first trimester [31, 32]. Insufficient first trimester maternal TH levels are associated with pregnancy loss and impaired neuropsychomotor development of the child [30, 33, 34]. Transplacental transfer of maternal THs into the fetal compartment is facilitated by TH binding proteins and transporters in the placenta [31, 35]. Deiodinase enzymes in the placenta (D2, D3) regulate fetal exposure to maternal THs, either by metabolizing T4 into the bioactive T3 or inactivating the hormones [35].

In a study of 18,683 pregnant women, women at the 85th–95th percentile of the first trimester hCG multiple of the median (MoM, a measure of hCG that is normalized for gestational age) had 6.4–11.8% lower risk of GDM incidence, respectively [36]. In this study, FT4 mediated 21.4% of the hCG-GDM association [36]. The placenta may be an important mediator in the regulation of glucose homeostasis by the maternal thyroid and subsequent risk of GDM. Higher first trimester hCG was associated with a reduced risk of GDM development and negatively associated with blood glucose levels [36, 37]. As gestational age advances, there is a net effect of changing maternal and placental physiology whereby maternal insulin resistance and blood glucose and free fatty acid (FFA) levels increase [9]. Glucose is transported through the placenta to the fetus to fuel growth [9]. Pancreatic β cell hyperplasia and hypertrophy occur to stimulate more insulin production and to counteract the increasing insulin resistance [9]. After the delivery of the child and the placenta, maternal insulin sensitivity returns to pre-pregnancy levels. The placenta plays an active role in this phenomenon [9] and is under-utilized as a source of biomarkers to study maternal glucose homeostasis.

## Gestational diabetes mellitus (GDM): definition and candidate biomarkers

When glucose homeostasis is highly disrupted in pregnancy due to a dysregulation of the mechanisms described above, women are at risk of developing GDM. GDM is defined as clinical hyperglycemia detected during pregnancy, secondary to insulin resistance [9]. Insulin resistance indicates that the cells are not able to properly utilize glucose leading to glucose levels in the blood that have reached a level high enough to become toxic to the cells. Risk factors such as obesity, advanced age, and family history of diabetes can lead to pancreatic β cell dysfunction, resulting in insufficient insulin production to overcome the insulin resistance associated with pregnancy [9, 38]. This causes blood sugar and FFA levels to increase [9, 38].

### Connecting the dots between thyroid function and gestational diabetes mellitus (GDM)

A causal relationship between THs and the risk of GDM is also supported by epidemiologic findings. Leng et al. reported that higher first trimester TSH levels were weakly associated with increased risk of GDM in a Chinese population of 7258 women (adjusted odds ratio (OR) 1.13, 95%CI 1.00, 1.27) [39]. In a longitudinal study of 321 pregnant women, FT3 and the FT3/FT4 ratio were positively associated with risk of GDM [40]. The adjusted OR of the highest versus lowest quartile of FT3 was 4.25 (95%CI 1.67, 10.80) in the first trimester and 3.89 (95%CI 1.50, 10.10) in the second trimester. For the FT3/FT4 ratio, the adjusted OR was 8.63 (95%CI 2.87, 26.00) in the first trimester and 13.60 (95%CI 3.97, 46.30) in the second trimester. Disruption of T3 and T4 expression as a result of hypothyroidism and hyperthyroidism is also associated with the risk of GDM [41, 42]. Some of these estimates are highly imprecise. Statistical power can be improved by increased sample size and by greater precision in the measurement of the physiologic process, which we aim to promote in this review.

Thyroid autoantibodies have also been studied as causes of GDM. In a meta-analysis, Yang et al. evaluated epidemiological studies published between 1997 and 2014 that assessed the association of thyroid autoantibodies with GDM. Among 21 studies, there was a weak association between thyroid autoantibodies and the risk of GDM (pooled relative risk (RR) 1.12, 95%CI 1.03, 1.22), and it was comparable when women with thyroid dysfunction were included (RR 1.18, 95%CI 1.06, 1.31) [43]. A more recent meta-analysis of thyroid dysfunction and thyroid autoimmunity by Jia et al. of 11 studies assessing thyroid function across the three trimesters of pregnancy showed a strong association between subclinical hypothyroidism, with TPOAb positivity, and GDM (OR 3.22, 95%CI 1.72, 6.03) [44]. Similarly, evidence of an association between TPOAb and GDM was reported in a prospective study of 1683 women by Huang et al., where TPOAb positive women in early pregnancy had an increased risk of GDM (RR 2.54, 95%CI 1.04, 6.23) [45]. These women also had significantly higher TSH and lower FT4 levels compared to euthyroid women. The presence of serum TPOAb and TgAb are indicators of higher GDM risk, making them candidate biomarkers of GDM either alone or in the context of clinically diagnosed thyroid disorders.

### Candidate biomarkers to examine thyroid hormone and glucose disruption in gestational diabetes mellitus (GDM) development

GDM is diagnosed by increased values in blood glucose measurements [38, 46]. Testing strategies in pregnancy vary and include a one-step 2-h 75 g oral glucose tolerance test (OGTT) or a two-step process which starts with a 1-h 50 g oral glucose challenge test (OGCT) followed by a diagnostic 3-h 100 g OGTT in women who screen positive for GDM [47]. These tests are routinely performed starting at 24–28 weeks and can serve as a source of biomarkers for use in epidemiologic studies (Fig. 1). Fasting, post-load (post consumption of a glucose-rich drink used in challenge described above), or specific glucose values can be used to assess a woman’s glucose tolerance during pregnancy.

In research studies, concurrent insulin measurement can be performed to assess insulin resistance and β cell function. A widely validated clinical and epidemiological tool is the homeostasis model assessment (HOMA), which is derived from a mathematical assessment of the balance between hepatic glucose output and insulin secretion based on fasting levels of both glucose and insulin [46, 48]. The Matsuda index is an another method to determine insulin sensitivity from the OGTT [49]. Fasting levels of C-peptide are indicators of pancreatic β cell function. C-peptide is released by these cells during insulin secretion but it degrades slower than insulin, making it a stable biomarker of insulin secretion [50]. Based on the OGTT, a pregnant woman may be diagnosed with isolated hyperglycemia (IH) or impaired glucose tolerance (IGT, a less mild form of glucose intolerance). The researcher can make use of her clinical diagnoses (binomial) or the continuously measured analytes such as fasting glucose or insulin levels to assess associations of THs and subclinical and clinical risk of GDM.

Candidate thyroid biomarkers to examine the causal pathway in maternal circulation include the hormones T3, T4, FT3, FT4, and TSH and thyroid autoantibodies (TPOAb and TgAb) (Fig. 1). These measures are ordered by the obstetrician commonly in the first trimester, but only in women presenting with symptoms of thyroid disease or who are at high risk (those who have an autoimmune disease such as T1D or family history of thyroid disease) [51, 52]. Small changes in FT3 and FT4 can result in a proportionally larger change in TSH due to their negative feedback relationship; therefore, TSH is the more reliable screening analyte for clinical diagnosis [51]. FT4, an accurate marker of thyroid function, is measured concurrently with TSH or in a follow-up screening to confirm a thyroid disorder diagnosis [51, 53]. Thyroid autoantibodies are also measured clinically in a small subset of women to determine women at risk of developing thyroid disorders in pregnancy, particularly those who have an autoimmune disease or have a family history of thyroid disease [51]. Measuring a full panel of THs in the research setting would allow for the most accurate understanding of thyroid function and also allow for assessment for intra- versus inter-individual variability [51, 52].

In the last 10 years, new methods for the measurement of THs have been developed using liquid chromatography-tandem mass spectrometry (LC-MS/MS) that have improved the sensitivity and accuracy of detection [54]. Due to the presence of TH carrier proteins in serum, free TH dissociation and reassociation to binding proteins can occur during sample processing, therefore altering the TH output measurements [54, 55]. This is a particular problem in biospecimens derived from pregnant women, due to the spike in TBG during pregnancy [25, 54]. Equilibrium dialysis or ultrafiltration prior to LC-MS/MS removes TH binding proteins prior to measurement of FT3 and FT4, reducing their interference [54]. LC-MS/MS allows for greater specificity compared to immunoassays by identifying analytes of interest by size rather than by antibodies. Antibodies have varied specificity to analytes and may cross-react with other metabolites in biospecimens, risking false positive results [55]. Sensitivity is enhanced because non-specific binding of antibodies and other types of interference that can mask the antibody-antigen reaction in immunoassays is avoided. This also allows for a reduced lower limit of detection of the analyte of interest [55]. LC-MS/MS methods have recently been developed for thyroid autoantibodies, but so far have comparable accuracy to the immunoassays [53].

Pregnant women are diagnosed with thyroid disease (i.e. hypothyroidism and hyperthyroidism) by comparing TH and antibody levels to pregnancy-specific reference ranges [52, 53]. These trimester-specific ranges account for changes in thyroid biomarker levels during pregnancy, such as the rise in FT4 and drop in TSH during the first trimester, that would otherwise appear as subclinical or clinical thyroid disease when compared to reference ranges for nonpregnant adults [52]. Universal trimester-specific ranges have been difficult to set due to differences in measurement protocols and population differences, such as iodine intake and ethnicity [52]. However, clinical laboratories can create their own reference ranges [52]. Outside of cut-offs and clinically significant TH levels, continuously measured TH biomarkers representing expression across the range of normal to abnormal variation are valuable in epidemiologic investigations to understand exposure effects and to link exposures to outcomes.

## PFAS exposure and risk of gestational diabetes mellitus (GDM) development and candidate biomarkers: evidence in the literature

**Fig. 2:**
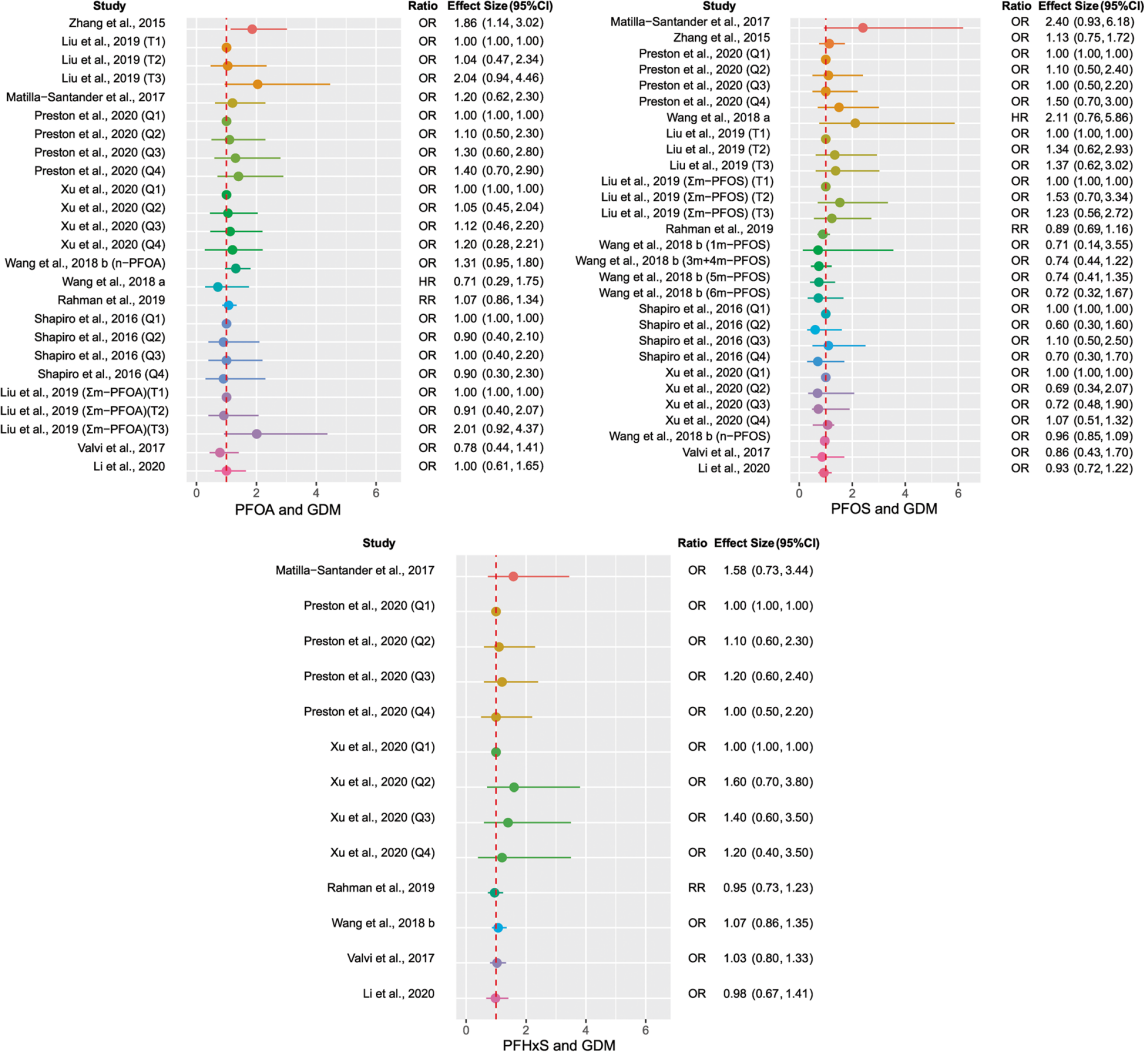
Summary statistics of selected PFAS on gestational diabetes mellitus (GDM) development in the epidemiologic literature. Abbreviations: HR, hazard ratio; OR, odds ratio; PFOA, perfluorooctanoic acid; PFOS, perfluorooctane sulfonate; PFHxS, perfluorohexane sulfonic acid; RR, risk ratio

**Fig. 3:**
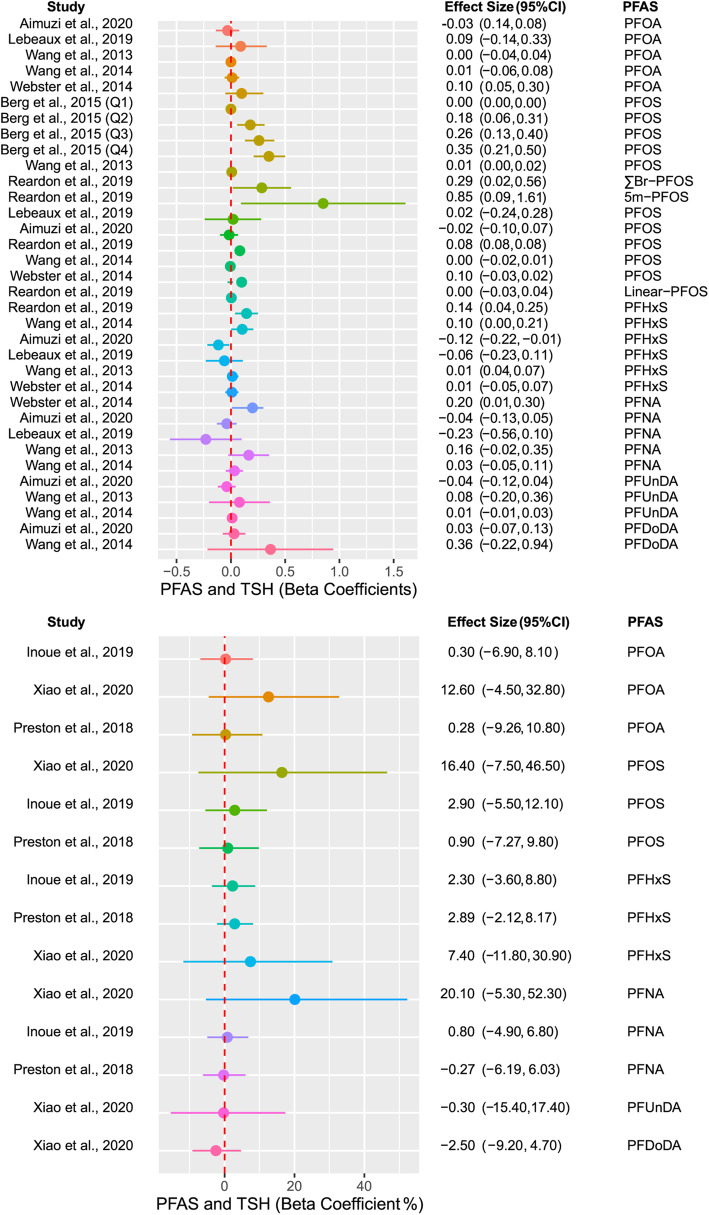
Summary statistics of selected PFAS on thyrotropin (TSH) in the epidemiologic literature. Displayed is a representative forest plot of the PFAS and TSH relationship for selected PFAS where not all units are the same across all studies. The aim is to assess the overall direction and strength of association. Abbreviations: PFOA, perfluorooctanoic acid; PFOS, perfluorooctane sulfonate; PFHxS, perfluorohexane sulfonic acid; PFNA, perfluorononanoic acid; PFUnDA, perfluoroundecanoate; PFDoDA, perfluorododecanoate; TSH, thyrotropin

Epidemiologic literature was reviewed and summarized according to specific criteria (Supplement: Methods). Data were extracted and compiled in the Supplemental Tables 1–8 and summarized in Table 1. Forest plots were prepared for representative PFAS that displayed the most significant associations with selected outcomes (Figs. 2, 3). Null coefficients are not interpretable but collectively may offer insight on relationships that were underpowered but worthy of considering in future studies. Therefore, we created criteria to assess the direction of association for all outcomes moving beyond a *p* value of less than 0.05, as described in the Methods (Supplement).

**Table 1:** Summary of the epidemiologic studies reviewed, according to the direction of association of PFAS exposure on outcomes in the proposed pathway

Outcome	Positive	Negative	Null, Positive Trend^b^	Null, Negative Trend^b^
**GDM**	36% (4/11 studies)	0% (0/11 studies)	91% (10/11 studies)	64% (7/11 studies)
**Maternal Blood**				
**Glucose homeostasis biomarkers**^a^	73% (8/11 studies)	18% (2/11 studies)	45% (5/11 studies)	45% (5/11 studies)
**T3**	50% (2/4 studies)	50% (2/4 studies)	25% (1/4 studies)	25% (1/4 studies)
**FT3**	33% (2/6 studies)	33% (2/6 studies)	17% (1/6 studies)	17% (1/6 studies)
**T4**	20% (1/5 studies)	40% (2/5 studies)	20% (1/5 studies)	20% (1/5 studies)
**FT4**	20% (2/10 studies)	40% (4/10 studies)	20% (2/10 studies)	30% (3/10 studies)
**TSH**	45% (5/11 studies)	18% (2/11 studies)	18% (2/11 studies)	9% (1/11 studies)

*Abbreviations*: *GDM* gestational diabetes mellitus, *FT3* free triiodothyronine, *FT4* free thyroxine, *T3* triiodothyronine, *T4* thyroxine, *TSH* thyrotropin

^a^Glucose homeostasis biomarkers: isolated hyperglycemia, impaired glucose tolerance, blood glucose and insulin levels, insulin resistance

^b^Criteria defined in detail in the Methods (Supplement)

### PFAS and GDM

Out of the 11 epidemiological studies that reported on the association of PFAS and GDM [56–66], four (44%) demonstrated a positive association, differing by PFAS compound (Table 1, Supplemental Table 3) [56, 59, 62, 63]. Overall, higher PFAS levels were associated with higher risk of GDM development. Effect sizes were small and confidence intervals were wide, indicating lack of precision and/or potential bias. The strongest associations were found with PFOA, PFOS, and PFHxS, which are presented in a forest plot comparison (Fig. 2).

### PFAS exposure and biomarkers of glucose homeostasis

We next examined the association of PFAS with biomarkers of glucose homeostasis that could putatively mediate the risk of GDM. These biomarkers do not indicate frank disease but are meaningful in understanding mechanism and chronic disease risk. A two-fold increase in serum PFHxS in Danish pregnant women was positively associated with fasting glucose and insulin levels (percentage change 1.7% (95%CI 0.2, 3.2) and 7.7% (95%CI 0.1, 15.9), respectively) [67]. Additionally, higher PFNA was associated with increased fasting insulin and β cell function (percentage change 12.1% (95%CI 0.7, 24.8) and 12.4% (95%CI 2.2, 23.7), respectively) [67]. Eleven epidemiological studies reported on the association of PFAS with glucose and insulin biomarkers in pregnant women [56–58, 60, 61, 64, 65, 67–70], with eight (73%) finding a positive association, depending on the PFAS [56–58, 60, 64, 65, 67, 69] (Table 1, Supplemental Table 4). Only two studies (18%) reported negative associations [68, 70], though levels of PFAS were lower in their study cohort compared to others examining this association (Supplemental Table 2). Additionally, the Mehta et al. study only included overweight and obese pregnant women enrolled in a stress-reduction intervention as a means to control weight gain and therefore this cohort may not reflect the general population [70]. There was evidence of nonmonotonic associations in the case of PFAS and glucose biomarkers, though these were ultimately null associations after adjustment for confounders (Supplemental Table 4). The general positive association of PFAS with glucose homeostasis biomarkers in these studies is in agreement with epidemiological studies examining PFAS exposure in non-pregnant individuals with both normal and elevated baseline glucose levels. In these populations, PFAS was found to be associated with an increased risk of diabetes and disruption of glycemic indicators, including increased blood glucose, glycated hemoglobin (HbA1c), and insulin secretion [71, 72].

### PFAS exposure and biomarkers of thyroid function

An NHANES study of three independent study periods between 1999 and 2006 reported that among US non-pregnant adults (*n* = 3974), participants with thyroid disease had greater serum PFAS levels than those without [73]. A recent meta-analysis of 12 studies examining the effects of PFAS exposure on TH levels in adults found that PFAS are positively associated with FT4 and TSH but negatively associated with total T3 and T4 [74]. In a subgroup analysis of four studies with pregnant women within this meta-analysis (*N* = 1735), PFAS were not associated with THs [74]. Information on gestational age at the time of blood sampling for PFAS and TH measurements was not collected or controlled for; yet gestational age is a source of variability in both measures. This could be one reason for a null association.

There were 13 individual studies reviewed that measured the associations of PFAS and THs in maternal circulation [75–87], with ten (77%) finding a positive or negative association [75, 76, 79–85, 87] (Table 1, Supplemental Tables 5–7). Berg and colleagues published two studies examining associations of PFAS with maternal TH levels using the same cohort. Results were extracted from their 2015 study only [76, 88]. Generally, PFAS were negatively associated with THs and positively associated with TSH (Table 1). This is consistent with the idea that PFAS disrupts TH homeostasis, resulting in a reduction in TH levels that can stimulate TSH secretion due to the negative feedback relationship. In a systematic review examining the relationship between PFAS and THs, Ballesteros et al. also concluded that PFHxS and PFOS are generally found to be positively associated with maternal TSH in pregnancy [89]. Of the studies included in our review, the strongest PFAS and thyroid biomarker relationship was with TSH (Fig. 3).

Of the six studies that performed a subanalysis stratifying women by thyroid antibody status, all six reported that maternal thyroid autoimmunity altered PFAS disruption of the thyroid based on effect measure modification (Supplemental Table 8) [75, 79–82, 85]. In these studies, it is not presumed that PFAS increased autoimmunity but that PFAS may exacerbate the autoimmune damage to further dysregulate TH production [79, 82, 85]. Itoh et al. found that maternal PFAS levels were associated with FT3 in both maternal thyroid autoantibody positive and negative groups [79]. Consistent with studies that did not measure thyroid autoantibody status, Webster et al. found that PFAS increased TSH and lowered FT4 levels in TPOAb positive pregnant women. Effects were milder in pregnant women with normal TPOAb levels [85]. However, in a recent US study, TPOAb positive women had a negative association between PFAS exposure and TSH levels [81], inconsistent with other studies reporting a positive association [75, 76, 82, 83, 85]. The authors noted that this discrepancy could be a result of study design differences, particularly iodide status of their study population, which affects TH synthesis [81, 85]. Overall, the results indicate that thyroid autoimmunity is a key variable to consider.

Beyond disrupting maternal TH homeostasis, PFAS can be transferred from maternal circulation to the fetus to alter fetal TH levels. Several studies included in this review examined the impact of PFAS on fetal THs by measuring the association of PFAS levels with cord blood and neonatal heel stick TH levels [80, 81, 83, 86, 87]. While results varied, maternal PFAS levels were generally positively associated with fetal and neonatal TSH and negatively with T3 and T4. THs regulate metabolic and physiological pathways that determine pregnancy outcomes (i.e. birth size) and development (i.e. neurodevelopment) [35]. Therefore, in addition to affecting maternal health, PFAS thyrotoxicity in pregnancy may alter fetal growth and development, with long-lasting impacts on child health.

## Future directions: epidemiological modeling of PFAS and gestational diabetes mellitus (GDM)

To accurately estimate the association of PFAS and GDM risk, study design plays an important role. Longitudinal, unlike cross-sectional, studies allow for the identification of temporality in the relationship. For example, Reardon et al. found that PFHxS was positively associated with TSH and negatively with FT4 [82]. The association was time-dependent for TSH, with the effect size decreasing as gestation progressed [82]. Measuring biomarkers at standardized points throughout gestation can yield less biased conclusions on the relationships proposed here and improve generalizability and transportability to other populations.

This pathway approach primarily strengthens the potential for causal inference by way of a more accurate and precise measurement of the phenomenon of interest. It reduces bias due to measurement error by capturing information on physiologically relevant processes and by including multiple measures within an individual. If biomarkers are measured longitudinally in pregnancy, that likewise strengthens the potential for measuring a causal effect.

The subdiscipline in epidemiology called ‘causal inference’ lays out specific techniques for estimation, inference, and quantitative analysis of bias [90, 91]. The basis of this approach is the potential outcomes framework. This is a hypothetical framework by which all people who are unexposed in a study and have a set of outcomes can essentially be taken back in time, and then be given the exposure in order to measure their set of outcomes under that condition. The probability of outcome under the two scenarios can be compared to get a causal effect estimate. This is not feasible in real life. The objective is to approximate this scenario by applying analytical techniques. Most pregnant women are exposed to PFAS so the idea of a no exposure group is also unrealistic. For this reason, we might instead estimate the difference in risk if all pregnant women had PFAS levels at the 75th percentile versus all women who had levels at the 25th percentile.

A first step in this approach is to generate a directed acyclic graph (DAG) that can structure the relationship of the exposure and the outcome in relationship to confounders, intermediate variables, and colliders [92]. This is a way to identify sources of structural bias that can be minimized by collecting information, adjustment, or inverse probability weighting. Confounders are those variables which are common causes of the X (exposure), the M (intermediate), and Y (outcome) variables in three categories: 1) confounders of PFAS (X) and GDM (Y); 2) confounders of PFAS (X) and intermediate biomarkers (M, maternal thyroid and pancreas, fetal placenta); and 3) confounders of intermediate biomarkers (M) and GDM (Y). Effect modifiers are variables such as thyroid autoimmune biomarkers which might be operating in tandem with PFAS exposure in some fashion but may not necessarily be intermediates in a causal pathway. Effect modifiers can be identified and evaluated based on knowledge of mechanism and previously reported findings, but they cannot be represented in a DAG [93]. Analytical strategies for constructing DAGs are described elsewhere [92, 94, 95]. Statistical approaches to analyze these associations are outside of the scope of this review, but also an important area in developing this pathway-level approach. G methods have been proposed for the analysis of complex, longitudinal data with time-varying confounders [96]. Methods that allow for single or multiple mediators may also be useful in estimation of PFAS effects in the presence of measures of placental, thyroid, liver, and/or pancreatic function [97].

Adjustment for variables that are not confounders can also lead to bias in the estimation of the exposure and outcome association [98]. Variables that were adjusted for but may not be confounders in the studies reviewed are noted in Supplemental Table 1. To qualify as a confounder, the variable must precede the exposure in time and plausibly be a common cause of the exposure and the outcome.

## Discussion

The evidence of PFAS exposure as a direct cause of GDM in the epidemiological literature is weak. This conclusion could change in future studies that consider the indirect pathway and measure the biomarkers proposed here. Based on the results of this review, we theorize that PFAS can alter TH homeostasis in pregnancy by causing higher TSH levels and lower T3 and T4 levels, disrupting downstream glucose metabolism. TH disruption may be happening at the level of the thyroid gland, the hypothalamus, the pituitary, or the fetal placenta (Fig. 1).

There is experimental evidence of the toxicity of PFAS to the thyroid. PFOS and PFHxS were found to inhibit iodide uptake in the FRTL-5 rat thyroid follicular cell line [99, 100]. These effects were not seen with PFOA, indicating compound specific effects on the thyroid [99]. In zebrafish, PFUnDA and PFOA induced changes in the expression of genes involved in TH metabolism and excretion [101, 102] while perfluorotridecanoic acid (PFTrDA) upregulated genes related to TH activation and synthesis [101]. Furthermore, PFAS may competitively bind to TH binding proteins to displace T4 [103] and to increase transcript expression of D1, an enzyme that converts T4 to T3, in the thyroids of rats [104]. All together, PFAS may therefore be altering TH homeostasis by direct interaction with the thyroid gland and through mechanisms that control TH availability. Based on the results of our literature review, PFAS may also exacerbate underlying thyroid autoimmunity. Approximately 30% of pregnant women during early pregnancy were found to be TBOAb/TgAb positive [105], confirming that this is a variable that cannot be ignored.

From our findings, we infer that PFAS exposure also causes glucose and insulin levels to increase in pregnancy. We propose that changes in glucose and insulin levels here may be a secondary consequence of thyroid dysregulation. This may also reflect direct toxicity of PFAS to the maternal liver and/or pancreas that could disrupt maternal and fetal glucose homeostasis during pregnancy. Molecular docking analysis found that PFOS, PFOA, and PFHxS can directly bind multiple peroxisome proliferator-activated receptor (PPAR) isoforms in the liver that regulate glucose metabolism [106]. Glycogen depletion and mitochondrial dysfunction are PPAR-independent mechanisms by which PFAS exerts toxicity in the liver [107]. In carp, a closely related species to zebrafish, PFOS exposure inhibited liver glucokinase gene expression, which regulates glucose uptake into the liver, and depleted glycogen stores in the liver, therefore altering glucose homeostasis [108]. Glycogen depletion was also observed in the livers of pregnant mice exposed to PFAS, along with hepatocellular alterations, such as hypertrophy and necrosis [109]. Additionally, Sant et al. determined that zebrafish embryonic exposure to PFAS induced morphological changes to the pancreas of zebrafish larvae, including reduced β cell area for insulin production, as well as reduced gene expression of hormones related to glucoregulation [110].

The majority of PFAS examined demonstrated null associations with GDM and the biomarkers proposed in this causal pathway. This may be a result of limitations in study design when examining PFAS cross-sectionally or sampling cases and controls versus a prospective cohort design. Findings could also be null due to measurement error or incorrect model specification. Incorrect model specification here includes the omission of interactions or intermediate variables. These are all issues that the proposed pathway approach can address. Another issue is that due to this being a large and diverse class of chemicals, the toxicological profiles of different PFAS vary dramatically. Therefore, there may be only particular compounds that impact the specific biomarkers proposed in this pathway. Biomarker-based studies are needed to narrow these lists and identify these specific relationships. Additionally, human exposure to PFAS is typically through complex mixtures and multiple routes of exposure, rather than to individual compounds [1]. In mixtures, the compounds have been shown to exert both additive and synergistic toxicities; therefore, examining the overall mixture effect may be more accurate in depicting the human exposure [111]. Few studies included in our review examined the combined effects of the PFAS on the outcomes. Chan et al. examined the association of the molar sum of PFHxS, PFOA, and PFOS with hypothyroxinemia in pregnant women but found no additive or synergistic effect, as compared to analyzing each compound alone [77]. Three studies [58, 70, 80] used Bayesian Kernel Machine Regression (BKMR) as a statistical approach to determine the PFAS mixture effect while also providing associations for each compound in the mixture to understand which are the main drivers of the overall association [112]. Correlations between mixture components are also taken into account [112]. With this approach, a PFAS mixture (six compounds) was associated with plasma glucose levels [58]. The single compounds PFOS and 2-(N-methyl-perfluorooctane sulfonamide) acetate (N-MeFOSAA) were the main contributors to this overall association [58]. Mixture modeling can be useful in identifying the ‘bad actors.’ There is no perfect model to account for these complex relationships, which is one reason it is impossible to conclude that PFAS associations with GDM are truly null.

Impairments in placental function can affect maternal health during pregnancy (i.e. preeclampsia). It follows that PFAS-induced effects on the placenta may also adversely affect the mother, as well as the fetus. The placenta is exposed to PFAS from maternal circulation [113]. In animal models, PFAS exposure causes both increases and decreases in placental weight, inhibits activity and gene expression of placental hormones, and induces necrosis and histopathological alterations, with compound specific effects [109, 114, 115]. Rodents do not produce hCG, so this model does not translate directly. Placental biomarkers altered by PFAS exposure during pregnancy, including but not exclusive to hCG, should be prioritized when studying this causal pathway.

Reverse causation, whereby thyroid dysfunction precedes and causes physiological differences that affect maternal PFAS concentrations, cannot be eliminated. For example, THs regulate multiple processes in the kidney, including the glomerular filtration rate (GFR), which is the rate of fluid filtered by the kidneys. Renal clearance is the primary mode of excretion of PFAS and a lower GFR is associated with higher serum PFAS levels [116]. Any alteration in thyroid function could in theory change the GFR and rate of excretion of PFAS, therefore affecting serum levels of PFAS. Watkins et al. measured the direction of association between PFOA and kidney function by comparing measured versus model-predicted (based on environmental exposure estimates) serum PFOA levels, which are dependently and independently influenced by GFR, respectively [116]. Only the measured PFOA was associated with kidney dysfunction, which may indicate that PFAS is a result of rather than the cause of the kidney function. Other physiological processes have been proposed that may demonstrate reverse causality in the causal association of PFAS with health outcomes [117]. Adding confounders related to circulating PFAS levels, such as GFR, into the model of PFAS exposure and GDM development could address this issue of reverse causation, but it cannot be completely eliminated when measures are cross-sectional in pregnancy.

The pathway proposed here may be influenced by a broad range of environmental exposures beyond PFAS. For example, organochlorine pesticides (OCPs) used for crop protection, such as dichlorodiphenyl trichloroethane and hexachlorobenzene, are also potential candidates for disruption of thyroid function [118, 119]. Several OCPs were associated with TSH and FT4 levels in cord plasma [120] and with the risk of developing GDM [121]. Likewise, exposure to brominated flame retardants, such as polybrominated diphenyl ethers (PBDEs), have been positively associated with GDM risk [122]. PBDEs are negatively associated with total T3 and T4 in cord serum and with altered birth outcomes, including lower IQ [123–125]. Furthermore, endocrine disruptors in plastics and personal care items, including phthalates and bisphenol A, have been shown to affect TH regulation including impairment of iodine uptake and inhibition of TH homeostasis [126] and phthalates have been found to be associated with hCG, glucose intolerance, and GDM [127–129].

GDM is a common pregnancy complication that may progress to adverse maternal and child health outcomes and can be better appreciated as a consequence of environmental exposure preconception and during pregnancy. Here, we present the evidence to date on PFAS as a relevant exposure. On the clinical side, this knowledge can be used to distinguish patients at different levels of risk for GDM and conversion of GDM to T2D, and also to formulate stronger individual-level interventions related to PFAS exposures through diet and drinking water in pregnancy. On the level of public health, these types of studies can be useful in generating causal knowledge on population-level exposures that increase the risk of GDM to motivate interventions when supported by the evidence. If consensus is reached regarding specific and harmful effects of PFAS, these types of studies could motivate innovation in the design of chemicals used in food packaging and commercial products. The biomarkers proposed in this pathway can be monitored in large cohorts to evaluate efficacy of PFAS regulation and/or more targeted types of interventions.

## Conclusions

Epidemiological evidence strongly supports that problems with thyroid function can disrupt glucose homeostasis, which we posit may be a driving force for the development of GDM and may be a mechanism through which PFAS exposures exert toxicity in pregnant women. This mechanism may offer an opportunity to more precisely and accurately quantify the associations of PFAS (and other thyroid-disrupting chemicals) with GDM. The relationship between TH biomarkers and GDM as well as glucose biomarkers and GDM are well established, however the combination of these two associations into one common pathway is relatively unexplored. PFAS exposure may be an upstream environmental lever that interferes with thyroid function and disrupts downstream glucose homeostasis.

## Supplementary Information

**Additional file 1: Methods. Supplemental Table 1.** Summary of studies examining PFAS and gestational diabetes (GDM), glucose, or thyroid biomarkers in pregnancy. **Supplemental Table 2.** Summary of PFAS and biomarker levels in cohorts. **Supplemental Table 3.** Association of PFAS with gestational diabetes mellitus (GDM). **Supplemental Table 4.** Association of PFAS with glucose and insulin biomarkers. **Supplemental Table 5**: Evidence of PFAS association with free and total triiodothyronine (T3) in pregnancy. **Supplemental Table 6:** Evidence of PFAS association with free and total thyroxine (T4) in pregnancy. **Supplemental Table 7:** Evidence of PFAS association with thyrotropin (TSH) in pregnancy. **Supplemental Table 8:** Evidence of PFAS association with thyroid hormones by thyroid autoantibody status in pregnancy.

## Data Availability

All data generated or analyzed during this study are included in this published article [and its supplementary information files].

## Abbreviations

AITDs: Autoimmune thyroid diseases
D1: Type 1 iodothyronine deiodinases
D2: Type 2 iodothyronine deiodinases
D3: Type 3 iodothyronine deiodinases
FFA: Free fatty acid
FT3: Free triiodothyronine
FT4: Free thyroxine
GDM: Gestational diabetes mellitus
hCG: Human chorionic gonadotropin
HOMA: Homeostasis model assessment
PLGF: Placental growth factor
PFAS: Per- and polyfluoroalkyl substances
T1D: Type I diabetes
T2D: Type II diabetes
T3: Triiodothyronine
T4: Thyroxine
TBG: Thyroxine-binding globulin
Tg: Thyroglobulin
TgAb: Thyroglobulin antibody
TH: Thyroid hormone
TPO: Thyroidperoxidase
TPOAb: Thyroid peroxidase antibody
TRH: Thyrotropin-releasing hormone
TSH: Thyrotropin
