# Supplementary material for: A pathway level analysis of PFAS exposure and risk of gestational diabetes mellitus
Source: Environ Health. 2021 May 22;20:63. doi: 10.1186/s12940-021-00740-z (PMC8141246; doi:10.1186/s12940-021-00740-z)
Supplement: Supplementary file 1 — Additional file 1: Methods. Supplemental Table 1. Summary of studies examining PFAS and gestational diabetes (GDM), glucose, or thyroid biomarkers in pregnancy. Supplemental Table 2. Summary of PFAS and biomarker levels in cohorts. Supplemental Table 3. Association of PFAS with gestational diabetes mellitus (GDM). Supplemental Table 4. Association of PFAS with glucose and insulin biomarkers. Supplemental Table 5: Evidence of PFAS association with free and total triiodothyronine (T3) in pregnancy. Supplemental Table 6: Evidence of PFAS association with free and total thyroxine (T4) in pregnancy. Supplemental Table 7: Evidence of PFAS association with thyrotropin (TSH) in pregnancy. Supplemental Table 8: Evidence of PFAS association with thyroid hormones by thyroid autoantibody status in pregnancy. [file 12940_2021_740_MOESM1_ESM.docx]

**A pathway level analysis of PFAS exposure and risk of gestational diabetes mellitus.**

Rahel L. Birru, Hai-Wei Liang, Fouzia Farooq, Megha Bedi, Maisa Feghali, Catherine L. Haggerty, Dara D. Mendez, Janet M. Catov, Carla A. Ng, Jennifer J. Adibi

**Table of Contents:**

1. Methods (pgs. 2-5)
2. Table 1: Summary of studies examining PFAS and gestational diabetes (GDM), glucose, or thyroid biomarkers in pregnancy (pgs. 6-10)
3. Table 2: Summary of PFAS and biomarker levels in cohorts (pgs. 11-21)
4. Table 3: Association of PFAS with gestational diabetes mellitus (GDM) (pgs. 22-29)
5. Table 4: Evidence of PFAS association with glucose and insulin biomarkers in pregnancy (pgs. 30-49)
6. Table 5: Evidence of PFAS association with free and total triiodothyronine (T3) in pregnancy (pgs. 50-55)
7. Table 6: Evidence of PFAS association with free and total thyroxine (T4) in pregnancy (pgs. 56-64)
8. Table 7: Evidence of PFAS association with thyrotropin (TSH) in pregnancy (pgs. 65-70)
9. Table 8: Evidence of PFAS association with free/total thyroid hormones by thyroid autoantibody status in pregnancy (pgs. 71-81)
10. Per- and polyfluoroalkyl substances (PFAS) compound abbreviations (pg. 82-83)
11. References (pgs. 84-86)

**Methods**

PubMed (1950-April 2021) was used to retrieve epidemiological studies examining the causal pathway of the association of per- and polyfluoroalkyl substances (PFAS) with gestational diabetes mellitus (GDM). Google Scholar was used to retrieve additional articles. Studies were included if they met the following criteria: 1) epidemiological study in pregnant women, 2) analyzed association of maternal circulating PFAS with GDM, maternal glucose biomarkers, and/or maternal thyroid hormones (THs) in pregnancy, 3) full text published in English. Exclusion criteria included reviews, commentaries, and animal studies. Textual, medical subject heading (MESH), or title/abstract (TIAB) search terms for PFAS, GDM, and thyroid biomarkers were used. The search and data extraction were completed by four authors independently (FF, HL, MB, RB).

| **Search Terms** | | |  |
| --- | --- | --- | --- |
| **PFAS** | **GDM** | **Thyroid Biomarkers** | **Pregnancy** |
| 1. per- and polyfluoroalkyl substances 2. perfluoroalkyl and polyfluoroalkyl substances perfluoroalkyl 3. polyfluoroalkyl 4. PFAS 5. perfluorooctanoic acid 6. perfluorooctane sulfonic acid 7. perfluoropentacoic acid 8. perfluorohexanoic acid 9. perfluoroheptanoic acid 10. perfluorononanoic acid 11. perfluorodecanoic acid 12. perfluoroundecanoate 13. perfluoroheptanoate 14. perfluorobutansulfonic acid 15. perfluorohexanesulfonic acid 16. nadon 17. genx 18. hpfo-da 19. 9cl-pf3ons 20. 11cl-pf3ouds 21. PFOA 22. PFOS 23. PFPeA 24. PFHeA 25. PFHxA 26. PFHpA 27. PFNA 28. PFDA 29. PFUnDA 30. PFHpA 31. PFBS 32. PFHxS | 1. gestational diabetes 2. gestational diabetes mellitus 3. GDM 4. glucose 5. insulin | 1. thyroid 2. thyroid hormone 3. triiodothyronine 4. thyroxine 5. thyroid stimulating hormone 6. thyrotropin 7. thyrotropin-releasing hormone 8. T3 9. T4 10. TSH 11. TRH 12. thyroid antibodies 13. thyroid autoantibodies 14. thyroglobulin 15. thyroid peroxidase 16. Tg 17. TgAb 18. TPO 19. TPOAb | 1. pregnancy 2. pregnant 3. maternal |

The criteria for assessing the direction of the association for the studies in Tables 2 and 3 were developed to consider statistical significance and also to go beyond the traditional and singular emphasis on P-value cut-offs of 0.05 to summarize relevant evidence. The P-value can be a useful starting point to separate studies with stronger vs. weaker statistical evidence of an association. P-values are also largely a function of sample size and modeling and adjustment strategy, and therefore may not be useful in distinguishing truly informative vs. non-informative studies [1-4]. Given the complexity of our question, we took a more comprehensive approach to evaluating associations that considered sample size, the effect size, direction of effect, and precision in the effect estimate. Outside of these criteria, we also evaluated in **Supplemental Table 1** the choice of variables to include in the model to point out situations where the associations may be subject to overadjustment or collider bias due to misclassified confounders. Our criteria are as follows:

1. Correlation coefficients: For studies that assess the strength of association based on correlation coefficients (r, ρ), we classified a correlation of <0.40 as weak (positive (+) or negative (-) based on the direction of the correlation), 0.4-0.70 as moderate, and >0.70 as strong.
2. Odds Ratio (OR): Studies that assessed the strength of association based on ORs were reported as follows:
   1. If the study reported an OR with a 95% confidence interval (95%CI) that did not include 1.0, the study’s finding was classified as statistically significant and was further classified as positive or negative based on whether the OR was above or below 1.0. Some studies assigned the reference level for an OR as 0.0, instead of the default 1.0; in these cases, we assessed whether or not the 95%CI included 0.0, and whether the OR was above or below 0.0.
   2. If the 95%CI did include 1.0 (or 0.0 for studies that used 0.0 for the reference category), but the OR was above 1.1 or below 0.9, we listed the association as positive or negative depending on the direction of the association. Studies that did not satisfy this criterion were reported as having null findings.
   3. If studies reported ORs as tertiles/quartiles and an overall monotonic trend was observed, we classified the findings as positive or negative, based on whether one or more tertile/quartile ORs were above 1.1 or below 0.9, regardless of statistical significance based on either a 95%CI or p-value. If there was no overall trend and only one of the levels was statistically significant, we assign it a positive or negative, depending on the direction. If the trends are nonmonotonic and there is no statistical significance, the results were classified as nonmonotonic.
3. β coefficients: For studies that reported β coefficients and reported a 95%CI that did not include 0.0 or reported a p<0.05, we reported the finding as a positive or negative association. Studies that reported findings that were not statistically significant were labeled as indeterminate. For these regression-based models, there is no uniform scale on which to evaluate the magnitude of an effect, unlike odds ratios. For this reason, we placed more emphasis on the precision of the beta coefficient.
4. Percent (%) change: To assess the strength of association for studies that reported the % change defined, for example, as (exp(β) - 1) × 100, we made the initial assessment based on p<0.05 (or whatever alpha was established) or based on the 95%CI. If the change was statistically significant, we listed the finding as a positive or negative change depending on the direction of the association. If the % change was not statistically significant, we assessed whether the absolute value of change was greater than 10% or less than -10%. Any reported change of a magnitude less than 10% for non-significant levels was considered a null association.

**SUPPLEMENTAL TABLE 1. Summary of studies examining PFAS and gestational diabetes (GDM), glucose, or thyroid biomarkers in pregnancy**

|  | **Author** | **Study Design** | **Country** | **Cohort Age** | **n** | **Relationship Examined** | **Confounding Factors Included** |
| --- | --- | --- | --- | --- | --- | --- | --- |
| 1 | Aimuzi et al., 2020 [5] | Prospective | China | mean±SD: 29±3.43 | 1885 | PFAS and THs, TAb subanalysis | maternal age, pre-pregnancy BMI, gestational age at TH measurement, fish intake, maternal education, difference between PFAS and THs measured gestational weeks, specific hospital |
| 2 | Berg et al., 2015 [6] | Longitudinal | Norway | median: 32 | 391 | PFAS and THs | age, BMI, parity, thyroxin binding capacity |
| 3 | Chan et al., 2011 [7] | Cross-sectional | Canada | mean±SD: 31.3±4.1 | 271 | PFAS and THs | maternal age, maternal weight, maternal race, gestational age at blood collection |
| 4 | Inoue et al., 2019 [8] | Cross-sectional | Denmark | N (%)  19-29: 607 (44.4);  30-34: 534 (39.1);  35-45: 225 (16.5) | 1366 | PFAS and THs | maternal age, pre-pregnancy BMI, parity, parental socio-occupational status, maternal smoking, birth year, fish intake in pregnancy, geographical residence, alcohol intake |
| 5 | Itoh et al., 2019 [9] | Cross-sectional | Japan | mean±SD: 30.7±4.4 | 499 | PFAS and THs, TAb subanalysis | age at delivery, pre-pregnancy BMI, parity, educational level, alcohol consumption, maternal smoking during pregnancy |
| 6 | Jenson et al., 2018 [10] | Longitudinal | Denmark | mean±SD: 29.9±4.4 | 318 | PFAS and glucose biomarkers | age, pre-pregnancy BMI, parity, educational level |
| 7 | Lebeaux et al., 2020 [11] | Longitudinal | USA | mean: 30 | 305 | PFAS and THs, TAb subanalysis | maternal age at delivery, maternal pre-pregnancy BMI, race/ethnicity, marital status, maternal education level, household income, mean  log10-transformed cotinine, maternal alcohol usage during pregnancy, nulliparity, fetal sex, gestational week at blood draw for PFAS measurement (if not the same time as TH measurement) |
| 8 | Li et al., 2020 [12] | Longitudinal | China | mean±SD: 28.3±3.2 | 874 | PFAS and GDM/glucose biomarkers | maternal age, maternal pre-pregnancy BMI, maternal education level, annual household income, passive smoking during pregnancy, infant sex, parity |
| 9 | Liu et al., 2019 [13] | Longitudinal | China | mean±SD: 29.3±2.9 | 189 | PFAS and GDM/glucose biomarkers | maternal age, BMI in early pregnancy, fetal sex, serum triglyceride, total cholesterol |
| 10 | Matilla-Santander et al., 2017 [14] | Longitudinal | Spain | mean±SD: 31.9±4 | 1237 | PFAS and GDM/glucose biomarkers | subcohort, country of birth, prepregnancy BMI, previous breastfeeding, parity, physical activity, relative Mediterranean Diet Score, gestational week of blood sample |
| 11 | Mehta et al., 2021 [15] | Cross-sectional | USA | ≤27 years: 48  >27 years: 49 | 95 | PFAS and glucose biomarkers | maternal age at enrollment, race/ethnicity, pre-pregnancy  BMI, household income, parity |
| 12 | Preston et al., 2018 [16] | Longitudinal | USA | mean: 32.5 | 732 | PFAS and THs, TAb subanalysis | maternal age, race/ethnicity, smoking status, fish intake, parity, gestational week at blood draw |
| 13 | Preston et al., 2020 [17] | Longitudinal | USA | mean±SD: 31.9±5.1 | 1540 | PFAS and GDM/glucose biomarkers | prepregnancy BMI, prior history of GDM, parity, smoking, education, maternal age, race/ethnicity |
| 14 | Rahman et al., 2019 [18] | Longitudinal | USA | mean±SD: 28.2 ± 5.5 | 2292 | PFAS and GDM | maternal age, education, parity, race/ethnicity, serum cotinine levels, enrollment BMI, family history of T2D among first degree relatives |
| 15 | Reardon et al., 2019 [19] | Longitudinal | Canada | mean: 33 | 478 | PFAS and THs, TAb subanalysis | maternal age, education, ethnicity, household income, parity, smoking history, current smoking status, alcohol consumption, recreational drug use, diagnosed hyperthyroidism |
| 16 | Ren et al., 2020 [20] | Prospective | China | mean±SD: 27.8±3.3 | 856 | PFAS and glucose biomarkers | maternal age, pre-pregnancy BMI, per capita household income, education level, passive smoking,  parity, pregnancy complication, history of abortion and stillbirth |
| 17 | Shapiro et al., 2016 [21] | Longitudinal | Canada | <=29: 308 (24.2%); 30-34: 447 (35.1%), >=35: 515 (40.4%); Missing: 4 (0.3%) | 1274 | PFAS and GDM/glucose biomarkers | maternal age, race, pre-pregnancy BMI, education |
| 18 | Starling et al., 2017 [22] | Prospective | USA | mean±SD: 27.8±6.2 | 628 | PFAS and glucose biomarkers | maternal age, pre-pregnancy BMI, race/ethnicity, education, smoking during pregnancy, gravidity, gestational age at blood draw |
| 19 | Valvi et al., 2017 [23] | Case-control | Faroe Islands, Denmark | mean±SD: 29.2±5.2 | 604 | PFAS and GDM | maternal age at delivery, pre-pregnancy BMI, education, smoking during pregnancy, parity |
| 20 | Wang et al., 2013 [24] | Cross sectional | Norway | mean±SD: 30±4 | 903 | PFAS and THs | maternal age, parity, gestational age at blood draw*,* total seafood intake, inter-pregnancy interval, HDL levels |
| 21 | Wang et al., 2014 [25] | Cross-sectional | Taiwan | mean±SD: 28.8±4.3 | 285 | PFAS and THs | maternal age, maternal education, parity |
| 22 | Wang et al., 2018 a [26] | Longitudinal | China | 20-40, <=26: 43.4%, 27-28: 25.7%, >=29: 30.9% | 385 | PFAS and GDM/glucose biomarkers | maternal age, pre-pregnancy BMI, paternal smoking status, family per-capita income, fetal sex, average intake of meat, vegetables, and aquatic products, average physical activity, average energy intake, family history of diabetes mellitus |
| 23 | Wang et al., 2018 b [27] | Case-control | China | mean (IQR)  Non-GDM: 29.0 (28.0, 31.0)  GDM mean (IQR): 29.5 (28.0, 32.0) | 252 | PFAS and GDM/glucose biomarkers | BMI, gestational weight gain, ethnic groups, maternal education, parity, maternal drinking during pregnancy, household income |
| 24 | Webster et al., 2014 [28] | Cross-sectional | Canada | mean (range): 34 (25-43) | 152 | PFAS and THs, TAb subanalysis | week of gestation, TPOAb status |
| 25 | Xiao et al., 2020 [29] | Longitudinal | Faroe Islands, Denmark | mean±SD: 28.1±5.6 | 172 | PFAS and THs | maternal pre-pregnancy BMI,  gestational age,  maternal education, parity,  smoking status during pregnancy,  alcohol consumption during pregnancy, fetal sex, potential chemical exposures that may affect TH levels |
| 26 | Xu et al., 2020 [30] | Cross-sectional | China | Controls: mean±SD: 29.4±3.0; GDM: mean±SD: 29.7±3.1 | 495 | PFAS and GDM | maternal age, BMI, parity, sampling time, educational level, serum lipids |
| 27 | Yang et al., 2016 [31] | Longitudinal | China | mean±SD: 29.8±2.9 | 157 | PFAS and THs | maternal age, maternal pre-pregnancy BMI, maternal monthly income, neonatal type of delivery |
| 28 | Zhang et al., 2015 [32] | Longitudinal | USA | mean±SD: 29.7±3.7 | 258 | PFAS and GDM | maternal age, BMI, parity, race/ethnicity, smoking status |

**Abbreviations:** body mass index, BMI; SD, standard deviation; T2D, type 2 diabetes; TAb, thyroid autoantibody; TH, thyroid hormone; TPOAb, thyroid peroxidase antibody

Green: Potential non-confounder variables adjusted for, Red: Potential effect modifiers adjusted for

**SUPPLEMENTAL TABLE 2. Summary of PFAS and biomarker levels in cohorts**

|  | **Author** | **PFAS Levels** | **Glucose Marker Levels** | **TH/TAb Marker Levels** |
| --- | --- | --- | --- | --- |
| 1 | Aimuzi et al., 2020 [5] | Maternal plasma (median (IQR), ng/mL):  PFOA: 12.32 (9.47, 16)  PFOS: 9.25 (6.59, 13.58)  PFNA: 1.63 (1.18, 2.22)  PFDA: 1.60 (1.11, 2.37)  PFUnDA: 1.31 (0.9, 1.88)  PFHxS: 0.54 (0.42, 0.7)  PFDoDA: 0.16 (0.10, 0.24)  PFBS: 0.04 (0.02, 0.06)  PFHpA: 0.06 (0.04, 0.1) | N/A | Maternal serum (median (range)):  FT3 (pmol/l): 4.74 (3.92, 5.88)  TSH (mIU/L): 1.40 (0.15, 3.76)  FT4 (pmol/l): 15.40 (12.21, 19.82)  N(%)  TPOAb(+): 223 (12.1%)  TPOAb(-): 1619 (87.9%) |
| 2 | Berg et al., 2015 [6] | Maternal serum (median, ng/mL):  PFOS: 8.03  PFOA: 1.53  PFNA: 0.56  PFHxS: 0.44  PFUnDA: 0.26  PFDA: 0.23  PFHpS: 0.10 | N/A | Maternal serum (median, range):  TSH (mlU/L): 1.55 (0.06-10.2)  T3 (nmol/L): 2.71 (1.47-4.75)  T4 (nmol/L): 145 (92.00-215)  FT3 (pmol/L): 4.59 (2.99-7.08)  FT4 (pmol/L): 13.0 (9.00-20.0) |
| 3 | Chan et al., 2011 [7] | Maternal serum (GM, ng/mL):  PFOS: 7.39  PFOA: 1.35  PFHxS: 1.08 | N/A | Maternal serum (mean):  Controls  TSH (mU/L): 1.13  FT4 (pmol/L): 12.9  Hypothyroxinemic  TSH (mU/L): 0.69  FT4 (pmol/L): 7.7 |
| 4 | Inoue et al., 2019 [8] | Maternal plasma (median (IQR), ng/mL):  PFOS: 29.5 (22.6, 37.7)  PFOA: 4.52 (3.38, 5.80)  PFHxS: 1.11 (0.83, 1.39)  PFNA: 0.45 (0.36, 0.57) PFHpS: 0.37 (0.27, 0.49)  PFDA: 0.17 (0.13, 0.22) | N/A | Maternal serum:  TSH (mIU/L): 1.13 (1.08,1.19)  FT4 (pmol/L): 14.2 (14.1,14.3) |
| 5 | Itoh et al., 2019 [9] | Maternal plasma (range, ng/mL):  Total PFAS: 3.07-42.67  PFOS: 1.15-30.28  PFOA: <0.2-12.37  PFNA: <0.3-6.64  PFUnDA: <0.1-5.89  PFHxS: <0.2-1.77  PFDA: <0.1-1.59  PFDoDA: <0.1-0.65  PFTrDA: <0.1-1.33  Low detection rates (<40% samples), therefore not included in models:  PFHxA, PFHpA, PFTeDA | N/A | Maternal serum (range):  TSH (μU/mL): <0.005-157.5  FT3 (pg/mL): 1.28-8.75  FT4 (pg/mL): 7.4-44.4  TPOAb (IU/mL): <5.0- 600.0  TgAb (IU/mL): <10.0-382.5 |
| 6 | Jenson et al., 2018 [10] | Maternal serum (median (5^th^-95^th^ percentile), ng/mL):  PFOS: 8.31 (4.08, 16.26)  PFOA: 1.71 (0.69, 4.19)  PFNA: 0.66 (0.37, 1.58)  PFHxS: 0.30 (0.08, 0.60)  PFDA: 0.26 (0.15, 0.53) | Maternal blood (median (IQR)):  Fasting plasma 75g-OGTT glucose (mmol/L): 5.1 (4.8, 5.4)  2h plasma 75g-OGTT glucose (mmol/L): 6.7 (5.7, 7.7)  Fasting serum 75g-OGTT insulin (pmol/L): 79 (51, 112)  Fasting serum 75g-OGTT C-peptide (pmol/L): 766 (603, 1006)  Plasma HOMA-IR: 2.6 (1.6, 3.8)  Plasma HOMA-%β: 140 (106, 187.7)  Plasma IS_OGTT_: 3.7 (2.6, 5.7) | N/A |
| 7 | Lebeaux et al., 2020 [11] | Maternal serum (median (IQR), ng/mL):  Maternal Serum:  PFOS: 14.3 (8.9)  PFOA: 5.5 (4.5)  PFHxS: 1.6 (1.5)  PFNA: 0.9 (0.4) | N/A | Maternal serum (median (IQR)):  TSH (μIU/L): 1.3 (1.2)  T3 (ng/dL): 158.0 (36.0)  T4 (μg/dL): 10.3 (2.5)  FT3 (pg/mL): 3.2 (0.4)  FT4 (ng/dL): 0.7 (0.1) |
| 8 | Li et al., 2020 [12] | Cord blood (GM, ng/mL):  PFOS: 4.16  PFOA: 1.62  6:2 Cl-PFESA: 0.77  PFHxS: 0.4  PFNA: 0.27  PFUnDA: 0.19  PFDA: 0.15  PFTrDA: 0.10  PFBS: 0.04  PFHpA: 0.04  8:2 Cl-PFESA: 0.03  PFDoDA: 0.02  PFTeDA: 0.01  Note: cord blood used in this study to estimate the maternal exposure | Fasting blood glucose, 1h 75g-OGTT, or 2h 75g-OGTT was used to classify women as having GDM (glucose concentrations not listed) | N/A |
| 9 | Liu et al., 2019 [13] | Maternal serum (median (IQR), ng/mL):  PFOS: 4.16 (2.79, 6.39)  Linear-PFOS: 3.13 (2.13, 4.59)  PFOA: 2.29 (1.78, 3.12)  L-PFOA: 2.25 (1.77, 3.06)  PFNA: 0.46 (0.35, 0.57)  PFDA: 0.35 (0.27, 0.49)  5m-PFOS: 0.33 (0.18, 0.59)  PFBA: 0.32 (0.13, 0.41)  PFUnDA: 0.31 (0.23, 0.45)  PFHxS: 0.29 (0.18, 0.45)  6m-PFOS: 0.25 (0.18, 0.43)  4m-PFOS: 0.18 (0.10, 0.34)  3 m-PFOS: 0.14 (0.08, 0.25)  1m-PFOS: 0.13 (0.07, 0.22)  PFPeA: 0.06 (0.03, 0.13)  PFTrDA: 0.05 (0.02, 0.07)  PFDoDA: 0.04 (0.02, 0.06)  6m-PFOA: 0.03 (<LOD, 0.06)  PFHxA: 0.02 (0.01, 0.03)  PFHpA: 0.02 (<LOD, 0.03)  PFBS: 0.005 (<LOD, 0.006) | Maternal plasma (mean (SD)):  75g-OGTT test  Fasting glucose (mmol/L): 4.5 (0.5)  1h glucose (mmol/L): 8.6 (1.7)  2h glucose (mmol/L): 7.3 (1.5) | N/A |
| 10 | Matilla-Santander et al., 2017 [14] | Maternal plasma (GM (GSD), ng/mL):  PFOS: 5.77 (1.61)  PFOA: 2.31 (1.71)  PFHxS: 0.55 (1.96)  PFNA: 0.64 (1.75) | 1h post 50-g OGCT followed by 1h-3h post 100g-OGTT used to classify women as having IGT and GDM (glucose concentrations not listed) | N/A |
| 11 | Mehta et al., 2021 [15] | Maternal serum (GM (GSE), ng/mL):  PFOS: 2.86 (0.17)  PFOA: 1.19 (0.09)  PFNA: 0.57 (0.03)  PFHxS : 0.53 (0.04)  PFDA: 0.17 (0.01) | Maternal blood (GM (GSE)):  FPG (mmol/L): 4.42 (0.04)  Fasting insulin (pmol/L): 81.19 (4.73)  HOMA-IR: 1.65 (0.09) | N/A |
| 12 | Preston et al., 2018 [16] | Maternal plasma (median (IQR), ng/mL):  PFOS: 24.0 (17.6, 32.6)  PFOA: 5.6 (3.9, 7.7)  PFHxS: 2.4 (1.6, 3.8)  NMeFOSAA: 1.8 (1.2, 2.9)  NEtFOSAA: 1.1 (0.7, 1.7)  PFNA: 0.6 (0.5, 0.8) | N/A | Maternal plasma (median (IQR)):  TSH (mIU/mL): 1.2 (0.7, 1.9)  T4 (µg/dL): 9.9 (8.7, 11.2)  FT4 index: 2.1 (1.9, 2.3) |
| 13 | Preston et al., 2020 [17] | Maternal plasma (GM, ng/mL):  PFOS: 25.5  PFOA: 5.7  PFHxS: 2.5  PFNA: 1.9  NMeFOSAA: 1.9  NEtFOSAA: 1.2 | 1h post 50g-OGCT followed by 1h-3h post 100g-OGTT used to classify women as having IH, IGT, and GDM (glucose concentrations not listed) | N/A |
| 14 | Rahman et al., 2019 [18] | Maternal plasma (GM, ng/mL):  PFOS: 5.21 (5.07, 5.35)  PFOA: 1.99 (1.93, 2.04)  PFNA: 0.80 (0.78, 0.82)  PFHxS: 0.76 (0.73, 0.78)  PFDA: 0.27 (0.26, 0.28)  PFUnDA: 0.20 (0.19, 0.20)  NMeFOSAA: 0.09 (0.08, 0.09)  PFHpA: 0.08 (0.07, 0.08)  PFDoDA: 0.06 (0.06, 0.06) | 3h post 100g-OGTT or 2h post 75g-OGTT used to classify women as having GDM (glucose concentrations not listed) | N/A |
| 15 | Reardon et al., 2019 [19] | Maternal plasma (GM, ng/mL):  PFOS: 4.54  Linear PFOS: 2.34  PFOA: 2.12  ∑Br-PFOS: 1.08  PFHxS: 1.01  PFNA: 0.76  iso-PFOS: 0.43  5m-PFOS: 0.32  PFDA: 0.26  ∑3m+4m-PFOS: 0.23  PFUnA: 0.16  1m-PFOS: 0.06  PFHpA: 0.04  PFDoA: 0.03 | N/A | Maternal (GM (GSD)):  First trimester (<13 weeks)  TSH (mIU/L): 1.04 (2.65)  FT4 (pmol/L): 15.1 (1.18)  FT3 (pmol/L): 4.64 (1.16)  Second trimester (14-26 weeks)  TSH (mIU/L): 1.36 (1.96)  FT4 (pmol/L): 14.1 (1.16)  FT3 (pmol/L): 4.54 (1.14)  Third trimester (27-40 weeks)  TSH (mIU/L): 1.20 (1.76)  FT4 (pmol/L): 12.9 (1.24)  FT3 (pmol/L): 4.43 (1.23)  Three months postpartum  TSH (mIU/L): 0.90 (3.20)  FT4 (pmol/L): 14.3 (1.18)  FT3 (pmol/L): 4.16 (1.18) |
| 16 | Ren et al., 2020 [20] | Maternal plasma (GM (GSD), ng/mL):  PFOA: 19.9 (1.6)  PFOS: 10.7 (1.7)  PFHxS: 2.7 (1.5)  PFDA: 2.0 (1.9)  PFNA: 1.8 (1.6)  PFUdA: 1.6 (2.0)  PFDoA: 0.1 (2.2)  PFTrDA: 0.1 (2.1) | Maternal plasma (mean (SD)):  FPG (mmol/L): 4.04 (0.45)  1h-PG (mmol/L): 6.46 (1.37) | N/A |
| 17 | Shapiro et al., 2016 [21] | Maternal plasma (GM (SD), ng/mL):  Normal glucose cases  PFOS: 4.58  PFOA: 1.68  PFHxS: 1.02  Gestational IGT cases  PFOS: 4.29  PFOA: 1.70  PFHxS: 1.0  GDM cases  PFOS: 4.74  PFOA: 1.64  PFHxS: 1.05 | 50g-OGCT followed by 75g-OGTT used to classify women as having IGT and GDM (glucose concentrations not listed) | N/A |
| 18 | Starling et al., 2017 [22] | Maternal serum (GM, ng/mL):  PFOS: 2.30  PFOA: 1.04  PFHxA: 0.75  PFNA: 0.39  PFDA: 0.14 | Maternal plasma (mean (SD)):  FPG (mg/dl): 78:4 (8.8) |  |
| 19 | Valvi et al., 2017 [23] | Maternal serum (Median (IQR), ng/mL):  PFOS: 27.2 (23.1, 33.1)  PFHxS: 4.54 (2.24, 8.52)  PFOA: 3.31 (2.54, 3.99)  PFNA: 0.59 (0.46, 0.79)  PFDA: 0.28 (0.22, 0.38) | 2h post OGTT used to classify women as having GDM (glucose concentrations not listed) | N/A |
| 20 | Wang et al., 2013 [24] | Maternal plasma (GM, ng/mL):  PFOS: 12.77  PFOA: 2.13  PFHxS: 0.62  PFNA: 0.37  PFUnDA: 0.20  PFHpS: 0.12  PFDA: 0.09 | N/A | Maternal plasma (median (IQR)):  TSH (μIU/mL): 3.5 (2.4, 4.8) |
| 21 | Wang et al., 2014 [25] | Maternal serum (median, ng/mL):  PFOS: 12.73  PFUnDA: 3.26  PFOA: 2.39  PFNA: 1.51  PFHxS: 0.81  PFDA: 0.46  PFDoDA: 0.36 | N/A | Maternal serum (median (IQR)):  TSH (μIU/mL): 1.76 (1.19, 2.45)  T4 (μg/dL): 11.16 (9.70, 12.80)  T3 (μg/dL): 0.16 (0.13, 0.18)  FT4 (ng/dL): 0.57 (0.49, 0.69) |
| 22 | Wang et al., 2018 a [26] | Maternal serum (median, ng/mL):  PFOA: 7.3  PFOS: 5.4 | OGTT used to classify women as having GDM  Maternal blood (range):  FBG (mmol/L): 3.06, 6.33  1h glucose post OGTT (mmol/L): 3.32, 14.69  2h glucose post OGTT (mmol/L): 3.42, 12.84  FIns (mIU/L): 2.07, 27.20  HOMA-IR: 0.36, 4.69 | N/A |
| 23 | Wang et al., 2018 b [27] | Maternal serum (median (IQR), ng/mL):  Non-GDM  n-PFOS: 2.81 (1.92, 4.58)  n-PFOA: 1.30 (0.95, 1.83)  PFHxS: 0.47 (0.32, 0.74)  3m+4m-PFOS: 0.42 (0.28, 0.80)  PFNA: 0.36 (0.25, 0.52)  5m-PFOS: 0.36 (0.21, 0.68)  6m-PFOS: 0.31 (0.20, 0.56)  PFDA: 0.27 (0.19, 0.44)  PFUnDA: 0.26 (0.16, 0.39)  1m-PFOS: 0.14 (0.08, 0.24)  GDM  n-PFOS: 2.70 (1.87, 4.17)  n-PFOA: 1.38 (0.98, 1.79)  PFHxS: 0.48 (0.27, 0.75)  3m+4m-PFOS: 0.44 (0.25, 0.68)  PFNA: 0.37 (0.25, 0.45)  5m-PFOS: 0.36 (0.20, 0.60)  6m-PFOS: 0.29 (0.21, 0.51)  PFDA: 0.26 (0.16, 0.44)  PFUnDA: 0.26 (0.15, 0.43)  1m-PFOS: 0.14 (0.08, 0.21) | 75g-OGTT used to classify women as having GDM (glucose concentrations not listed) | N/A |
| 24 | Webster et al., 2014 [28] | Maternal serum (GM (GSD), ng/mL):  PFOS: 4.4 (1.7)  PFOA: 1.6 (1.8)  PFHxS: 1.1 (2.2)  PFNA: 0.6 (1.5)  Low detection rates (<40% samples), therefore not included in models:  PFBS, PFHpS, PFDS, PFPeA, PFHxA, PFHpA, PFDA, PFUnDA, PFDoDA, PFTrDA, PFTA, NEtFOSAA, NMeFOSAA, NEtFOSA, NMeFOSA, PFOSA, FHUEA, FOUEA, FDUEA | N/A | Maternal serum (mean):  15w  TSH (mIU/L): 1.4  T4 (nmol/L): 125.9  FT4 (pmol/L): 9.5  TPOAb (IU/mL): 6.4  18w  TSH (mIU/L): 1.4  T4 (nmol/L): 119.0  FT4 (pmol/L): 8.5 |
| 25 | Xiao et al., 2020 [29] | Maternal serum (GM (GSE), µg/g):  PFOS: 20.86 (0.47)  PFOA: 2.37 (0.07)  NEtFOSAA: 0.65 (0.03)  PFNA: 0.60 (0.02)  PFHxS: 0.55 (0.02)  PFUnDA: 0.47 (0.02)  PFHpS: 0.35 (0.03)  PFDA: 0.30 (0.01)  NMeFOSAAA: 0.18 (0.01)  PFDoDA: 0.03 (<0.01)  PFHpA: 0.03 (<0.01)  FOSA: 0.04 (<0.01) | N/A | Maternal serum (GM (SE)):  TSH (IU/L): 1.34 (0.05)  T4 (nmol/L): 121.08 (1.95)  FT4 (pmol/L): 8.18 (0.11)  FT3: (pmol/L): 4.25 (0.05) |
| 26 | Xu et al., 2020 [30] | Maternal serum (median (IQR), ng/mL):  Controls  PFOA: 7.91 (3.51, 12.9)  PFOS: 6.45 (3.11, 9.2)  PFDA: 1.53 (0.93, 2.31)  PFHxS: 1.33 (0.41, 1.98)  PFUA: 1.09 (0.22, 1.93)  PFNA: 0.83 (0.21, 1.94)  PFBS: 0.13 (0.07, 0.24)  PFDoA: 0.08 (0.02, 0.28)  PFHpS: 0.1 (0.08, 0.25)  PFHpA: 0.07 (<LOD, 0.11)  PFOSA: 0.04 (<LOD, < LOD)  PFDS: 0.04 (<LOD, < LOD)  GDM  PFOA: 8.19 (3.55, 13.19)  PFOS: 6.69 (3.24, 9.42)  PFDA: 1.46 (0.9, 2.27)  PFHxS: 1.33 (0.41, 1.98)  PFUA: 1.13 (0.3, 2.21)  PFNA: 0.77 (0.2, 1.91)  PFDoA: 0.19 (0.04, 0.33)  PFBS: 0.17 (0.09, 0.26)  PFHpA: 0.11 (<LOD, 0.14)  PFHpS: 0.1 (0.08, 0.26)  PFOSA: 0.04 (<LOD, < LOD)  PFDS: 0.04 (<LOD, < LOD) | 1h and 2h post 75g-OGTT used to classify women as having GDM (glucose concentrations not listed) | N/A |
| 27 | Yang et al., 2016 [31] | Maternal serum (mean (SD), ng/mL):  PFHxS: 0.63 (0.47)  PFOS: 5.08 (3.26)  PFOA: 1.95 (1.09)  PFNA: 0.52 (0.27)  PFDA: 0.45 (0.28)  PFUnDA: 0.45 (0.24)  PFDoDA: 0.046 (0.026)  6:2 FTS: 0.04838 (0.04094)  NMeFOSAA: 0.00477 (0.00668) | N/A | Maternal serum (Mean (SD)):  TSH (µIU/L): 3.32 (1.95)  T4 (nmol/L): 145.60 (35.82)  T3 (nmol/L): 2.57 (0.55)  FT4 (pmol/L): 11.98 (2.03)  FT3 (pmol/L): 3.79 (0.67) |
| 28 | Zhang et al., 2015 [32] | Maternal serum (GM (95%CI), ng/mL):  Controls  PFOA: 3.07 (2.83, 3.32)  NEtFOSAA: 0.11 (0.10, 0.12)  NMeFOSAA: 0.29 (0.26, 0.33)  PFDA: 0.40 (0.37, 0.43)  PFNA: 1.20 (1.12, 1.30)  PFOSA: 0.11 (0.10, 0.12)  PFOS: 12.04 (11.12, 13.05)  GDM  PFOA: 3.94 (3.15, 4.93)  NEtFOSAA: 0.11 (0.09, 0.14)  NMeFOSAA: 0.30 (0.21, 0.42)  PFDA: 0.41 (0.32, 0.51)  PFNA: 1.23 (0.99, 1.52)  PFOSA: 0.13 (0.05, 0.34)  PFOS: 13.10 (10.52, 16.33) | Self-reported physician-diagnosed GDM (glucose concentrations not listed) | N/A |

**Abbreviations:** CI, confidence interval; FBG, fasting blood glucose; FIns, fasting insulin; FPG, fasting plasma glucose; GDM, gestational diabetes mellitus; GM, geometric mean; GSD, geometric standard deviation; GSE, geometric standard error; HOMA-IR, homeostatic model assessments of insulin resistance; HOMA-%β, homeostatic model assessments of β-cell function; IH, isolated hyperglycemia; IGT, impaired glucose tolerance; IQR, interquartile range; IS_OGTT_, insulin sensitivity from OGTT (Matsuda index); OGCT, oral glucose challenge test; OGTT, oral glucose tolerance test; 1h-PG, 1h-plasma glucose after 50g-OGTT; SD, standard deviation; SE, standard error; T3, triiodothyronine; T4, thyroxine; TAb, thyroid autoantibody; TgAb, thyroglobulin antibody; TH, thyroid hormone; TPOAb, thyroid peroxidase antibody; TSH, thyrotropin

**SUPPLEMENTAL TABLE 3. Association of PFAS with gestational diabetes mellitus (GDM)**

| **Author** | **Exposure** | **Statistical test (measure of association)** | **Association value (95% CI)** | **Direction of association** |
| --- | --- | --- | --- | --- |
|  |  |  |  |  |
| **Outcome: GDM** | | | | |
| Zhang et al., 2015 | PFOA | Logistic regression (OR) | **1.86 (1.14, 3.02)** | **+** |
| Liu et al., 2019 | Short-chain PFCAs | Conditional logistic regression (OR) | **T1: 1.0 (ref)** | **+** |
|  |  |  | **T2: 1.82 (0.80, 4.16)** |  |
|  |  |  | **T3: 3.01 (1.31, 6.94)** |  |
| Liu et al., 2019 | Linear PFOA | Conditional logistic regression (OR) | **T1: 1.0 (ref)** | **+** |
|  |  |  | **T2: 1.04 (0.47, 2.34)** |  |
|  |  |  | **T3: 2.04 (0.94, 4.46)** |  |
| Xu et al., 2020 | PFDoDA | Multivariable conditional logistic regression (OR) | **Q1: 1.0 ref** | **+** |
|  |  |  | **Q2: 0.77 (0.50, 1.78)** |  |
|  |  |  | **Q3: 1.99 (1.10, 3.23)** |  |
|  |  |  | **Q4: 13.02 (4.71, 27.28)** |  |
| Rahman et al., 2019 | PFHpA | Multivariate Poisson regression (RR) | **1.18 (1.04, 1.34)** | **+** |
| Xu et al., 2020 | PFBS | Multivariable conditional logistic regression (OR) | **Q1: 1.0 (ref)** | **+** |
|  |  |  | **Q2: 1.23 (1.02, 1.99)** |  |
|  |  |  | **Q3: 1.30 (1.25, 2.17)** |  |
|  |  |  | **Q4: 1.97 (1.09, 3.42)** |  |
| Matilla-Santander et al., 2017 | PFOA | Logistic regression, per log10-unit increase of PFAS (OR) | 1.20 (0.62, 2.30) | + |
| Preston et al., 2020 | PFOA | Multinomial logistic regression (OR) | Q1: 1.0 (ref) | + |
|  |  |  | Q2: 1.1 (0.5, 2.3) |  |
|  |  |  | Q3: 1.3 (0.6, 2.8) |  |
|  |  |  | Q4: 1.4 (0.7, 2.9) |  |
| Xu et al., 2020 | PFOA | Multivariable conditional logistic regression (OR) | Q1: 1.0 (ref) | + |
|  |  |  | Q2: 1.05 (0.45, 2.04) |  |
|  |  |  | Q3: 1.12 (0.46, 2.20) |  |
|  |  |  | Q4: 1.20 (0.28, 2.21) |  |
| Wang et al., 2018 b | n-PFOA | Conditional logistic regression (OR) | 1.31 (0.95, 1.80) | + |
| Matilla-Santander et al., 2017 | PFOS | Logistic regression, per log10-unit increase of PFAS (OR) | 2.40 (0.93, 6.18) | + |
| Zhang et al., 2015 | PFOS | Logistic regression (OR) | 1.13 (0.75, 1.72) | + |
| Preston et al., 2020 | PFOS | Multinomial logistic regression (OR) | Q1: (ref) | + |
|  |  |  | Q2: 1.1 (0.5, 2.4) |  |
|  |  |  | Q3: 1.0 (0.5, 2.2) |  |
|  |  |  | Q4: 1.5 (0.7, 3.0) |  |
| Wang et al., 2018 a | PFOS | Cox proportional hazard regression model (HR) | 2.11 (0.76, 5.86) | + |
| Liu et al., 2019 | Linear PFOS | Conditional logistic regression (OR) | T1: 1.0 (ref) | + |
|  |  |  | T2: 1.34 (0.62, 2.93) |  |
|  |  |  | T3: 1.37 (0.62, 3.02) |  |
| Liu et al., 2019 | Σm-PFOS | Conditional logistic regression (OR) | T1: (ref) | + |
|  |  |  | T2: 1.53 (0.70, 3.34) |  |
|  |  |  | T3: 1.23 (0.56, 2.72) |  |
| Li et al., 2020 | PFDA | Logistic regression (OR) | 1.18 (0.84, 1.65) | + |
| Valvi et al., 2017 | PFDA | Logistic regression, per doubling of exposure (OR) | 1.20 (0.73, 1.96) | + |
| Li et al., 2020 | PFNA | Logistic regression (OR) | 1.40 (0.92, 2.14) | + |
| Wang et al., 2018 b | PFNA | Conditional logistic regression (OR) | 1.25 (0.37, 4.28) | + |
| Matilla-Santander et al., 2017 | PFHxS | Logistic regression, per log10-unit increase of PFAS (OR) | 1.58 (0.73, 3.44) | + |
| Preston et al., 2020 | PFHxS | Multinomial logistic regression (OR) | Q1: 1.0 (ref) | + |
|  |  |  | Q2: 1.1 (0.6, 2.3) |  |
|  |  |  | Q3: 1.2 (0.6, 2.4) |  |
|  |  |  | Q4: 1.0 (0.5, 2.2) |  |
| Shapiro et al., 2016 | PFHxS | Logistic regression (OR) | Q1: 1.0 (ref) | + |
|  |  |  | Q2: 1.6 (0.7, 3.8) |  |
|  |  |  | Q3: 1.4 (0.6, 3.5) |  |
|  |  |  | Q4: 1.2 (0.4, 3.5) |  |
| Li et al., 2020 | PFUnDA | Logistic regression (OR) | 1.12 (0.78, 1.61) | + |
| Wang et al., 2018 b | PFUnDA | Conditional logistic regression (OR) | 1.79 (0.65, 4.96) | + |
| Li et al., 2020 | PFTrDA | Logistic regression (OR) | 1.16 (0.80, 1.68) | + |
| Preston et al., 2020 | NEtFOSAA | Multinomial logistic regression (OR) | Q1: 1.0 (ref) | + |
|  |  |  | Q2: 1.4 (0.7, 2.8) |  |
|  |  |  | Q3: 1.4 (0.7, 3.0) |  |
|  |  |  | Q4: 1.5 (0.7, 3.2) |  |
| Zhang et al., 2015 | NEtFOSAA | Logistic regression (OR) | 1.25 (0.87, 1.80) | + |
| Liu et al., 2019 | Long-chain PFCAs | Conditional logistic regression (OR) | T1: 1.0 (ref) | + |
|  |  |  | T2: 1.24 (0.56, 2.76) |  |
|  |  |  | T3: 1.88 (0.85, 4.15) |  |
| Liu et al., 2019 | Total PFCAs | Conditional logistic regression (OR) | T1: 1.0 (ref) | + |
|  |  |  | T2: 2.22 (0.96, 5.12) |  |
|  |  |  | T3: 2.47 (1.06, 5.74) |  |
| Wang et al., 2018 a | PFOA | Cox proportional hazard regression model (HR) | 0.71 (0.29, 1.75) | - |
| Rahman et al., 2019 | PFOS | Multivariate Poisson regression (RR) | 0.89 (0.69, 1.16) | - |
| Valvi et al., 2017 | PFOS | Logistic regression, per doubling of exposure (OR) | 0.86 (0.43, 1.70) | - |
| Wang et al., 2018 b | 1m-PFOS | Conditional logistic regression (OR) | 0.71 (0.14, 3.55) | - |
| Wang et al., 2018 b | 3m+4m-PFOS | Conditional logistic regression (OR) | 0.74 (0.44, 1.22) | - |
| Wang et al., 2018 b | 5m-PFOS | Conditional logistic regression (OR) | 0.74 (0.41, 1.35) | - |
| Wang et al., 2018 b | 6m-PFOS | Conditional logistic regression (OR) | 0.72 (0.32, 1.67) | - |
| Valvi et al., 2017 | PFOA | Logistic regression, per doubling of exposure (OR) | 0.79 (0.44, 1.41) | - |
| Matilla-Santander et al., 2017 | PFNA | Logistic regression, per log10-unit increase of PFAS (OR) | 0.85 (0.40, 1.80) | - |
| Valvi et al., 2017 | PFNA | Logistic regression, per doubling of exposure (OR) | 0.88 (0.53, 1.47) | - |
| Wang et al., 2018 b | PFDA | Conditional logistic regression (OR) | 0.85 (0.30, 2.92) | - |
| Xu et al., 2020 | PFHxS | Multivariable conditional logistic regression (OR) | Q1: 1.0 (ref) | - |
|  |  |  | Q2: 0.71 (0.25, 1.65) |  |
|  |  |  | Q3: 0.90 (0.39, 2.04) |  |
|  |  |  | Q4: 0.81 (0.21, 1.64) |  |
| Rahman et al., 2019 | PFDoDA | Multivariate Poisson regression (RR) | 0.87 (0.42, 1.80) | - |
| Rahman et al., 2019 | PFUnDA | Multivariate Poisson regression (RR) | 0.88 (0.67, 1.17) | - |
| Li et al., 2020 | 8:2 Cl-PFESA | Logistic regression (OR) | 0.86 (0.65, 1.14) | - |
| Rahman et al., 2019 | Total PFAS | Multivariate Poisson regression (RR) | 0.95 (0.73, 1.25) | null |
| Li et al., 2020 | PFOA | Logistic regression (OR) | 1.00 (0.61, 1.65) | null |
| Rahman et al., 2019 | PFOA | Multivariate Poisson regression (RR) | 1.07 (0.86, 1.34) | null |
| Shapiro et al., 2016 | PFOA | Logistic regression (OR) | Q1: 1.0 (ref) | null |
|  |  |  | Q2: 0.9 (0.4, 2.1) |  |
|  |  |  | Q3:1.0 (0.4, 2.2) |  |
|  |  |  | Q4: 0.9 (0.3, 2.3) |  |
| Liu et al., 2019 | Σm-PFOA | Conditional logistic regression (OR) | T1: 1.0 (ref) | null |
|  |  |  | T2: 0.91 (0.40, 2.07) |  |
|  |  |  | T3: 2.01 (0.92, 4.37) |  |
| Li et al., 2020 | PFOS | Logistic regression (OR) | 0.93 (0.72, 1.22) | null |
| Shapiro et al., 2016 | PFOS | Logistic regression (OR) | Q1: 1.0 (ref) | null |
|  |  |  | Q2: 0.6 (0.3, 1.6) |  |
|  |  |  | Q3: 1.1 (0.5, 2.5) |  |
|  |  |  | Q4: 0.7 (0.3, 1.7) |  |
| Xu et al., 2020 | PFOS | Multivariable conditional logistic regression (OR) | Q1: (ref) | null |
|  |  |  | Q2: 0.69 (0.34, 2.07) |  |
|  |  |  | Q3: 0.72 (0.48, 1.90) |  |
|  |  |  | Q4: 1.07 (0.51, 1.32) |  |
| Wang et al., 2018 b | n-PFOS | Conditional logistic regression (OR) | 0.96 (0.85, 1.09) | null |
| Preston et al., 2020 | PFNA | Multinomial logistic regression (OR) | Q1: 1.0 (ref) | null |
|  |  |  | Q2: 1.4 (0.8, 2.6) |  |
|  |  |  | Q3: 0.8 (0.4, 1.9) |  |
|  |  |  | Q4: 1.0 (0.5, 2.0) |  |
| Rahman et al., 2019 | PFNA | Multivariate Poisson regression (RR) | 1.05 (0.82, 1.35) | null |
| Xu et al., 2020 | PFNA | Multivariable conditional logistic regression (OR) | Q1: 1.0 (ref) | null |
|  |  |  | Q2: 1.01 (0.57, 2.04) |  |
|  |  |  | Q3: 1.14 (0.60, 1.90) |  |
|  |  |  | Q4: 0.71 (0.43, 1.35) |  |
| Zhang et al., 2015 | PFNA | Logistic regression (OR) | 1.06 (0.70, 1.60) | null |
| Rahman et al., 2019 | PFDA | Multivariate Poisson regression (RR) | 1.02 (0.86, 1.20) | null |
| Xu et al., 2020 | PFDA | Multivariable conditional logistic regression (OR) | Q1: 1.0 (ref) | null |
|  |  |  | Q2: 0.71 (0.29, 1.46) |  |
|  |  |  | Q3: 1.01 (0.42, 2.71) |  |
|  |  |  | Q4: 1.14 (0.53, 2.07) |  |
| Li et al., 2020 | PFHxS | Logistic regression (OR) | 0.98 (0.67, 1.41) | null |
| Rahman et al., 2019 | PFHxS | Multivariate Poisson regression (RR) | 0.95 (0.73, 1.23) | null |
| Valvi et al., 2017 | PFHxS | Logistic regression, per doubling of exposure (OR) | 1.03 (0.80, 1.33) | null |
| Wang et al., 2018 b | PFHxS | Conditional logistic regression (OR) | 1.07 (0.86, 1.35) | null |
| Li et al., 2020 | PFDoDA | Logistic regression (OR) | 1.09 (0.81, 1.68) | null |
| Xu et al., 2020 | PFUnDA | Multivariable conditional logistic regression (OR) | Q1: 1.0 (ref) | null |
|  |  |  | Q2: 0.81 (0.41, 2.03) |  |
|  |  |  | Q3: 0.71 (0.31, 1.94) |  |
|  |  |  | Q4: 1.01 (0.37, 2.53) |  |
| Preston et al., 2020 | NMeFOSAA | Multinomial logistic regression (OR) | Q1: 1.0 (ref) | null |
|  |  |  | Q2: 1.1 (0.6, 2.2) |  |
|  |  |  | Q3: 1.3 (0.7, 2.4) |  |
|  |  |  | Q4: 0.9 (0.4, 1.9) |  |
| Zhang et al., 2015 | NMeFOSAA | Logistic regression (OR) | 1.05 (0.71, 1.54) | null |
| Rahman et al., 2019 | NMeFOSAA | Multivariate Poisson regression (RR) | 1.01 (0.85, 1.19) | null |
| Li et al., 2020 | PFHpA | Logistic regression (OR) | 1.08 (0.63, 1.87) | null |
| Li et al., 2020 | PFTeDA | Logistic regression (OR) | 0.92 (0.62, 1.37) | null |
| Li et al., 2020 | PFBS | Logistic regression (OR) | 1.04 (0.84, 1.29) | null |
| Li et al., 2020 | 6:2 Cl-PFESA | Logistic regression (OR) | 1.02 (0.76, 1.37) | null |
| Zhang et al., 2015 | PFDA | Logistic regression (OR) | 1.04 (0.70, 1.53) | null |
| Zhang et al., 2015 | PFOSA | Logistic regression (OR) | 1.07 (0.74, 1.55) | null |
| Xu et al., 2020 | PFHpS | Multivariable conditional logistic regression (OR) | Q1: 1.0 (ref) | nonmonotonic |
|  |  |  | Q2: 0.97 (0.50, 1.98) |  |
|  |  |  | Q3: 0.82 (0.41, 1.91) |  |
|  |  |  | Q4: 1.19 (0.61, 2.70) |  |
| Liu et al., 2019 | Short-chain PFSAs | Conditional logistic regression (OR) | T1: 1.0 (ref) | nonmonotonic |
|  |  |  | T2: 1.31 (0.65, 2.64) |  |
|  |  |  | T3: 1.13 (0.47, 2.74) |  |
| Liu et al., 2019 | Long-chain PFSAs | Conditional logistic regression (OR) | T1: 1.0 (ref) | nonmonotonic |
|  |  |  | T2: 1.78 (0.75, 4.25) |  |
|  |  |  | T3: 1.39 (0.55, 3.49) |  |
| Liu et al., 2019 | Total PFSAs | Conditional logistic regression (OR) | T1: 1.0 (ref) | nonmonotonic |
|  |  |  | T2: 1.54 (0.71, 3.35) |  |
|  |  |  | T3: 1.25 (0.57, 2.76) |  |

**Abbreviations:** HR, hazard ratio; IDE, indeterminate, GDM, gestational diabetes mellitus; OR, odds ratio; Q, quartile; ref, reference; RR, risk ratio; T, tertile

**Bold**: statistically significant

**SUPPLEMENTAL TABLE 4. Association of PFAS with glucose and insulin biomarkers**

| **Author** | **Exposure** | **Statistical test (measure of association)** | **Association value (95% CI)** | **Direction of association** |
| --- | --- | --- | --- | --- |
|  |  |  |  |  |
| **Outcome: IH** | | | | |
| Preston et al., 2020 | PFHxS | Multinomial logistic regression (OR) | Q1: 1.0 (ref) | + |
|  |  |  | Q2: 1.0 (0.6, 1.7) |  |
|  |  |  | Q3: 1.2 (0.7, 2.0) |  |
|  |  |  | Q4: 1.4 (0.8, 2.5) |  |
| Preston et al., 2020 | PFOA | Multinomial logistic regression (OR) | Q1: 1.0 (ref) | - |
|  |  |  | Q2: 0.8 (0.5, 1.4) |  |
|  |  |  | Q3: 0.8 (0.5, 1.4) |  |
|  |  |  | Q4: 0.9 (0.5, 1.6) |  |
| Preston et al., 2020 | PFNA | Multinomial logistic regression (OR) | Q1: 1.0 (ref) | null |
|  |  |  | Q2: 0.9 (0.5, 1.4) |  |
|  |  |  | Q3: 1.0 (0.6, 1.7) |  |
|  |  |  | Q4: 1.1 (0.6, 1.8) |  |
| Preston et al., 2020 | NEtFOSAA | Multinomial logistic regression (OR) | Q1: 1.0 (ref) | null |
|  |  |  | Q2: 0.7 (0.4, 1.2) |  |
|  |  |  | Q3: 1.0 (0.6, 1.6) |  |
|  |  |  | Q4: 0.9 (0.5, 1.5) |  |
| Preston et al., 2020 | PFOS | Multinomial logistic regression (OR) | Q1: 1.0 (ref) | nonmonotonic |
|  |  |  | Q2: 1.1 (0.7, 1.9) |  |
|  |  |  | Q3: 1.3 (0.7, 2.1) |  |
|  |  |  | Q4: 1.0 (0.6, 1.7) |  |
| Preston et al., 2020 | NMeFOSAA | Multinomial logistic regression (OR) | Q1: 1.0 ref | nonmonotonic |
|  |  |  | Q2: 1.6 (1.0, 2.7) |  |
|  |  |  | Q3: 0.9 (0.5, 1.6) |  |
|  |  |  | Q4: 1.2 (0.7, 2.0) |  |
| **Outcome: IGT** | | | | |
| Matilla-Santander et al., 2017 | PFOS | Logistic regression, per log10-unit increase of PFAS (OR) | **1.99 (1.06, 3.78)** | **+** |
| Shapiro et al., 2016 | PFHxS | Logistic regression (OR) | Q1: 1.0 (ref) | **+** |
|  |  |  | **Q2: 3.5 (1.4, 8.9)** |  |
|  |  |  | Q3: 1.3 (0.5, 4.0) |  |
|  |  |  | Q4: 2.5 (0.9, 7.0) |  |
| Matilla-Santander et al., 2017 | PFOA | Logistic regression, per log10-unit increase of PFAS (OR) | 1.24 (0.78, 1.94) | + |
| Matilla-Santander et al., 2017 | PFHxS | Logistic regression, per log10-unit increase of PFAS (OR) | 1.65 (0.99, 2.76) | + |
| Preston et al., 2020 | NMeFOSAA | Multinomial logistic regression (OR) | Q1: 1.0 (ref) | + |
|  |  |  | Q2: 2.0 (0.9, 4.9) |  |
|  |  |  | Q3: 1.9 (0.8, 4.4) |  |
|  |  |  | Q4: 1.0 (0.4, 2.8) |  |
| Preston et al., 2020 | PFOA | Multinomial logistic regression (OR) | Q1: 1.0 (ref) | - |
|  |  |  | Q2: 0.6 (0.3, 1.4) |  |
|  |  |  | Q3: 0.6 (0.2, 1.5) |  |
|  |  |  | Q4: 0.8 (0.3, 1.8) |  |
| Shapiro et al., 2016 | PFOA | Logistic regression (OR) | Q1: 1.0 (ref) | - |
|  |  |  | Q2: 0.9 (0.4, 2.0) |  |
|  |  |  | Q3: 0.7 (0.3, 1.6) |  |
|  |  |  | Q4: 0.7 (0.3, 1.8) |  |
| Preston et al., 2020 | PFNA | Multinomial logistic regression (OR) | Q1: 1.0 (ref) | - |
|  |  |  | Q2: 0.6 (0.3, 1.3) |  |
|  |  |  | Q3: 0.5 (0.2, 1.5) |  |
|  |  |  | Q4: 0.9 (0.4, 2.0) |  |
| Matilla-Santander et al., 2017 | PFNA | Logistic regression, per log10-unit increase of PFAS (OR) | 0.95 (0.57, 1.60) | null |
| Preston et al., 2020 | PFHxS | Multinomial logistic regression (OR) | Q1: 1.0 (ref) | null |
|  |  |  | Q2: 0.7 (0.3, 1.8) |  |
|  |  |  | Q3: 1.3 (0.6, 2.8) |  |
|  |  |  | Q4: 1.0 (0.4, 2.5) |  |
| Preston et al., 2020 | NEtFOSAA | Multinomial logistic regression (OR) | Q1: 1.0 (ref) | null |
|  |  |  | Q2: 0.9 (0.4, 2.2) |  |
|  |  |  | Q3: 1.2 (0.5, 2.8) |  |
|  |  |  | Q4: 1.1 (0.4, 2.5) |  |
| Preston et al., 2020 | PFOS | Multinomial logistic regression (OR) | Q1: 1.0 (ref) | nonmonotonic |
|  |  |  | Q2: 1.2 (0.5, 3.0) |  |
|  |  |  | Q3: 1.9 (0.8, 4.4) |  |
|  |  |  | Q4: 0.9 (0.3, 2.4) |  |
| Shapiro et al., 2016 | PFOS | Logistic regression (OR) | Q1: 1.0 (ref) | nonmonotonic |
|  |  |  | Q2: 1.2 (0.5, 2.8) |  |
|  |  |  | Q3: 1.2 (0.6, 2.8) |  |
|  |  |  | Q4: 0.8 (0.3, 2.1) |  |
| **Outcome: Fasting blood glucose** | | | | |
| Li et al., 2020 | PFHpA | Multivariable linear regression (β) | **0.07 (0.02, 0.13)** | **+** |
| Wang et al., 2018 b | 1m-PFOS | Multinomial logistic regression analysis (OR) | Low blood glucose group: (ref) |  |
|  |  |  | **Medium blood glucose group: 1.86 (1.00, 3.48)** | **+** |
|  |  |  | **High blood glucose group: 2.07 (1.09, 3.93)** | **+** |
| Wang et al., 2018 b | 3m+4m-PFOS | Multinomial logistic regression analysis (OR) | Low blood glucose group: (ref) |  |
|  |  |  | Medium blood glucose group: 1.81 (0.98, 3.33) | + |
|  |  |  | **High blood glucose group: 1.88 (1.00, 3.52)** | **+** |
| Wang et al., 2018 b | 5m-PFOS | Multinomial logistic regression analysis (OR) | Low blood glucose group: (ref) |  |
|  |  |  | **Medium blood glucose group: 1.94 (1.05, 3.80)** | **+** |
|  |  |  | **High blood glucose group: 2.45 (1.24, 4.64)** | **+** |
| Wang et al., 2018 b | n-PFOS | Multinomial logistic regression analysis (OR) | Low blood glucose group: (ref) |  |
|  |  |  | **Medium blood glucose group: 1.94 (1.05, 3.58)** | **+** |
|  |  |  | High blood glucose group: 1.59 (0.85, 2.96) | + |
| Jensen et al., 2018 | PFHxS | Linear regression (β, % change) | **High GDM risk: 1.7 (0.2, 3.2)** | **+** |
|  |  |  | Low GDM risk: 0.2 (-1.3, 1.7) | null |
| Wang et al., 2018 b | PFHxS | Multinomial logistic regression analysis (OR) | Low blood glucose group: (ref) |  |
|  |  |  | **Medium blood glucose group: 1.32 (0.72, 2.42)** | **+** |
|  |  |  | **High blood glucose group: 2.29 (1.22, 4.29)** | **+** |
| Wang et al., 2018 b | n-PFOA | Multinomial logistic regression analysis (OR) | Low blood glucose group: (ref) |  |
|  |  |  | Medium blood glucose group: 1.60 (0.87, 2.93) | + |
|  |  |  | High blood glucose group: 1.33 (0.72, 2.48) | + |
| Ren et al., 2020 | PFOA | Logistic regression (OR) | 1.31 (0.77, 2.22) | + |
| Wang et al., 2018 b | 6m-PFOS | Multinomial logistic regression analysis (OR) | Low blood glucose group: (ref) |  |
|  |  |  | Medium blood glucose group: 1.24 (0.67, 2.28) | + |
|  |  |  | High blood glucose group: 1.42 (0.83, 2.77) | + |
| Ren et al., 2020 | PFOS | Logistic regression (OR) | 1.28 (0.85, 1.93) | + |
| Wang et al., 2018 b | PFNA | Multinomial logistic regression analysis (OR) | Low blood glucose group: (ref) |  |
|  |  |  | Medium blood glucose group: 1.37 (0.74, 2.52) | + |
|  |  |  | High blood glucose group: 1.08 (0.58, 2.00) | null |
| Ren et al., 2020 | PFNA | Logistic regression (OR) | 1.55 (0.98, 2.46) | + |
| Wang et al., 2018 b | PFDA | Multinomial logistic regression analysis (OR) | Low blood glucose group: (ref) |  |
|  |  |  | Medium blood glucose group: 1.30 (0.71, 2.38) | + |
|  |  |  | High blood glucose group: 0.96 (0.52, 1.78) | null |
| Ren et al., 2020 | PFDA | Logistic regression (OR) | 1.24 (0.87, 1.76) | + |
| Ren et al., 2020 | PFTrDA | Logistic regression (OR) | 1.12 (0.89, 1.40) | + |
| Mehta et al., 2021 | ∑PFAS | Multivariable linear regression, per doubling of exposure (β, % change) | **-2.73 (-5.15, -0.25)** | **-** |
| Mehta et al., 2021 | PFOS | Multivariable linear regression, per doubling of exposure (β, % change) | **-2.06 (-4.06, -0.02)** | **-** |
| Starling et al., 2017 | PFDA | Linear regression (β) | Low PFDA group: 1.0 (ref) | **-** |
|  |  |  | **High PFDA group: -0.024 (-0.041, -0.007)** |  |
| Starling et al., 2017 | PFNA | Linear regression (β) | Low PFNA group: 1.0 (ref) | **-** |
|  |  |  | **High PFNA group: -0.025 (-0.042, -0.009)** |  |
| Starling et al., 2017 | PFHxS | Linear regression (β) | T1: 1.0 (ref) | **-** |
|  |  |  | T2: -0.009 (-0.029, 0.010) |  |
|  |  |  | **T3: -0.023 (-0.044, -0.002)** |  |
| Ren et al., 2020 | PFHxS | Logistic regression (OR) | 0.89 (0.51, 1.55) | - |
| Wang et al., 2018 b | PFUnDA | Multinomial logistic regression analysis (OR) | Low blood glucose group: (ref) |  |
|  |  |  | Medium blood glucose group: 0.77 (0.42, 1.41) | - |
|  |  |  | High blood glucose group: 0.71 (0.38, 1.73) | - |
| Ren et al., 2020 | PFUnDA | Logistic regression (OR) | 0.98 (0.71, 1.35) | null |
| Jensen et al., 2018 | PFOA | Linear regression (β, % change) | High GDM risk: -1.3 (-3.0, 0.5) | null |
|  |  |  | Low GDM risk: 1.2 (-0.7, 3.2) | null |
| Li et al., 2020 | PFOA | Multivariable linear regression (β) | -0.02 (-0.07, 0.03) | IDE |
| Mehta et al., 2021 | PFOA | Multivariable linear regression, per doubling of exposure (β, % change) | -1.38 (-2.85, 0.12) | null |
| Starling et al., 2017 | PFOA | Linear regression (β) | T1: 1.0 (ref) | IDE |
|  |  |  | T2: -0.014 (-0.034, 0.006) |  |
|  |  |  | T3: -0.025 (-0.046, -0.004) |  |
| Wang et al., 2018 a | PFOA | Linear regression (β) | -0.005 (-0.018, 0.008) | IDE |
| Liu et al., 2019 | Linear PFOA | General linear models, (β) | 0.07 (-0.08, 0.21) | IDE |
| Liu et al., 2019 | Σm-PFOA | General linear models, (β) | 0.03 (-0.03, 0.08) | IDE |
| Jensen et al., 2018 | PFOS | Linear regression (β, % change) | High GDM risk: -0.1 (-2.3, 2.2) | null |
|  |  |  | Low GDM risk: 0.3 (-2.2, 2.8) |  |
| Li et al., 2020 | PFOS | Multivariable linear regression (β) | 0.01 (-0.03, 0.03) | IDE |
| Starling et al., 2017 | PFOS | Linear regression (β) | T1: 1.0 (ref) | IDE |
|  |  |  | T2: 0.011 (-0.009, 0.030), |  |
|  |  |  | T3: -0.009 (-0.029, 0.011) |  |
| Wang et al., 2018 a | PFOS | Linear regression (β) | -0.009 (-0.019, 0.002) | IDE |
| Liu et al., 2019 | Linear PFOS | General linear models, (β) | -0.07 (-0.17, 0.04) | IDE |
| Liu et al., 2019 | Σm-PFOS | General linear models, (β) | -0.02 (-0.11, 0.06) | IDE |
| Jensen et al., 2018 | PFNA | Linear regression (β, % change) | High GDM risk: 0.03 (-2.1, 2.2) | null |
|  |  |  | Low GDM risk: 1.4 (-0.8, 3.7) |  |
| Li et al., 2020 | PFNA | Multivariable linear regression (β) | 0.00 (-0.04, 0.04) | IDE |
| Mehta et al., 2021 | PFNA | Multivariable linear regression, per doubling of exposure (β, % change) | -2.06 (-4.24, 0.17) | null |
| Jensen et al., 2018 | PFDA | Linear regression (β, % change) | High GDM risk: -1.3 (-3.6, 1.0) | null |
|  |  |  | Low GDM risk: 0.5 (-1.9, 2.9) |  |
| Li et al., 2020 | PFDA | Multivariable linear regression (β) | -0.02 (-0.06, 0.01) | IDE |
| Mehta et al., 2021 | PFDA | Multivariable linear regression, per doubling of exposure (β, % change) | -0.69 (-2.12, 0.76) | null |
| Li et al., 2020 | PFHxS | Multivariable linear regression (β) | 0.00 (-0.04, 0.04) | IDE |
| Mehta et al., 2021 | PFHxS | Multivariable linear regression, per doubling of exposure (β, % change) | -0.69 (-2.18, 0.83) | null |
| Li et al., 2020 | PFDoDA | Multivariable linear regression (β) | -0.02 (-0.06, 0.01) | IDE |
| Ren et al., 2020 | PFDoDA | Logistic regression (OR) | 1.09 (0.87, 1.37) | null |
| Li et al., 2020 | PFUnDA | Multivariable linear regression (β) | -0.02 (-0.06, 0.01) | IDE |
| Li et al., 2020 | PFTrDA | Multivariable linear regression (β) | -0.02 (-0.06, 0.00) | IDE |
| Li et al., 2020 | PFTeDA | Multivariable linear regression (β) | -0.03 (-0.07, 0.01) | IDE |
| Li et al., 2020 | PFBS | Multivariable linear regression (β) | 0.00 (-0.02, 0.02) | IDE |
| Li et al., 2020 | 6:2 Cl-PFESA | Multivariable linear regression (β) | 0.00 (-0.03, 0.03) | IDE |
| Li et al., 2020 | 8:2 Cl-PFESA | Multivariable linear regression (β) | -0.01 (-0.04, 0.03) | IDE |
| Liu et al., 2019 | Short-chain PFCAs | General linear models, (β) | 0.002 (-0.07, 0.07) | IDE |
| Liu et al., 2019 | Long-chain PFCAs | General linear models, (β) | -0.01 (-0.17, 0.15) | IDE |
| Liu et al., 2019 | Short-chain PFSAs | General linear models, (β) | -0.01 (-0.12, 0.09) | IDE |
| Liu et al., 2019 | Long-chain PFSAs | General linear models, (β) | -0.05 (-0.16, 0.05) | IDE |
| Liu et al., 2019 | Total PFCAs | General linear models, (β) | -0.01 (-0.18, 0.15) | IDE |
| Liu et al., 2019 | Total PFSAs | General linear models, (β) | -0.06 (-0.16, 0.05) | IDE |
| **Outcome: 1-hr glucose test** | | | | |
| Liu et al., 2019 | Linear PFOA | General linear models, (β) | **0.55 (0.01, 1.10)** | **+** |
| Ren et al., 2020 | PFOS | Logistic regression (OR) | **1.87 (1.15, 3.05)** | **+** |
| Ren et al., 2020 | PFNA | Logistic regression (OR) | **2.15 (1.24, 3.74)** | **+** |
| Ren et al., 2020 | PFDA | Logistic regression (OR) | **1.61 (1.10, 2.44)** | **+** |
| Ren et al., 2020 | PFUnDA | Logistic regression (OR) | **1.71 (1.12, 2.62)** | **+** |
| Ren et al., 2020 | PFDoDA | Logistic regression (OR) | **1.34 (1.00, 1.81)** | **+** |
| Li et al., 2020 | PFBS | Multivariable linear regression (β) | **0.09 (0.02, 0.17)** | **+** |
| Liu et al., 2019 | Short-chain PFCAs | General linear models, (β) | **0.42 (0.14, 0.69)** | **+** |
| Liu et al., 2019 | Total PFCAs | General linear models, (β) | **0.73 (0.10, 1.37)** | **+** |
| Preston et al., 2020 | PFOS | Multinomial logistic regression (OR) | Q1: 0 (ref) | **+** |
|  |  |  | Q2: 2.4 (-1.2, 6.0) |  |
|  |  |  | **Q3: 3.7 (0.0, 7.4)** |  |
|  |  |  | **Q4: 4.3 (0.5, 8.0)** |  |
| Preston et al., 2020 | NMeFOSAA | Multinomial logistic regression (OR) | Q1: (ref) | **+** |
|  |  |  | **Q2: 5.8 (2.1, 9.4)** |  |
|  |  |  | Q3: 2.4 (-1.1, 5.9) |  |
|  |  |  | Q4: 2.7 (-1.0, 6.4) |  |
| Ren et al., 2020 | PFTrDA | Logistic regression (OR) | 1.13 (0.87, 1.49) | + |
| Ren et al., 2020 | PFOA | Logistic regression (OR) | 1.40 (0.75, 2.59) | + |
| Li et al., 2020 | PFOA | Multivariable linear regression (β) | 0.03 (-0.15, 0.20) | IDE |
| Preston et al., 2020 | PFOA | Multinomial logistic regression, (OR) | Q1: 0 (ref) | null |
|  |  |  | Q2: -0.4 (-4.0, 3.3) |  |
|  |  |  | Q3: 1.0 (-2.8, 4.8) |  |
|  |  |  | Q4: 1.5 (-2.4, -5.3) |  |
| Liu et al., 2019 | Σm-PFOA | General linear models, (β) | -0.01 (-0.24, 0.21) | IDE |
| Li et al., 2020 | PFOS | Multivariable linear regression (β) | 0.06 (-0.04, 0.16) | IDE |
| Liu et al., 2019 | Σm-PFOS | General linear models, (β) | 0.24 (-0.09, 0.57) | IDE |
| Liu et al., 2019 | Linear PFOS | General linear models, (β) | 0.30 (-0.12, 0.71) | IDE |
| Li et al., 2020 | PFNA | Multivariable linear regression (β) | 0.07 (-0.09, 0.23) | IDE |
| Preston et al., 2020 | PFNA | Multinomial logistic regression (OR) | Q1: (ref) | null |
|  |  |  | Q2: -0.9 (-4.2, 2.5) |  |
|  |  |  | Q3: 1.1 (-2.8, 5.1) |  |
|  |  |  | Q4: 0.8 (-2.8, 4.4) |  |
| Li et al., 2020 | PFHxS | Multivariable linear regression (β) | 0.07 (-0.06, 0.21) | IDE |
| Preston et al., 2020 | PFHxS | Multinomial logistic regression (OR) | Q1: (ref) | null |
|  |  |  | Q2: -0.7 (-4.3, 3.0) |  |
|  |  |  | Q3: 1.9 (-1.8, 5.6) |  |
|  |  |  | Q4: 1.2 (-2.6, 5.0) |  |
| Ren et al., 2020 | PFHxS | Logistic regression (OR) | 0.92 (0.45, 1.86) | null |
| Li et al., 2020 | PFDA | Multivariable linear regression (β) | 0.10 (-0.05, 0.25) | IDE |
| Li et al., 2020 | PFUnDA | Multivariable linear regression (β) | 0.05 (-0.08, 0.19) | IDE |
| Li et al., 2020 | PFDoDA | Multivariable linear regression (β) | 0.04 (-0.07, 0.15) | IDE |
| Li et al., 2020 | PFHpA | Multivariable linear regression (β) | 0.07 (-0.13, 0.27) | IDE |
| Li et al., 2020 | PFTrDA | Multivariable linear regression (β) | 0.12 (-0.02, 0.25) | IDE |
| Li et al., 2020 | PFTeDA | Multivariable linear regression (β) | 0.03 (-0.11, 0.17) | IDE |
| Li et al., 2020 | 6:2 Cl-PFESA | Multivariable linear regression (β) | 0.03 (-0.08, 0.14) | IDE |
| Li et al., 2020 | 8:2 Cl-PFESA | Multivariable linear regression (β) | 0.01 (-0.08, 0.11) | IDE |
| Liu et al., 2019 | Long-chain PFCAs | General linear models, (β) | 0.58 (-0.03, 1.18) | IDE |
| Liu et al., 2019 | Short-chain PFSAs | General linear models, (β) | -0.03 (-0.46, 0.39) | IDE |
| Liu et al., 2019 | Long-chain PFSAs | General linear models, (β) | 0.28 (-0.13, 0.69) | IDE |
| Liu et al., 2019 | Total PFSAs | General linear models, (β) | 0.30 (-0.11, 0.7) | IDE |
| Preston et al., 2020 | NEtFOSAA | Multinomial logistic regression (OR) | Q1: (ref) | nonmonotonic |
|  |  |  | Q2: -1.9 (-5.4, 1.7) |  |
|  |  |  | Q3: 1.7 (-2.0, 5.4) |  |
|  |  |  | Q4: 0.4 (-3.4, 4.1) |  |
| **Outcome: 2-hr Glucose test** | | | | |
| Liu et al., 2019 | Linear PFOA | General linear models, (β) | **0.73 (0.27, 1.18)** | **+** |
| Li et al., 2020 | PFNA | Multivariable linear regression (β) | **0.13 (0.01, 0.25)** | **+** |
| Li et al., 2020 | PFBS | Multivariable linear regression (β) | **0.07 (0.01, 0.13)** | **+** |
| Liu et al., 2019 | Short-chain PFCAs | General linear models, (β) | **0.26 (0.03, 0.50)** | **+** |
| Liu et al., 2019 | Long-chain PFCAs | General linear models, (β) | **0.85 (0.35, 1.35)** | **+** |
| Liu et al., 2019 | Total PFCAs | General linear models, (β) | **0.95 (0.43, 1.48)** | **+** |
| Jensen et al., 2018 | PFOA | Linear regression (β, % change) | High GDM risk: -2.6 (-6.9, 1.8) | null |
|  |  |  | Low GDM risk: -0.02 (-4.7, 4.8) | null |
| Li et al., 2020 | PFOA | Multivariable linear regression (β) | 0.10 (-0.04, 0.24) | IDE |
| Liu et al., 2019 | Σm-PFOA | General linear models, (β) | 0.11 (-0.07, 0.30) | IDE |
| Li et al., 2020 | PFOS | Multivariable linear regression (β) | 0.05 (-0.02, 0.13) | IDE |
| Jensen et al., 2018 | PFOS | Linear regression (β, % change) | High GDM risk: 2.9 (-2.8, 8.9) | null |
|  |  |  | Low GDM risk: 2.3 (-3.7, 8.8) | null |
| Liu et al., 2019 | Σm-PFOS | General linear models, (β) | 0.02 (-0.26, 0.31) | IDE |
| Liu et al., 2019 | Linear PFOS | General linear models, (β) | 0.32 (-0.03, 0.67) | IDE |
| Jensen et al., 2018 | PFHxS | Linear regression (β, % change) | High GDM risk: 2.9 (-0.8, 6.8) | null |
|  |  |  | Low GDM risk: 0.2 (-3.4, 3.9) | null |
| Li et al., 2020 | PFHxS | Multivariable linear regression (β) | 0.05 (-0.05, 0.16) | IDE |
| Jensen et al., 2018 | PFNA | Linear regression (β, % change) | High GDM risk: -1.3 (-6.5, 4.2) | null |
|  |  |  | Low GDM risk: 2.4 (-3.0, 8.2) | null |
| Li et al., 2020 | PFDA | Multivariable linear regression (β) | 0.08 (-0.02, 0.17) | IDE |
| Jensen et al., 2018 | PFDA | Linear regression (β, % change) | High GDM risk: -3.3 (-8.7, 2.5) | null |
|  |  |  | Low GDM risk: 2.4 (-3.4, 8.6) | null |
| Li et al., 2020 | PFUnDA | Multivariable linear regression (β) | 0.06 (-0.05, 0.16) | IDE |
| Li et al., 2020 | PFDoDA | Multivariable linear regression (β) | 0.01 (-0.07, 0.09) | IDE |
| Li et al., 2020 | PFTrDA | Multivariable linear regression (β) | 0.07 (-0.03, 0.18) | IDE |
| Li et al., 2020 | PFTeDA | Multivariable linear regression (β) | 0.00 (-0.11, 0.11) | IDE |
| Li et al., 2020 | PFHpA | Multivariable linear regression (β) | 0.01 (-0.14, 0.16) | IDE |
| Li et al., 2020 | 6:2 Cl-PFESA | Multivariable linear regression (β) | 0.06 (-0.02, 0.15) | IDE |
| Li et al., 2020 | 8:2 Cl-PFESA | Multivariable linear regression (β) | 0.00 (-0.08, 0.07) | IDE |
| Liu et al., 2019 | Short-chain PFSAs | General linear models, (β) | 0.10 (-0.26, 0.46) | IDE |
| Liu et al., 2019 | Long-chain PFSAs | General linear models, (β) | 0.24 (-0.11, 0.58) | IDE |
| Liu et al., 2019 | Total PFSAs | General linear models, (β) | 0.21 (-0.13, 0.55) | IDE |
| **Outcome: mean 1-hr, 2-hr glucose test** | | | | |
| Wang et al., 2018 a | PFOA | Linear regression (β) | 0.014 (-0.013, 0.041) | IDE |
| Wang et al., 2018 a | PFOS | Linear regression (β) | 0.006 (-0.015, 0.028) | IDE |
| **Outcome: Fasting insulin** | | | | |
| Jensen et al., 2018 | PFHxS | Linear regression (β, % change) | **High GDM risk: 7.7 (0.1, 15.9)** | **+** |
|  |  |  | Low GDM risk: 2.6 (-6.7, 12.9) | null |
| Jensen et al., 2018 | PFNA | Linear regression (β, % change) | **High GDM risk: 12.1 (0.7, 24.8)** | **+** |
|  |  |  | Low GDM risk: 10.0 (-4.7, 26.9) | + |
| Mehta et al., 2021 | ∑PFAS | Multivariable linear regression, per doubling of exposure (β, % change) | -4.07 (-18.01, 12.24) | null |
| Jensen et al., 2018 | PFOA | Linear regression (β, % change) | High GDM risk: -4.0 (-12.2, 5.0) | null |
|  |  |  | Low GDM risk: 9.4 (-3.3, 23.9) | null |
| Mehta et al., 2021 | PFOA | Multivariable linear regression, per doubling of exposure (β, % change) | -3.41 (-12.09, 6.13) | null |
| Wang et al., 2018 a | PFOA | Linear regression (β) | 0.069 (-0.005, 0.143) | IDE |
| Jensen et al., 2018 | PFOS | Linear regression (β, % change) | High GDM risk: 2.7 (-8.5, 15.2) | null |
|  |  |  | Low GDM risk: 3.6 (-11.7, 21.6) | null |
| Mehta et al., 2021 | PFOS | Multivariable linear regression, per doubling of exposure (β, % change) | -2.06 (-13.80, 11.28) | null |
| Wang et al., 2018 a | PFOS | Linear regression (β) | 0.013 (-0.048, 0.074) | IDE |
| Jensen et al., 2018 | PFDA | Linear regression (β, % change) | High GDM risk: -0.2 (-11.2, 12.1) | null |
|  |  |  | Low GDM risk: 1.5 (-12.9, 18.3) | null |
| Mehta et al., 2021 | PFDA | Multivariable linear regression, per doubling of exposure (β, % change) | -4.74 (-12.83, 4.11) | null |
| Mehta et al., 2021 | PFNA | Multivariable linear regression, per doubling of exposure (β, % change) | -6.05 (-18.32, 8.07) | null |
| Mehta et al., 2021 | PFHxS | Multivariable linear regression, per doubling of exposure (β, % change) | -2.73 (-11.38, 6.75) | null |
| **Outcome: HOMA-IR** | | | | |
| Jensen et al., 2018 | PFHxS | Linear regression (β, % change) | **High GDM risk: 9.5 (1.0, 18.8)** | **+** |
|  |  |  | Low GDM risk: 2.8 (-7.5, 14.3) | null |
| Jensen et al., 2018 | PFOA | Linear regression (β, % change) | High GDM risk: -5.2 (-14.2, 4.7) | null |
|  |  |  | Low GDM risk: 10.8 (-3.4, 27.1) | + |
| Jensen et al., 2018 | PFNA | Linear regression (β, % change) | High GDM risk: 12.2 (-0.5, 26.4) | + |
|  |  |  | Low GDM risk: 11.5 (-4.8, 30.7) |  |
| Mehta et al., 2021 | ∑PFAS | Multivariable linear regression, per doubling of exposure (β, % change) | -7.34 (-20.51, 8.01) | null |
| Mehta et al., 2021 | PFOA | Multivariable linear regression, per doubling of exposure (β, % change) | -3.41 (-11.81, 5.80) | null |
| Wang et al., 2018 a | PFOA | Linear regression (β) | 0.074 (-0.011, 0.158) | IDE |
| Jensen et al., 2018 | PFOS | Linear regression (β, % change) | High GDM risk: 2.6 (-9.7, 16.6) | null |
|  |  |  | Low GDM risk: 3.9 (-13.0, 24.0) | null |
| Mehta et al., 2021 | PFOS | Multivariable linear regression, per doubling of exposure (β, % change) | -4.74 (-15.94, 7.96) | null |
| Wang et al., 2018 a | PFOS | Linear regression (β) | 0.005 (-0.064, 0.073) | IDE |
| Jensen et al., 2018 | PFDA | Linear regression (β, % change) | High GDM risk: -1.5 (-13.5, 12.1) | null |
|  |  |  | Low GDM risk: 2.0 (-13.9, 20.9) | null |
| Mehta et al., 2021 | PFDA | Multivariable linear regression, per doubling of exposure (β, % change) | -4.74 (-12.56, 3.79) | null |
| Mehta et al., 2021 | PFNA | Multivariable linear regression, per doubling of exposure (β, % change) | -7.34 (-19.07, 6.09) | null |
| Mehta et al., 2021 | PFHxS | Multivariable linear regression, per doubling of exposure (β, % change) | -4.74 (-12.95, 4.25) | null |
| **Outcome: HOMA-%β** | | | | |
| Jensen et al., 2018 | PFNA | Linear regression (β, % change) | **High GDM risk: 12.4 (2.2, 23.7)** | **+** |
|  |  |  | Low GDM risk: 4.4 (-6.8, 17.0) | null |
| Jensen et al., 2018 | PFOA | Linear regression (β, % change) | High GDM risk: -0.4 (-8.0, 8.0) | null |
|  |  |  | Low GDM risk: 4.9 (-4.9, 15.7) |  |
| Jensen et al., 2018 | PFOS | Linear regression (β, % change) | High GDM risk: 2.9 (-7.1, 14.1) | null |
|  |  |  | Low GDM risk: 2.2 (-10.0, 15.9) |  |
| Jensen et al., 2018 | PFHxS | Linear regression (β, % change) | High GDM risk: 2.3 (-4.3, 9.4) | null |
|  |  |  | Low GDM risk: 1.9 (-5.5, 9.8) |  |
| Jensen et al., 2018 | PFDA | Linear regression (β, % change) | High GDM risk: 3.9 (-6.4, 15.2) | null |
|  |  |  | Low GDM risk: -1.0 (-12.3, 11.7) |  |
| **Outcome: Insulin sensitivity test derived from OGTT, Matsuda Index** | | | | |
| Jensen et al., 2018 | PFNA | Linear regression (β, % change) | High GDM risk: -5.4 (-16.7, 7.5) | null |
|  |  |  | Low GDM risk: -11.6 (-22.9, 1.2) | - |
| Jensen et al., 2018 | PFOA | Linear regression (β, % change) | High GDM risk: 6.1 (-4.5, 17.8) | null |
|  |  |  | Low GDM risk: -8.0 (-18.3, 3.5) | null |
| Jensen et al., 2018 | PFOS | Linear regression (β, % change) | High GDM risk: -1.9 (-14.3, 12.3) | null |
|  |  |  | Low GDM risk: -8.2 (-21.2, 6.9) | null |
| Jensen et al., 2018 | PFHxS | Linear regression (β, % change) | High GDM risk: -7.1 (-14.8, 1.3) | null |
|  |  |  | Low GDM risk: 0.1 (-8.6, 9.6) | null |
| Jensen et al., 2018 | PFDA | Linear regression (β, % change) | High GDM risk: 5.6 (-7.9, 21.1) | null |
|  |  |  | Low GDM risk: -8.7 (-21.1, 5.6) | null |
| **Outcome: Fasting C-peptide** | | | | |
| Jensen et al., 2018 | PFOA | Linear regression (β, % change) | High GDM risk: -1.5 (-6.9, 4.2) | null |
|  |  |  | Low GDM risk: 6.4 (-2.2, 15.9) | null |
| Jensen et al., 2018 | PFOS | Linear regression (β, % change) | High GDM risk: 0.6 (-6.4, 8.2) | null |
|  |  |  | Low GDM risk: 2.9 (-7.8, 15.0) | null |
| Jensen et al., 2018 | PFHxS | Linear regression (β, % change) | High GDM risk: 4.6 (-0.1, 9.6) | null |
|  |  |  | Low GDM risk: 3.0 (-3.5, 9.9) | null |
| Jensen et al., 2018 | PFNA | Linear regression (β, % change) | High GDM risk: 6.4 (-0.6, 13.9) | null |
|  |  |  | Low GDM risk: 6.1 (-3.8, 17.0) | null |
| Jensen et al., 2018 | PFDA | Linear regression (β, % change) | High GDM risk: -2.0 (-9.0, 5.4) | null |
|  |  |  | Low GDM risk: -1.0 (-10.8, 10.0) | null |

**Abbreviations:** β, beta coefficient; HOMA-IR, homeostatic model of assessment of insulin resistance; IDE, indeterminate, IH, isolated hyperglycemia; IGT, impaired glucose tolerance; OGTT, oral glucose tolerance test; OR, odds ratio; Q, quartile; ref, reference; T, tertile

**Bold**: statistically significant

**SUPPLEMENTAL TABLE 5: Evidence of PFAS association with free and** **total triiodothyronine (T3) in pregnancy**

| **Author** | **Exposure** | **Outcome** | **Statistical test (measure of association)** | **Association Value (95% CI or p-value)#** | **Direction** |
| --- | --- | --- | --- | --- | --- |
|  |  |  |  |  |  |
| **Outcome: T3** | | | | | |
| Wang et al., 2014 | PFDA | T3 | Linear regression (β) | **0.002 (0.000, 0.003)** | **+** |
| Yang et al., 2016 | 6:2 FTS | T3 | Spearman correlation (rho) | **0.205 (P <0.05)** | **+ (weak)** |
| Yang et al., 2016 | PFOA | T3 | Spearman correlation (rho) | 0.102 (P >0.05) | + (weak) |
| Yang et al., 2016 | PFOS | T3 | Spearman correlation (rho) | 0.008 (P >0.05) | + (weak) |
| Yang et al., 2016 | PFHxS | T3 | Spearman correlation (rho) | 0.084 (P >0.05) | + (weak) |
| Berg et al., 2015 | PFDA | T3 | Mixed-effects linear model (β) | Q1: ref | **-** |
|  |  |  |  | Q2: -0.04 (-0.08, 0.04) |  |
|  |  |  |  | Q3: -0.05 (-0.08, 0.00) |  |
|  |  |  |  | **Q4: -0.1 (-0.14,-0.06)** |  |
| Yang et al., 2016 | PFDoDA | T3 | Spearman correlation (rho) | **-0.301 (P <0.01)** | **- (weak)** |
| Yang et al., 2016 | PFNA | T3 | Spearman correlation (rho) | -0.018 (P >0.05) | - (weak) |
| Yang et al., 2016 | PFDA | T3 | Spearman correlation (rho) | -0.079 (P >0.05) | - (weak) |
| Yang et al., 2016 | PFUnDA | T3 | Spearman correlation (rho) | -0.097 (P >0.05) | - (weak) |
| Yang et al., 2016 | NMeFOSAA | T3 | Spearman correlation (rho) | -0.028 (P >0.05) | - (weak) |
| Lebeaux et al., 2019 | PFOA | T3 | Multivariable linear regression (β) | -0.01 (-0.05, 0.04) | IDE |
| Wang et al., 2014 | PFOA | T3 | Linear regression (β) | -0.000 (-0.002, 0.009) | IDE |
| Lebeaux et al., 2019 | PFOS | T3 | Multivariable linear regression (β) | -0.02 (-0.07, 0.03) | IDE |
| Wang et al., 2014 | PFOS | T3 | Linear regression (β) | 0.000 (-0.002, 0.001) | IDE |
| Lebeaux et al., 2019 | PFNA | T3 | Multivariable linear regression (β) | -0.03 (-0.09, 0.03) | IDE |
| Wang et al., 2014 | PFNA | T3 | Linear regression (β) | -0.001 (-0.003, 0.002) | IDE |
| Lebeaux et al., 2019 | PFHxS | T3 | Multivariable linear regression (β) | -0.01 (-0.04, 0.02) | IDE |
| Wang et al., 2014 | PFHxS | T3 | Linear regression (β) | -0.002 (-0.005, 0.001) | IDE |
| Wang et al., 2014 | PFUnDA | T3 | Linear regression (β) | 0.000 (-0.001, 0.000) | IDE |
| Wang et al., 2014 | PFDoDA | T3 | Linear regression (β) | -0.005 (-0.022, 0.011) | IDE |
| **Outcome: FT3** | | | | | |
| Aimuzi et al., 2020 | PFNA | FT3 | Linear regression (β) | **0.179 (0.047, 0.311)** | **+** |
| Aimuzi et al., 2020 | PFHxS | FT3 | Linear regression (β) | **0.197 (0.054, 0.339)** | **+** |
| Yang et al., 2016 | 6:2 FTS | FT3 | Spearman correlation (rho) | **0.16 (P <0.05)** | **+ (weak)** |
| Yang et al., 2016 | NMeFOSAA | FT3 | Spearman correlation (rho) | **0.163 (P <0.05)** | **+ (weak)** |
| Yang et al., 2016 | PFOA | FT3 | Spearman correlation (rho) | 0.024 (P >0.05) | + (weak) |
| Yang et al., 2016 | PFOS | FT3 | Spearman correlation (rho) | 0.025 (P >0.05) | + (weak) |
| Yang et al., 2016 | PFHxS | FT3 | Spearman correlation (rho) | 0.124 (P >0.05) | + (weak) |
| Berg et al., 2015 | PFUnDA | FT3 | Mixed-effects linear model (β) | Q1: ref | **negative** |
|  |  |  |  | Q2: -0.08 (-0.15, 0.00) |  |
|  |  |  |  | Q3: -0.09 (-0.16, -0.01) |  |
|  |  |  |  | **Q4: -0.18 (-0.25,-0.12)** |  |
| Yang et al., 2016 | PFDoDA | FT3 | Spearman correlation (rho) | **-0.268 (P <0.01)** | **- (weak)** |
| Yang et al., 2016 | PFNA | FT3 | Spearman correlation (rho) | -0.063 (P >0.05) | - (weak) |
| Yang et al., 2016 | PFDA | FT3 | Spearman correlation (rho) | -0.087 (P >0.05) | - (weak) |
| Yang et al., 2016 | PFUnDA | FT3 | Spearman correlation (rho) | -0.121 (P >0.05) | - (weak) |
| Aimuzi et al., 2020 | PFOA | FT3 | Linear regression (β) | 0.105 (-0.046, 0.256) | IDE |
| Lebeaux et al., 2019 | PFOA | FT3 | Multivariable linear regression (β) | -0.01 (-0.04, 0.01) | IDE |
| Xiao et al., 2020 | PFOA | FT3 | Multiple linear regression (exp(β) - 1) × 100%) | 3.1 (-1.2, 7.6) | null |
| Reardon et al., 2019 | Linear-PFOA | FT3 | Multivariate mixed effect model (β) | 0.001 (P=0.535) | IDE |
| Aimuzi et al., 2020 | PFOS | FT3 | Linear regression (β) | 0.112 (-0.004, 0.227) | IDE |
| Lebeaux et al., 2019 | PFOS | FT3 | Multivariable linear regression (β) | -0.03 (-0.06, 0.00) | IDE |
| Xiao et al., 2020 | PFOS | FT3 | Multiple linear regression (exp(β) - 1) × 100%) | 2.2 (-3.6, 8.5) | null |
| Reardon et al., 2019 | Total-PFOS | FT3 | Multivariate mixed effect model (β) | -0.0028 (P=0.242) | IDE |
| Reardon et al., 2019 | Linear-PFOS | FT3 | Multivariate mixed effect model (β) | -0.004 (P=0.248) | IDE |
| Reardon et al., 2019 | 1m-PFOS | FT3 | Multivariate mixed effect model (β) | -0.122 (P=0.188) | IDE |
| Reardon et al., 2019 | ∑Br-PFOS | FT3 | Multivariate mixed effect model (β) | -0.008 (P=0.284) | IDE |
| Reardon et al., 2019 | ∑3m+4m-PFOS | FT3 | Multivariate mixed effect model (β) | -0.036 (P=0.290) | IDE |
| Reardon et al., 2019 | 5m-PFOS | FT3 | Multivariate mixed effect model (β) | -0.028 (P=0.182) | IDE |
| Reardon et al., 2019 | *iso-PFOS* | FT3 | Multivariate mixed effect model (β) | -0.011 (P=0.533) | IDE |
| Lebeaux et al., 2019 | PFNA | FT3 | Multivariable linear regression (β) | -0.02 (-0.05, 0.02) | IDE |
| Reardon et al., 2019 | PFNA | FT3 | Multivariate mixed effect model (β) | 0.004 (P=0.330) | IDE |
| Xiao et al., 2020 | PFNA | FT3 | Multiple linear regression (exp(β) - 1) × 100%) | -2.7 (-8.5, 3.6) | null |
| Aimuzi et al., 2020 | PFDA | FT3 | Linear regression (β) | 0.107 (0, 0.214) | IDE |
| Reardon et al., 2019 | PFDA | FT3 | Multivariate mixed effect model (β) | 0.003 (P=0.736) | IDE |
| Xiao et al., 2020 | PFDA | FT3 | Multiple linear regression (exp(β) - 1) × 100%) | -1.4 (-6.9, 4.5) | null |
| Lebeaux et al., 2019 | PFHxS | FT3 | Multivariable linear regression (β) | -0.02 (-0.04, 0.00) | IDE |
| Reardon et al., 2019 | PFHxS | FT3 | Multivariate mixed effect model (β) | 0.0002 (P=0.955) | IDE |
| Xiao et al., 2020 | PFHxS | FT3 | Multiple linear regression (exp(β) - 1) × 100%) | -0.7 (-5.6, 4.5) | null |
| Aimuzi et al., 2020 | PFUnDA | FT3 | Linear regression (β) | 0.062 (-0.05, 0.175) | IDE |
| Xiao et al., 2020 | PFUnDA | FT3 | Multiple linear regression (exp(β) - 1) × 100%) | -1.8 (-5.8, 2.4) | null |
| Reardon et al., 2019 | PFUnDA | FT3 | Multivariate mixed effect model (β) | 0.014 (P=0.698) | IDE |
| Aimuzi et al., 2020 | PFDoDA | FT3 | Linear regression (β) | 0.005 (-0.166, 0.175) | IDE |
| Xiao et al., 2020 | PFDoDA | FT3 | Multiple linear regression (exp(β) - 1) × 100%) | 0.6 (-1.3, 2.4) | null |
| Xiao et al., 2020 | NMeFOSAA | FT3 | Multiple linear regression (exp(β) - 1) × 100%) | 0.1 (-2.6, 2.8) | null |
| Xiao et al., 2020 | NEtFOSAA | FT3 | Multiple linear regression (exp(β) - 1) × 100%) | 1.6 (-1.8, 5.0) | null |
| Xiao et al., 2020 | PFOSA | FT3 | Multiple linear regression (exp(β) - 1) × 100%) | -0.7 (-1.9, 0.6) | null |
| Aimuzi et al., 2020 | PFBS | FT3 | Linear regression (β) | -0.033 (-0.117, 0.051) | IDE |
| Aimuzi et al., 2020 | PFHpA | FT3 | Linear regression (β) | -0.013 (-0.081, 0.054) | IDE |
| Xiao et al., 2020 | PFHpA | FT3 | Multiple linear regression (exp(β) - 1) × 100%) | 1.0 (-0.9, 2.9) | null |
| Xiao et al., 2020 | PFHpS | FT3 | Multiple linear regression (exp(β) - 1) × 100%) | 0.7 (-0.7, 2.2) | null |

**Abbreviations:** β, beta coefficient; fT3, free triiodothyronine; IDE, indeterminate; Q, quartile; r, rho; T3, triiodothyronine

**Bold**: statistically significant

# Tables present associations based on THs measured in maternal plasma or serum

**SUPPLEMENTAL TABLE 6: Evidence of PFAS association with free and total thyroxine (T4) in pregnancy**

| **Author** | **Exposure** | **Outcome** | **Statistical test (measure of association)** | **Association Value (95% CI or p-value)#** | **Direction** |
| --- | --- | --- | --- | --- | --- |
|  |  |  |  |  |  |
| **Outcome: T4** | | | | | |
| Yang et al., 2016 | 6:2 FTS | T4 | Spearman correlation (rho) | **0.172 (P <0.05)** | **+ (weak)** |
| Yang et al., 2016 | PFOA | T4 | Spearman correlation (rho) | 0.062 (P >0.05) | + (weak) |
| Yang et al., 2016 | PFOS | T4 | Spearman correlation (rho) | 0.021 (P >0.05) | + (weak) |
| Yang et al., 2016 | PFHxS | T4 | Spearman correlation (rho) | 0.084 (P >0.05) | + (weak) |
| Yang et al., 2016 | PFUnDA | T4 | Spearman correlation (rho) | 0.03 (P >0.05) | + (weak) |
| Yang et al., 2016 | NMeFOSAA | T4 | Spearman correlation (rho) | 0.072 (P >0.05) | + (weak) |
| Wang et al., 2014 | PFNA | T4 | Linear regression (β) | **-0.189 (-0.333, -0.046)** | **-** |
| Wang et al., 2014 | PFUnDA | T4 | Linear regression (β) | **-0.062 (-0.097, -0.026)** | **-** |
| Yang et al., 2016 | PFDoDA | T4 | Spearman correlation (rho) | **-0.160 (P <0.05)** | **- (weak)** |
| Wang et al., 2014 | PFDoDA | T4 | Linear regression (β) | **-1.742 (-2.785, -0.700)** | **-** |
| Yang et al., 2016 | PFNA | T4 | Spearman correlation (rho) | -0.006 (P >0.05) | - (weak) |
| Yang et al., 2016 | PFDA | T4 | Spearman correlation (rho) | -0.079 (P >0.05) | - (weak) |
| Lebeaux et al., 2019 | PFOA | T4 | Multivariable linear regression (β) | -0.03 (-0.10, 0.04) | IDE |
| Preston et al., 2018 | PFOA | T4 | Multivariable linear regression (β) | 0.09 (-0.08, 0.27) | IDE |
| Wang et al., 2014 | PFOA | T4 | Linear regression (β) | 0.011 (-0.108, 0.130) | IDE |
| Xiao et al., 2020 | PFOA | T4 | Multiple linear regression (exp(β) - 1) × 100%) | 0.7 (-5.5, 7.3) | null |
| Lebeaux et al., 2019 | PFOS | T4 | Multivariable linear regression (β) | 0.02 (-0.08, 0.08) | IDE |
| Preston et al., 2018 | PFOS | T4 | Multivariable linear regression (β) | 0.01 (-0.14, 0.16) | IDE |
| Wang et al., 2014 | PFOS | T4 | Linear regression (β) | 0.019 (-0.016, 0.053) | IDE |
| Xiao et al., 2020 | PFOS | T4 | Multiple linear regression (exp(β) - 1) × 100%) | 0.1 (-8.3, 9.3) | null |
| Lebeaux et al., 2019 | PFNA | T4 | Multivariable linear regression (β) | -0.07 (-0.17, 0.03) | IDE |
| Preston et al., 2018 | PFNA | T4 | Multivariable linear regression (β) | -0.05 (-0.16, 0.05) | IDE |
| Xiao et al., 2020 | PFNA | T4 | Multiple linear regression (exp(β) - 1) × 100%) | -1.2 (-9.9, 8.2) | null |
| Xiao et al., 2020 | PFDA | T4 | Multiple linear regression (exp(β) - 1) × 100%) | -2.1 (-10.1, 6.7) | null |
| Lebeaux et al., 2019 | PFHxS | T4 | Multivariable linear regression (β) | 0.00 (-0.05, 0.06) | IDE |
| Preston et al., 2018 | PFHxS | T4 | Multivariable linear regression (β) | -0.05 (-0.14, 0.04) | IDE |
| Wang et al., 2014 | PFHxS | T4 | Linear regression (β) | -0.130 (-0.316, 0.057) | IDE |
| Xiao et al., 2020 | PFHxS | T4 | Multiple linear regression (exp(β) - 1) × 100%) | 0.4 (-6.9, 8.2) | null |
| Wang et al., 2014 | PFDA | T4 | Linear regression (β) | 0.047 (-0.028, 0.123) | IDE |
| Xiao et al., 2020 | PFUnDA | T4 | Multiple linear regression (exp(β) - 1) × 100%) | 0.4 (-5.6, 6.9) | null |
| Xiao et al., 2020 | PFDoDA | T4 | Multiple linear regression (exp(β) - 1) × 100%) | 0.1 (-2.6, 2.9) | null |
| Preston et al., 2018 | NMeFOSAA | T4 | Multivariable linear regression (β) | 0.03 (-0.10, 0.15) | IDE |
| Xiao et al., 2020 | NMeFOSAA | T4 | Multiple linear regression (exp(β) - 1) × 100%) | -2.6 (-6.3, 1.4) | null |
| Preston et al., 2018 | NEtFOSAA | T4 | Multivariable linear regression (β) | -0.01 (-0.09, 0.06) | null |
| Xiao et al., 2020 | NEtFOSAA | T4 | Multiple linear regression (exp(β) - 1) × 100%) | 1.6 (-3.2, 6.8) | null |
| Xiao et al., 2020 | PFOSA | T4 | Multiple linear regression (exp(β) - 1) × 100%) | 2.5 (0.7, 4.3) | null |
| Xiao et al., 2020 | PFHpA | T4 | Multiple linear regression (exp(β) - 1) × 100%) | 0.8 (-2.0, 3.7) | null |
| Xiao et al., 2020 | PFHpS | T4 | Multiple linear regression (exp(β) - 1) × 100%) | 0.9 (-1.3, 3.0) | null |
| **Outcome: FT4** | | | | | |
| Aimuzi et al., 2020 | PFOA | FT4 | Linear regression (β) | **0.121 (0.015, 0.227)** | **+** |
| Aimuzi et al., 2020 | PFHxS | FT4 | Linear regression (β) | **0.123 (0.024, 0.222)** | **+** |
| Yang et al., 2016 | 6:2 FTS | FT4 | Spearman correlation (rho) | **0.164 (P <0.05)** | **+ (weak)** |
| Chan et al., 2011 | PFHxS | hypothyroxinemia (normal TSH, lowest 10th percentile FT4) | Conditional logistic regression (OR) | 1.12 (0.89, 1.41) | + |
| Yang et al., 2016 | PFHxS | FT4 | Spearman correlation (rho) | 0.038 (P >0.05) | + (weak) |
| Yang et al., 2016 | NMeFOSAA | FT4 | Spearman correlation (rho) | 0.081 (P >0.05) | + (weak) |
| Wang et al., 2014 | PFNA | FT4 | Linear regression (β) | **-0.019 (-0.028, -0.009)** | **-** |
| Preston et al., 2018 | PFOA | FT4 (index) | Multivariable linear regression (exp(β) - 1) × 100%) | **-1.87 (-3.40, -0.31)** | **-** |
| Reardon et al., 2019 | PFHxS | FT4 | Multivariate mixed effect model (β) | **-0.006 (P=0.034)** | **-** |
| Wang et al., 2014 | PFUnDA | FT4 | Linear regression (β) | **-0.004 (-0.007, -0.002)** | **-** |
| Yang et al., 2016 | PFDoDA | FT4 | Spearman correlation (rho) | **-0.160 (P <0.05)** | **- (weak)** |
| Wang et al., 2014 | PFDoDA | FT4 | Linear regression (β) | **-0.132 (-0.204, -0.059)** | **-** |
| Chan et al., 2011 | PFOS | hypothyroxinemia (normal TSH, lowest 10th percentile FT4) | Conditional logistic regression (OR) | 0.88 (0.63, 1.24) | - |
| Yang et al., 2016 | PFOS | FT4 | Spearman correlation (rho) | -0.057 (P >0.05) | - (weak) |
| Yang et al., 2016 | PFNA | FT4 | Spearman correlation (rho) | -0.072 (P >0.05) | - (weak) |
| Yang et al., 2016 | PFDA | FT4 | Spearman correlation (rho) | -0.086 (P >0.05) | - (weak) |
| Yang et al., 2016 | PFUnDA | FT4 | Spearman correlation (rho) | -0.062 (P >0.05) | - (weak) |
| Aimuzi et al., 2020 | PFDoDA | FT4 | Linear regression (β) | -0.073 (-0.177, 0.03) | - (weak) |
| Chan et al., 2011 | PFOA | hypothyroxinemia (normal TSH, lowest 10th percentile FT4) | Conditional logistic regression (OR) | 0.94 (0.74, 1.18) | null |
| Inoue et al., 2019 | PFOA | FT4 | Multivariate linear regression (exp(β) - 1) × 100%) | 0.6 (-0.6, 1.8) | null |
| Lebeaux et al., 2019 | PFOA | FT4 | Multivariable linear regression (β) | -0.01 (-0.06, 0.03) | IDE |
| Wang et al., 2014 | PFOA | FT4 | Linear regression (β) | -0.003 (-0.012, 0.005) | IDE |
| Webster et al., 2014 | PFOA | FT4 | Mixed-effects linear model (β) | -0.06 (-0.3, 0.2) | IDE |
| Xiao et al., 2020 | PFOA | FT4 | Multiple linear regression (exp(β) - 1) × 100%) | -0.4 (-5.4, 4.8) | null |
| Yang et al., 2016 | PFOA | FT4 | Spearman correlation (rho) | 0.00 (P >0.05) | null |
| Reardon et al., 2019 | Linear PFOA | FT4 | Multivariate mixed effect model (β) | -0.002 (P=0.138) | IDE |
| Aimuzi et al., 2020 | PFOS | FT4 | Linear regression (β) | 0.046 (-0.035, 0.127) | IDE |
| Inoue et al., 2019 | PFOS | FT4 | Multivariate linear regression (exp(β) - 1) × 100%) | 0.3 (-1.1, 1.7) | null |
| Lebeaux et al., 2019 | PFOS | FT4 | Multivariable linear regression (β) | 0.02 (-0.02, 0.07) | IDE |
| Preston et al., 2018 | PFOS | FT4 (index) | Multivariable linear regression (exp(β) - 1) × 100%) | -1.04 (-2.36, 0.29) | null |
| Reardon et al., 2019 | PFOS | FT4 | Multivariate mixed effect model (β) | -0.00000482 (P=0.999) | IDE |
| Wang et al., 2014 | PFOS | FT4 | Linear regression (β) | 0.001 (-0.002, 0.003) | IDE |
| Webster et al., 2014 | PFOS | FT4 | Mixed-effects linear model (β) | 0.03 (-0.2, 0.2) | IDE |
| Xiao et al., 2020 | PFOS | FT4 | Multiple linear regression (exp(β) - 1) × 100%) | -2.9 (-9.4, 4.3) | null |
| Reardon et al., 2019 | Linear-PFOS | FT4 | Multivariate mixed effect model (β) | 0.002 (P=0.656) | IDE |
| Reardon et al., 2019 | 5m-PFOS | FT4 | Multivariate mixed effect model (β) | -0.012 (P=0.565) | IDE |
| Reardon et al., 2019 | *iso-PFOS* | FT4 | Multivariate mixed effect model (β) | 0.001 (P=0.965) | IDE |
| Reardon et al., 2019 | ∑Br-PFOS | FT4 | Multivariate mixed effect model (β) | -0.002 (P=0.810) | IDE |
| Reardon et al., 2019 | 1m-PFOS | FT4 | Multivariate mixed effect model (β) | -0.116 (P=0.198) | IDE |
| Reardon et al., 2019 | ∑3m+4m-PFOS | FT4 | Multivariate mixed effect model (β) | 0.019 (P=0.59) | IDE |
| Aimuzi et al., 2020 | PFNA | FT4 | Linear regression (β) | 0.053 (-0.037, 0.144) | IDE |
| Inoue et al., 2019 | PFNA | FT4 | Multivariate linear regression (exp(β) - 1) × 100%) | -0.1 (-1.2, 0.9) | null |
| Lebeaux et al., 2019 | PFNA | FT4 | Multivariable linear regression (β) | -0.01 (-0.07, 0.05) | IDE |
| Preston et al., 2018 | PFNA | FT4 (index) | Multivariable linear regression (exp(β) - 1) × 100%) | -0.57 (-1.52, 0.40) | null |
| Reardon et al., 2019 | PFNA | FT4 | Multivariate mixed effect model (β) | -0.004 (P=0.379) | null |
| Webster et al., 2014 | PFNA | FT4 | Mixed-effects linear model (β) | -0.03 (-0.2, 0.2) | IDE |
| Xiao et al., 2020 | PFNA | FT4 | Multiple linear regression (exp(β) - 1) × 100%) | -5.7 (-12.4, 1.4) | null |
| Aimuzi et al., 2020 | PFDA | FT4 | Linear regression (β) | 0.05 (-0.026, 0.127) | IDE |
| Inoue et al., 2019 | PFDA | FT4 | Multivariate linear regression (exp(β) - 1) × 100%) | 0.8 (-0.4, 1.9) | null |
| Reardon et al., 2019 | PFDA | FT4 | Multivariate mixed effect model (β) | -0.010 (P=0.314) | IDE |
| Xiao et al., 2020 | PFDA | FT4 | Multiple linear regression (exp(β) - 1) × 100%) | -1.5 (-8.1, 5.5) | null |
| Inoue et al., 2019 | PFHxS | FT4 | Multivariate linear regression (exp(β) - 1) × 100%) | -0.3 (-1.6, 0.9) | null |
| Lebeaux et al., 2019 | PFHxS | FT4 | Multivariable linear regression (β) | 0.02 (-0.01, 0.05) | IDE |
| Preston et al., 2018 | PFHxS | FT4 (index) | Multivariable linear regression (exp(β) - 1) × 100%) | -0.60 (-1.39, 0.19) | null |
| Wang et al., 2014 | PFHxS | FT4 | Linear regression (β) | -0.010 (-0.023, 0.003) | IDE |
| Webster et al., 2014 | PFHxS | FT4 | Mixed-effects linear model (β) | -0.02 (-0.1, 0.07) | IDE |
| Xiao et al., 2020 | PFHxS | FT4 | Multiple linear regression (exp(β) - 1) × 100%) | 5.9 (-0.2, 12.4) | null |
| Wang et al., 2014 | PFDA | FT4 | Linear regression (β) | -0.001 (-0.006, 0.005) | IDE |
| Aimuzi et al., 2020 | PFUnDA | FT4 | Linear regression (β) | 0.033 (-0.048, 0.114) | IDE |
| Xiao et al., 2020 | PFUnDA | FT4 | Multiple linear regression (exp(β) - 1) × 100%) | -0.1 (-5.0, 5.0) | null |
| Reardon et al., 2019 | PFUnDA | FT4 | Multivariate mixed effect model (β) | -0.037 (P=0.321) | IDE |
| Xiao et al., 2020 | PFDoDA | FT4 | Multiple linear regression (exp(β) - 1) × 100%) | 0.2 (-2.0, 2.4) | null |
| Preston et al., 2018 | NMeFOSAA | FT4 (index) | Multivariable linear regression (exp(β) - 1) × 100%) | -0.82 (-1.93, 0.29) | null |
| Xiao et al., 2020 | NMeFOSAA | FT4 | Multiple linear regression (exp(β) - 1) × 100%) | -2.5 (-5.5, 0.7) | null |
| Preston et al., 2018 | NEtFOSAA | FT4 (index) | Multivariable linear regression (exp(β) - 1) × 100%) | 0.07 (-0.61, 0.77) | null |
| Xiao et al., 2020 | NEtFOSAA | FT4 | Multiple linear regression (exp(β) - 1) × 100%) | 0.2 (-3.7, 4.3) | null |
| Xiao et al., 2020 | PFOSA | FT4 | Multiple linear regression (exp(β) - 1) × 100%) | -0.8 (-2.3, 0.6) | null |
| Aimuzi et al., 2020 | PFBS | FT4 | Linear regression (β) | 0.016 (-0.048, 0.08) | IDE |
| Aimuzi et al., 2020 | PFHpA | FT4 | Linear regression (β) | -0.035 (-0.093, 0.024) | IDE |
| Xiao et al., 2020 | PFHpA | FT4 | Multiple linear regression (exp(β) - 1) × 100%) | 0.5 (-1.8, 2.7) | null |
| Inoue et al., 2019 | PFHpS | FT4 | Multivariate linear regression (exp(β) - 1) × 100%) | 0.1 (-1.3, 1.5) | null |
| Xiao et al., 2020 | PFHpS | FT4 | Multiple linear regression (exp(β) - 1) × 100%) | -0.8 (-2.5, 0.9) | null |

**Abbreviations:** β, beta coefficient; fT4, free thyroxine; IDE, indeterminate; r, rho, T4, thyroxine

**Bold**: statistically significant

# Tables present associations based on THs measured in maternal plasma or serum

**SUPPLEMENTAL TABLE 7: Evidence of PFAS association with thyrotropin (TSH) in pregnancy**

| **Author** | **Exposure** | **Outcome** | **Statistical test (measure of association)** | **Association Value (95% CI or p-value)#** | **Direction** |
| --- | --- | --- | --- | --- | --- |
|  |  |  |  |  |  |
| **Outcome: TSH** | | | | |  |
| Berg et al., 2015 | PFOS | TSH | Mixed-effects linear model (β) | Q1: ref | **+** |
|  |  |  |  | Q2: 0.18 (0.06, 0.31) |  |
|  |  |  |  | **Q3: 0.26 (0.13, 0.40)** |  |
|  |  |  |  | **Q4: 0.35 (0.21, 0.50)** |  |
| Wang et al., 2013 | PFOS | TSH | Linear regression (β) | **0.008 (0.001, 0.016)** | **+** |
| Webster et al., 2014 | PFNA | TSH | Mixed-effects linear model (β) | **0.2 (0.01, 0.3)** | **+** |
| Reardon et al., 2019 | ∑Br-PFOS | TSH | Multivariate mixed effect model (β) | **0.286 (P=0.038)** | **+** |
| Reardon et al., 2019 | 5m-PFOS | TSH | Multivariate mixed effect model (β) | **0.851 (P=0.028)** | **+** |
| Reardon et al., 2019 | PFHxS | TSH | Multivariate mixed effect model (β) | **0.144 (P=0.008)** | **+** |
| Wang et al., 2014 | PFHxS | TSH | Linear regression (β) | **0.105 (0.002, 0.207)** | **+** |
| Xiao et al., 2020 | PFOA | TSH | Multiple linear regression (exp(β) - 1) × 100%) | 12.6 (-4.5, 32.8) | + |
| Xiao et al., 2020 | PFOS | TSH | Multiple linear regression (exp(β) - 1) × 100%) | 16.4 (-7.5, 46.5) | + |
| Xiao et al., 2020 | PFDA | TSH | Multiple linear regression (exp(β) - 1) × 100%) | 13.9 (-9.0, 42.6) | + |
| Xiao et al., 2020 | PFNA | TSH | Multiple linear regression (exp(β) - 1) × 100%) | 20.1 (-5.3, 52.3) | + |
| Inoue et al., 2019 | PFHxS | TSH | Multivariate linear regression (exp(β) - 1) × 100%) | 2.3 (-3.6, 8.8) | + |
| Yang et al., 2016 | PFOS | TSH | Spearman correlation (rho) | **-0.261 (P <0.01)** | **- (weak)** |
| Yang et al., 2016 | PFDA | TSH | Spearman correlation (rho) | **-0.216 (P <0.01)** | **- (weak)** |
| Yang et al., 2016 | PFNA | TSH | Spearman correlation (rho) | **-0.170 (P <0.05)** | **- (weak)** |
| Aimuzi et al., 2020 | PFHxS | TSH | Linear regression (β) | **-0.115 (-0.216, -0.014)** | **-** |
| Yang et al., 2016 | PFUnDA | TSH | Spearman correlation (rho) | **-0.202 (P <0.05)** | **- (weak)** |
| Yang et al., 2016 | PFDoDA | TSH | Spearman correlation (rho) | **-0.231 (P <0.01)** | **- (weak)** |
| Yang et al., 2016 | PFOA | TSH | Spearman correlation (rho) | -0.124 (P >0.05) | - (weak) |
| Yang et al., 2016 | PFHxS | TSH | Spearman correlation (rho) | -0.154 (P >0.05) | - (weak) |
| Yang et al., 2016 | 6:2 FTS | TSH | Spearman correlation (rho) | -0.013 (P >0.05) | - (weak) |
| Yang et al., 2016 | NMeFOSAA | TSH | Spearman correlation (rho) | -0.046 (P >0.05) | - (weak) |
| Aimuzi et al., 2020 | PFOA | TSH | Linear regression (β) | -0.03 (-0.138, 0.078) | IDE |
| Inoue et al., 2019 | PFOA | TSH | Multivariate linear regression (exp(β) - 1) × 100%) | 0.3 (-6.9, 8.1) | null |
| Lebeaux et al., 2019 | PFOA | TSH | Multivariable linear regression (β) | 0.09 (-0.14, 0.33) | IDE |
| Preston et al., 2018 | PFOA | TSH | Multivariable linear regression (exp(β) - 1) × 100%) | 0.28 (-9.26, 10.8) | null |
| Wang et al., 2013 | PFOA | TSH | Linear regression (β) | -0.0001 (-0.045, 0.044) | IDE |
| Wang et al., 2014 | PFOA | TSH | Linear regression (β) | 0.011 (-0.057, 0.078) | IDE |
| Webster et al., 2014 | PFOA | TSH | Mixed-effects linear model (β) | 0.1 (-0.05, 0.3) | IDE |
| Reardon et al., 2019 | Linear-PFOA | TSH | Multivariate mixed effect model (β) | 0.007 (P=0.368) | IDE |
| Aimuzi et al., 2020 | PFOS | TSH | Linear regression (β) | -0.016 (-0.099, 0.066) | IDE |
| Inoue et al., 2019 | PFOS | TSH | Multivariate linear regression (exp(β) - 1) × 100%) | 2.9 (-5.5, 12.1) | null |
| Lebeaux et al., 2019 | PFOS | TSH | Multivariable linear regression (β) | 0.02 (-0.24, 0.28) | IDE |
| Preston et al., 2018 | PFOS | TSH | Multivariable linear regression (exp(β) - 1) × 100%) | 0.90 (-7.27, 9.80) | null |
| Reardon et al., 2019 | PFOS | TSH | Multivariate mixed effect model (β) | 0.082 (P=0.069) | IDE |
| Wang et al., 2014 | PFOS | TSH | Linear regression (β) | -0.005 (-0.024, 0.013) | IDE |
| Webster et al., 2014 | PFOS | TSH | Mixed-effects linear model (β) | 0.1 (-0.03, 0.02) | IDE |
| Reardon et al., 2019 | Linear-PFOS | TSH | Multivariate mixed effect model (β) | 0.005 (P=0.792) | IDE |
| Reardon et al., 2019 | 1m-PFOS | TSH | Multivariate mixed effect model (β) | 0.379 (P=0.396) | IDE |
| Reardon et al., 2019 | ∑3m+4m-PFOS | TSH | Multivariate mixed effect model (β) | 0.026 (P=0.888) | IDE |
| Reardon et al., 2019 | *iso*-PFOS | TSH | Multivariate mixed effect model (β) | 0.053 (P=0.585) | IDE |
| Aimuzi et al., 2020 | PFNA | TSH | Linear regression (β) | -0.038 (-0.129, 0.054) | IDE |
| Inoue et al., 2019 | PFNA | TSH | Multivariate linear regression (exp(β) - 1) × 100%) | 0.8 (-4.9, 6.8) | null |
| Lebeaux et al., 2019 | PFNA | TSH | Multivariable linear regression (β) | -0.23 (-0.56, 0.10) | IDE |
| Preston et al., 2018 | PFNA | TSH | Multivariable linear regression (exp(β) - 1) × 100%) | -0.27 (-6.19, 6.03) | null |
| Reardon et al., 2019 | PFNA | TSH | Multivariate mixed effect model (β) | 0.005 (P=0.810) | IDE |
| Wang et al., 2013 | PFNA | TSH | Linear regression (β) | 0.165 (-0.023, 0.353) | IDE |
| Wang et al., 2014 | PFNA | TSH | Linear regression (β) | 0.033 (-0.046, 0.112) | IDE |
| Aimuzi et al., 2020 | PFDA | TSH | Linear regression (β) | -0.03 (-0.107, 0.048) | IDE |
| Inoue et al., 2019 | PFDA | TSH | Multivariate linear regression (exp(β) - 1) × 100%) | -1.9 (-7.7, 4.2) | null |
| Reardon et al., 2019 | PFDA | TSH | Multivariate mixed effect model (β) | 0.023 (P=0.647) | IDE |
| Wang et al., 2013 | PFDA | TSH | Linear regression (β) | 0.060 (-0.458, 0.578) | IDE |
| Lebeaux et al., 2019 | PFHxS | TSH | Multivariable linear regression (β) | -0.06 (-0.23, 0.11) | IDE |
| Preston et al., 2018 | PFHxS | TSH | Multivariable linear regression (exp(β) - 1) × 100%) | 2.89 (-2.12, 8.17) | null |
| Wang et al., 2013 | PFHxS | TSH | Linear regression (β) | 0.013 (-0.043, 0.070) | IDE |
| Webster et al., 2014 | PFHxS | TSH | Mixed-effects linear model (β) | 0.01 (-0.05, 0.07) | IDE |
| Xiao et al., 2020 | PFHxS | TSH | Multiple linear regression (exp(β) - 1) × 100%) | 7.4 (-11.8, 30.9) | null |
| Wang et al., 2013 | PFHpS | TSH | Linear regression (β) | 0.299 (-0.113, 0.710) | IDE |
| Inoue et al., 2019 | PFHpS | TSH | Multivariate linear regression (exp(β) - 1) × 100%) | 2.0 (-6.8, 11.5) | null |
| Aimuzi et al., 2020 | PFUnDA | TSH | Linear regression (β) | -0.037 (-0.119, 0.045) | IDE |
| Reardon et al., 2019 | PFUnDA | TSH | Multivariate mixed effect model (β) | -0.024 (P=0.901) | IDE |
| Wang et al., 2013 | PFUnDA | TSH | Linear regression (β) | 0.080 (-0.200, 0.360) | IDE |
| Wang et al., 2014 | PFUnDA | TSH | Linear regression (β) | 0.011 (-0.009, 0.030) | IDE |
| Xiao et al., 2020 | PFUnDA | TSH | Multiple linear regression (exp(β) - 1) × 100%) | -0.3 (-15.4, 17.4) | null |
| Wang et al., 2014 | PFDA | TSH | Linear regression (β) | 0.004 (-0.037, 0.045) | IDE |
| Aimuzi et al., 2020 | PFDoDA | TSH | Linear regression (β) | 0.03 (-0.074, 0.134) | IDE |
| Wang et al., 2014 | PFDoDA | TSH | Linear regression (β) | 0.365 (-0.215, 0.944) | IDE |
| Xiao et al., 2020 | PFDoDA | TSH | Multiple linear regression (exp(β) - 1) × 100%) | -2.5 (-9.2, 4.7) | null |
| Xiao et al., 2020 | NMeFOSAA | TSH | Multiple linear regression (exp(β) - 1) × 100%) | 8.0 (-2.7, 19.7) | null |
| Preston et al., 2018 | NEtFOSAA | TSH | Multivariable linear regression (exp(β) - 1) × 100%) | -0.06 (-4.31, 4.38) | null |
| Xiao et al., 2020 | NEtFOSAA | TSH | Multiple linear regression (exp(β) - 1) × 100%) | 5.7 (-7.3, 20.4) | null |
| Preston et al., 2018 | NMeFOSAA | TSH | Multivariable linear regression (exp(β) - 1) × 100%) | 0.57 (-6.27, 7.92) | null |
| Xiao et al., 2020 | PFOSA | TSH | Multiple linear regression (exp(β) - 1) × 100%) | 2.2 (-2.6, 7.1) | null |
| Aimuzi et al., 2020 | PFBS | TSH | Linear regression (β) | -0.001 (-0.065, 0.063) | IDE |
| Aimuzi et al., 2020 | PFHpA | TSH | Linear regression (β) | -0.014 (-0.073, 0.045) | IDE |
| Xiao et al., 2020 | PFHpA | TSH | Multiple linear regression (exp(β) - 1) × 100%) | -1.6 (-8.6, 6.0) | null |
| Xiao et al., 2020 | PFHpS | TSH | Multiple linear regression (exp(β) - 1) × 100%) | -0.6 (-6.1, 5.2) | null |

**Abbreviations:** β, beta coefficient; IDE, indeterminate; Q, quartile; r, rho; TSH, thyrotropin

**Bold**: statistically significant

# Tables present associations based on THs measured in maternal plasma or serum

**SUPPLEMENTAL TABLE 8: Evidence of PFAS association with thyroid hormones by thyroid autoantibody status in pregnancy**

| **Author** | **Exposure** | **Outcome** | **Statistical test (measure of association)** | **Association Value (95% CI or p-value)#** | **Direction** |
| --- | --- | --- | --- | --- | --- |
|  |  |  |  |  |  |
| **By Thyroid Antibody Status** | | | | | |
| Aimuzi et al., 2020 | PFNA | FT3 | Linear regression (β) | TPOAb (-): 0.128 (-0.009, 0.265) | IDE |
|  |  |  |  | **TPOAb (+): 0.491 (0.033, 0.95)** | **+** |
| Itoh et al., 2019 | PFNA | FT3 | Linear regression (β) | TAb (-): 0.032 (-0.023, 0.086) | IDE |
|  |  |  |  | **TAb (+): 0.180 (0.013, 0.347)** | **+** |
| Aimuzi et al., 2020 | PFHxS | FT3 | Linear regression (β) | **TPOAb (-): 0.204 (0.058, 0.351)** | **+** |
|  |  |  |  | TPOAb (+): 0.042 (-0.502, 0.587) | IDE |
| Itoh et al., 2019 | PFHxS | FT3 | Linear regression (β) | **TAb (-): 0.043 (0.003, 0.083)** | **+** |
|  |  |  |  | TAb (+): 0.040 (-0.090, 0.170) | IDE |
| Aimuzi et al., 2020 | PFNA | FT4 | Linear regression (β) | TPOAb (-): 0.001 (-0.096, 0.099) | IDE |
|  |  |  |  | **TPOAb (+): 0.318 (0.018, 0.618)** | **+** |
| Lebeaux et al., 2019 | PFHxS | FT4 | Multivariable linear regression (β) | **TgAb (-): 0.05 (0.01, 0.09)** | **+** |
|  |  |  |  | TgAb (+): -0.02 (-0.06, 0.03) | IDE |
| Reardon et al., 2019 | PFUnDA | FT4 | Multivariate mixed effect model (β) | Normal TPOAb: -0.011 (-0.087, 0.066) | IDE |
|  |  |  |  | **High TPOAb: -0.240 (-0.456, -0.025)** | **-** |
| Webster et al., 2014 | PFOA | TSH | Mixed-effects linear model (β) | Normal TPOAb: 0.07 (-0.1, 0.2) | IDE |
|  |  |  |  | **High TPOAb: 0.7 (0.09, 1)** | **+** |
| Webster et al., 2014 | PFOS | TSH | Mixed-effects linear model (β) | Normal TPOAb: 0.07 (-0.06, 0.2) | IDE |
|  |  |  |  | **High TPOAb: 0.9 (0.2, 2)** | **+** |
| Webster et al., 2014 | PFNA | TSH | Mixed-effects linear model (β) | Normal TPOAb: 0.1 (-0.05, 0.3) | IDE |
|  |  |  |  | **High TPOAb: 0.6 (0.1, 1)** | **+** |
| Reardon et al., 2019 | 1m-PFOS | TSH | Multivariate mixed effect model (β) | **1^st^ trimester normal TPOAb:** **1.97 (0.198, 3.74)** | **+** |
|  |  |  |  | 1^st^ trimester high TPOAb: −0.819 (−3.13, 1.49) | IDE |
|  |  |  |  | **2^nd^ trimester normal TPOAb:** **1.36 (0.218, 2.51)** | **+** |
|  |  |  |  | 2^nd^ trimester high TPOAb: -1.427 (-3.32, 0.468) | IDE |
|  |  |  |  | 3^rd^ trimester normal TPOAb: 0.754 (-0.273, 1.78) | IDE |
|  |  |  |  | **3^rd^ trimester high TPOAb:** **-2.03 (-3.88, -0.190)** | **-** |
| Preston et al., 2018 | PFNA | TSH | Multivariable linear regression (exp(β) - 1) × 100%) | TPOAb (-): 0.78 (-5.46, 7.44) | null |
|  |  |  |  | **TPOAb (+): -16.1 (-27.7, -2.56)** | **-** |
| Aimuzi et al., 2020 | PFUnDA | TSH | Linear regression (β) | TPOAb (-): -0.005 (-0.094, 0.085) | IDE |
|  |  |  |  | **TPOAb (+): -0.281 (-0.531, -0.032)** | **-** |
| Preston et al., 2018 | PFOA | TSH | Multivariable linear regression (exp(β) - 1) × 100%) | TPOAb (-): 0.88 (-9.22, 12.1) | null |
|  |  |  |  | TPOAb (+): -19.0 (-35.1, 1.15) | - |
| Preston et al., 2018 | PFOS | TSH | Multivariable linear regression (exp(β) - 1) × 100%) | TPOAb (-): 2.84 (-6.02, 12.5) | null |
|  |  |  |  | TPOAb (+): -16.4 (-29.8, -0.38) | - |
| Itoh et al., 2019 | PFAS (total) | FT3 | Linear regression (β) | TAb (-): 0.071 (-0.001, 0.144) | IDE |
|  |  |  |  | TAb (+): 0.190 (-0.020, 0.399) | IDE |
| Aimuzi et al., 2020 | PFOA | FT3 | Linear regression (β) | TPOAb (-): 0.079 (-0.079, 0.238) | IDE |
|  |  |  |  | TPOAb (+): 0.222 (-0.255, 0.699) | IDE |
| Itoh et al., 2019 | PFOA | FT3 | Linear regression (β) | TAb (-): 0.021 (-0.029, 0.071) | IDE |
|  |  |  |  | TAb (+): 0.110 (-0.009, 0.229) | IDE |
| Lebeaux et al., 2019 | PFOA | FT3 | Multivariable linear regression (β) | TPOAb ≤ median: -0.03 (-0.06, 0.00) | IDE |
|  |  |  |  | TPOAb > median: 0.004 (-0.03, 0.04) | IDE |
| Lebeaux et al., 2019 | PFOA | T3 | Multivariable linear regression (β) | TPOAb ≤ median: -0.03 (-0.09, 0.02) | IDE |
|  |  |  |  | TPOAb > median: 0.03 (-0.04, 0.09) | IDE |
| Lebeaux et al., 2019 | PFOA | FT3 | Multivariable linear regression (β) | TgAb (-): -0.01 (-0.04, 0.02) | IDE |
|  |  |  |  | TgAb (+): -0.03 (-0.08, 0.01) | IDE |
| Lebeaux et al., 2019 | PFOA | T3 | Multivariable linear regression (β) | TgAb (-): -0.01 (-0.06, 0.04) | IDE |
|  |  |  |  | TgAb (+): -0.02 (-0.09, 0.05) | IDE |
| Aimuzi et al., 2020 | PFOS | FT3 | Linear regression (β) | TPOAb (-): 0.081 (-0.04, 0.202) | IDE |
|  |  |  |  | TPOAb (+): 0.287 (-0.129, 0.703) | IDE |
| Itoh et al., 2019 | PFOS | FT3 | Linear regression (β) | TAb (-): 0.055 (-0.004, 0.115) | IDE |
|  |  |  |  | TAb (+): 0.149 (-0.048, 0.348) | IDE |
| Lebeaux et al., 2019 | PFOS | FT3 | Multivariable linear regression (β) | TPOAb ≤ median: -0.04 (-0.08, 0.00) | IDE |
|  |  |  |  | TPOAb > median: -0.02 (-0.06, 0.02) | IDE |
| Lebeaux et al., 2019 | PFOS | T3 | Multivariable linear regression (β) | TPOAb ≤ median: -0.05 (-0.12, 0.01) | IDE |
|  |  |  |  | TPOAb > median: 0.01 (-0.05, 0.08) | IDE |
| Lebeaux et al., 2019 | PFOS | FT3 | Multivariable linear regression (β) | TgAb (-): -0.02 (-0.05, 0.02) | IDE |
|  |  |  |  | TgAb (+): -0.06 (-0.11, -0.01) | IDE |
| Lebeaux et al., 2019 | PFOS | T3 | Multivariable linear regression (β) | TgAb (-): -0.02 (-0.08, 0.03) | IDE |
|  |  |  |  | TgAb (+): -0.04 (-0.12, 0.04) | IDE |
| Lebeaux et al., 2019 | PFNA | FT3 | Multivariable linear regression (β) | TPOAb ≤ median: -0.03 (-0.08, 0.02) | IDE |
|  |  |  |  | TPOAb > median: -0.01 (-0.06, 0.04) | IDE |
| Lebeaux et al., 2019 | PFNA | T3 | Multivariable linear regression (β) | TPOAb ≤ median: -0.07 (-0.15, 0.02) | IDE |
|  |  |  |  | TPOAb > median: 0.004 (-0.08, 0.09) | IDE |
| Lebeaux et al., 2019 | PFNA | FT3 | Multivariable linear regression (β) | TgAb (-): -0.04 (-0.08, 0.01) | IDE |
|  |  |  |  | TgAb (+): 0.02 (-0.04, 0.08) | IDE |
| Lebeaux et al., 2019 | PFNA | T3 | Multivariable linear regression (β) | TgAb (-): -0.04 (-0.12, 0.03) | IDE |
|  |  |  |  | TgAb (+): 0.01 (-0.08, 0.11) | IDE |
| Aimuzi et al., 2020 | PFDA | FT3 | Linear regression (β) | TPOAb(-): 0.073 (-0.038, 0.184) | IDE |
|  |  |  |  | TPOAb(+): 0.307 (-0.08, 0.693) | IDE |
| Itoh et al., 2019 | PFDA | FT3 | Linear regression (β) | TAb (-): 0.043 (-0.004, 0.090) | IDE |
|  |  |  |  | TAb (+): 0.103 (-0.035, 0.242) | IDE |
| Lebeaux et al., 2019 | PFHxS | FT3 | Multivariable linear regression (β) | TPOAb ≤ median: -0.02 (-0.05, 0.00) | IDE |
|  |  |  |  | TPOAb > median: -0.01 (-0.04, 0.02) | IDE |
| Lebeaux et al., 2019 | PFHxS | T3 | Multivariable linear regression (β) | TPOAb ≤ median: -0.03 (-0.07, 0.01) | IDE |
|  |  |  |  | TPOAb > median: 0.01 (-0.03, 0.06) | IDE |
| Lebeaux et al., 2019 | PFHxS | FT3 | Multivariable linear regression (β) | TgAb (-): -0.01 (-0.04, 0.01) | IDE |
|  |  |  |  | TgAb (+): -0.02 (-0.05, 0.00) | IDE |
| Lebeaux et al., 2019 | PFHxS | T3 | Multivariable linear regression (β) | TgAb (-): -0.02 (-0.06, 0.02) | IDE |
|  |  |  |  | TgAb (+): -0.01 (-0.06, 0.03) | IDE |
| Aimuzi et al., 2020 | PFDoDA | FT3 | Linear regression (β) | TPOAb (-): -0.015 (-0.195, 0.165) | IDE |
|  |  |  |  | TPOAb (+): 0.107 (-0.445, 0.66) | IDE |
| Itoh et al., 2019 | PFDoDA | FT3 | Linear regression (β) | TAb (-): 0.024 (-0.018, 0.065) | IDE |
|  |  |  |  | TAb (+): -0.023 (-0.154, 0.109) | IDE |
| Itoh et al., 2019 | PFTrDA | FT3 | Linear regression (β) | TAb (-): 0.018 (-0.023, 0.060) | IDE |
|  |  |  |  | TAb (+): -0.005 (-0.139, 0.130) | IDE |
| Aimuzi et al., 2020 | PFUnDA | FT3 | Linear regression (β) | TPOAb (-): 0.035 (-0.082, 0.151) | IDE |
|  |  |  |  | TPOAb (+): 0.236 (-0.17, 0.642) | IDE |
| Itoh et al., 2019 | PFUnDA | FT3 | Linear regression (β) | TAb (-): 0.034 (-0.003, 0.072) | IDE |
|  |  |  |  | TAb (+): 0.040 (-0.112, 0.192) | IDE |
| Aimuzi et al., 2020 | PFBS | FT3 | Linear regression (β) | TPOAb (-): -0.016 (-0.109, 0.077) | IDE |
|  |  |  |  | TPOAb (+): -0.028 (-0.222, 0.165) | IDE |
| Aimuzi et al., 2020 | PFHpA | FT3 | Linear regression (β) | TPOAb (-): -0.024 (-0.097, 0.049) | IDE |
|  |  |  |  | TPOAb (+): 0.073 (-0.12, 0.266) | IDE |
| Itoh et al., 2019 | PFAS (total) | FT4 | Linear regression (β) | TAb (-): 0.074 (-0.007, 0.155) | IDE |
|  |  |  |  | TAb (+): -0.007 (-0.204, 0.190) | IDE |
| Aimuzi et al., 2020 | PFOA | FT4 | Linear regression (β) | TPOAb (-): 0.106 (-0.008, 0.22) | IDE |
|  |  |  |  | TPOAb (+): 0.138 (-0.204, 0.479) | IDE |
| Itoh et al., 2019 | PFOA | FT4 | Linear regression (β) | TAb (-): 0.014 (-0.042, 0.070) | IDE |
|  |  |  |  | TAb (+): 0.034 (-0.078, 0.145) | IDE |
| Lebeaux et al., 2019 | PFOA | FT4 | Multivariable linear regression (β) | TPOAb ≤ median: -0.02 (-0.07, 0.04) | IDE |
|  |  |  |  | TPOAb > median: -0.002 (-0.07, 0.06) | IDE |
| Lebeaux et al., 2019 | PFOA | T4 | Multivariable linear regression (β) | TPOAb ≤ median: -0.01 (-0.10, 0.08) | IDE |
|  |  |  |  | TPOAb > median: -0.05 (-0.16, 0.05) | IDE |
| Lebeaux et al., 2019 | PFOA | FT4 | Multivariable linear regression (β) | TgAb (-): -0.0001 (-0.05, 0.05) | IDE |
|  |  |  |  | TgAb (+): -0.04 (-0.11, 0.03) | IDE |
| Lebeaux et al., 2019 | PFOA | T4 | Multivariable linear regression (β) | TgAb (-): -0.03 (-0.11, 0.06) | IDE |
|  |  |  |  | TgAb (+): -0.05 (-0.17, 0.07) | IDE |
| Webster et al., 2014 | PFOA | FT4 | Mixed-effects linear model (β) | Normal TPOAb: -0.03 (-0.3, 0.2) | IDE |
|  |  |  |  | High TPOAb: -0.4 (-1, 0.5) | IDE |
| Aimuzi et al., 2020 | PFOS | FT4 | Linear regression (β) | TPOAb (-): 0.006 (-0.081, 0.092) | IDE |
|  |  |  |  | TPOAb (+): 0.196 (-0.083, 0.475) | IDE |
| Itoh et al., 2019 | PFOS | FT4 | Linear regression (β) | TAb (-): 0.066 (-0.001, 0.133) | IDE |
|  |  |  |  | TAb (+): -0.021 (-0.204, 0.163) | IDE |
| Lebeaux et al., 2019 | PFOS | FT4 | Multivariable linear regression (β) | TPOAb ≤ median: 0.03 (-0.03, 0.10) | IDE |
|  |  |  |  | TPOAb > median: 0.02 (-0.04, 0.09) | IDE |
| Lebeaux et al., 2019 | PFOS | T4 | Multivariable linear regression (β) | TPOAb ≤ median: 0.04 (-0.06, 0.15) | IDE |
|  |  |  |  | TPOAb > median: -0.03 (-0.14, 0.08) | IDE |
| Lebeaux et al., 2019 | PFOS | FT4 | Multivariable linear regression (β) | TgAb (-): 0.05 (0.00, 0.11) | IDE |
|  |  |  |  | TgAb (+): -0.04 (-0.12, 0.04) | IDE |
| Lebeaux et al., 2019 | PFOS | T4 | Multivariable linear regression (β) | TgAb (-): 0.03 (-0.07, 0.12) | IDE |
|  |  |  |  | TgAb (+): -0.07 (-0.20, 0.07) | IDE |
| Webster et al., 2014 | PFOS | FT4 | Mixed-effects linear model (β) | Normal TPOAb: 0.05 (-0.1, 0.2) | IDE |
|  |  |  |  | High TPOAb: -0.7 (-2, 0.3) | IDE |
| Itoh et al., 2019 | PFNA | FT4 | Linear regression (β) | TAb (-): 0.038 (-0.023, 0.099) | IDE |
|  |  |  |  | TAb (+): -0.018 (-0.178, 0.142) | IDE |
| Lebeaux et al., 2019 | PFNA | FT4 | Multivariable linear regression (β) | TPOAb ≤ median: 0.02 (-0.07, 0.10) | IDE |
|  |  |  |  | TPOAb > median: -0.04 (-0.12, 0.05) | IDE |
| Lebeaux et al., 2019 | PFNA | T4 | Multivariable linear regression (β) | TPOAb ≤ median: -0.06 (-0.20, 0.07) | IDE |
|  |  |  |  | TPOAb > median: -0.09 (-0.22, 0.05) | IDE |
| Lebeaux et al., 2019 | PFNA | FT4 | Multivariable linear regression (β) | TgAb (-): 0.03 (-0.04, 0.11) | IDE |
|  |  |  |  | TgAb (+): -0.07 (-0.16, 0.03) | IDE |
| Lebeaux et al., 2019 | PFNA | T4 | Multivariable linear regression (β) | TgAb (-): -0.08 (-0.21, 0.04) | IDE |
|  |  |  |  | TgAb (+): -0.02 (-0.18, 0.14) | IDE |
| Webster et al., 2014 | PFNA | FT4 | Mixed-effects linear model (β) | Normal TPOAb: 0.004 (-0.2, 0.2) | IDE |
|  |  |  |  | High TPOAb: -0.3 (-1, 0.4) | IDE |
| Aimuzi et al., 2020 | PFDA | FT4 | Linear regression (β) | TPOAb (-): 0.013 (-0.068, 0.094) | IDE |
|  |  |  |  | TPOAb (+): 0.244 (-0.013, 0.502) | IDE |
| Itoh et al., 2019 | PFDA | FT4 | Linear regression (β) | TAb (-): 0.029 (-0.024, 0.081) | IDE |
|  |  |  |  | TAb (+): -0.050 (-0.176, 0.077) | IDE |
| Aimuzi et al., 2020 | PFHxS | FT4 | Linear regression (β) | TPOAb (-): 0.091 (-0.015, 0.197) | IDE |
|  |  |  |  | TPOAb (+): 0.261 (-0.056, 0.578) | IDE |
| Itoh et al., 2019 | PFHxS | FT4 | Linear regression (β) | TAb (-): 0.039 (-0.006, 0.084) | IDE |
|  |  |  |  | TAb (+): 0.025 (-0.091, 0.141) | IDE |
| Lebeaux et al., 2019 | PFHxS | FT4 | Multivariable linear regression (β) | TPOAb ≤ median: 0.02 (-0.02, 0.06) | IDE |
|  |  |  |  | TPOAb > median: 0.02 (-0.03, 0.06) | IDE |
| Lebeaux et al., 2019 | PFHxS | T4 | Multivariable linear regression (β) | TPOAb ≤ median: 0.01 (-0.06, 0.07) | IDE |
|  |  |  |  | TPOAb > median: 0.01 (-0.07, 0.08) | IDE |
| Lebeaux et al., 2019 | PFHxS | T4 | Multivariable linear regression (β) | TgAb (-): -0.005 (-0.07, 0.06) | IDE |
|  |  |  |  | TgAb (+): 0.01 (-0.07, 0.08) | IDE |
| Webster et al., 2014 | PFHxS | FT4 | Mixed-effects linear model (β) | Normal TPOAb: -0.01 (-0.1, 0.08) | IDE |
|  |  |  |  | High TPOAb: -0.5 (-1, 0.3) | IDE |
| Aimuzi et al., 2020 | PFDoDA | FT4 | Linear regression (β) | TPOAb (-): -0.097 (-0.208, 0.013) | IDE |
|  |  |  |  | TPOAb (+): 0.021 (-0.318, 0.359) | IDE |
| Itoh et al., 2019 | PFDoDA | FT4 | Linear regression (β) | TAb (-): 0.015 (-0.032, 0.062) | IDE |
|  |  |  |  | TAb (+): -0.040 (-0.156, 0.077) | IDE |
| Itoh et al., 2019 | PFTrDA | FT4 | Linear regression (β) | TAb (-): 0.016 (-0.030, 0.062) | IDE |
|  |  |  |  | TAb (+): 0.020 (-0.100, 0.139) | IDE |
| Aimuzi et al., 2020 | PFUnDA | FT4 | Linear regression (β) | TPOAb (-): -0.004 (-0.09, 0.083) | IDE |
|  |  |  |  | TPOAb (+): 0.208 (-0.063, 0.479) | IDE |
| Itoh et al., 2019 | PFUnDA | FT4 | Linear regression (β) | TAb (-): 0.037 (-0.005, 0.079) | IDE |
|  |  |  |  | TAb (+): 0.016 (-0.120, 0.152) | IDE |
| Aimuzi et al., 2020 | PFBS | FT4 | Linear regression (β) | TPOAb (-): 0.039 (-0.031, 0.109) | IDE |
|  |  |  |  | TPOAb (+): -0.116 (-0.295, 0.062) | IDE |
| Aimuzi et al., 2020 | PFHpA | FT4 | Linear regression (β) | TPOAb (-): -0.046 (-0.109, 0.018) | IDE |
|  |  |  |  | TPOAb (+): 0.012 (-0.161, 0.185) | IDE |
| Itoh et al., 2019 | PFAS (total) | TSH | Linear regression (β) | TAb (-): 0.376 (-0.952, 0.200) | IDE |
|  |  |  |  | TAb (+): -0.170 (-1.866, 1.527) | IDE |
| Aimuzi et al., 2020 | PFOA | TSH | Linear regression (β) | TPOAb (-): -0.02 (-0.138, 0.098) | IDE |
|  |  |  |  | TPOAb (+): -0.03 (-0.344, 0.283) | IDE |
| Itoh et al., 2019 | PFOA | TSH | Linear regression (β) | TAb (-): -0.046 (-0.439, 0.348) | IDE |
|  |  |  |  | TAb (+): -0.258 (-1.219, 0.704) | IDE |
| Lebeaux et al., 2019 | PFOA | TSH | Multivariable linear regression (β) | TPOAb ≤ median: 0.13 (-0.17, 0.42) | IDE |
|  |  |  |  | TPOAb > median: 0.02 (-0.34, 0.37) | IDE |
| Lebeaux et al., 2019 | PFOA | TSH | Multivariable linear regression (β) | TgAb (-): 0.12 (-0.16, 0.40) | IDE |
|  |  |  |  | TgAb (+): 0.01 (-0.38, 0.40) | IDE |
| Aimuzi et al., 2020 | PFOS | TSH | Linear regression (β) | TPOAb (-): 0.008 (-0.081, 0.097) | IDE |
|  |  |  |  | TPOAb (+): -0.198 (-0.455, 0.059) | IDE |
| Itoh et al., 2019 | PFOS | TSH | Linear regression (β) | TAb (-): -0.373 (-0.847, 0.101) | IDE |
|  |  |  |  | TAb (+): 0.046 (-1.535, 1.627) | IDE |
| Lebeaux et al., 2019 | PFOS | TSH | Multivariable linear regression (β) | TPOAb ≤ median: -0.06 (-0.41, 0.29) | IDE |
|  |  |  |  | TPOAb > median: 0.07 (-0.29, 0.44) | IDE |
| Lebeaux et al., 2019 | PFOS | TSH | Multivariable linear regression (β) | TgAb (-): -0.002 (-0.31, 0.31) | IDE |
|  |  |  |  | TgAb (+): 0.02 (-0.42, 0.46) | IDE |
| Aimuzi et al., 2020 | PFNA | TSH | Linear regression (β) | TPOAb (-): -0.006 (-0.106, 0.095) | IDE |
|  |  |  |  | TPOAb (+): -0.213 (-0.489, 0.063) | IDE |
| Itoh et al., 2019 | PFNA | TSH | Linear regression (β) | TAb (-): -0.151 (-0.583, 0.280) | IDE |
|  |  |  |  | TAb (+): -0.410 (-1.782, 0.962) | IDE |
| Lebeaux et al., 2019 | PFNA | TSH | Multivariable linear regression (β) | TPOAb ≤ median: -0.27 (-0.72, 0.18) | IDE |
|  |  |  |  | TPOAb > median: -0.20 (-0.66, 0.26) | IDE |
| Lebeaux et al., 2019 | PFNA | TSH | Multivariable linear regression (β) | TgAb (-): -0.40 (-0.81, 0.01) | IDE |
|  |  |  |  | TgAb (+): 0.03 (-0.50, 0.55) | IDE |
| Aimuzi et al., 2020 | PFDA | TSH | Linear regression (β) | TPOAb (-): -0.006 (-0.089, 0.078) | IDE |
|  |  |  |  | TPOAb (+): -0.21 (-0.448, 0.027) | IDE |
| Itoh et al., 2019 | PFDA | TSH | Linear regression (β) | TAb (-): -0.114 (-0.485, 0.258) | IDE |
|  |  |  |  | TAb (+): 0.087 (-1.105, 1.189) | IDE |
| Aimuzi et al., 2020 | PFHxS | TSH | Linear regression (β) | TPOAb (-): -0.104 (-0.213, 0.006) | IDE |
|  |  |  |  | TPOAb (+): -0.168 (-0.46, 0.124) | IDE |
| Itoh et al., 2019 | PFHxS | TSH | Linear regression (β) | TAb (-): 0.033 (-0.289, 0.354) | IDE |
|  |  |  |  | TAb (+): 0.082 (-0.921, 1.085) | IDE |
| Lebeaux et al., 2019 | PFHxS | TSH | Multivariable linear regression (β) | TPOAb ≤ median: 0.03 (-0.18, 0.25) | IDE |
|  |  |  |  | TPOAb > median: -0.20 (-0.44, 0.04) | IDE |
| Lebeaux et al., 2019 | PFHxS | TSH | Multivariable linear regression (β) | TgAb (-): -0.03 (-0.26, 0.19) | IDE |
|  |  |  |  | TgAb (+): -0.11 (-0.35, 0.14) | IDE |
| Preston et al., 2018 | PFHxS | TSH | Multivariable linear regression (exp(β) - 1) × 100%) | TPOAb (-): 3.04 (-2.51, 8.91) | null |
|  |  |  |  | TPOAb (+): -4.77 (-12.7, 3.88) | null |
| Webster et al., 2014 | PFHxS | TSH | Mixed-effects linear model (β) | Normal TPOAb: 0.01 (-0.05, 0.08) | IDE |
|  |  |  |  | High TPOAb: 0.02 (-0.6, 0.6) | IDE |
| Aimuzi et al., 2020 | PFDoDA | TSH | Linear regression (β) | TPOAb (-): 0.033 (-0.08, 0.147) | IDE |
|  |  |  |  | TPOAb (+): 0.016 (-0.277, 0.308) | IDE |
| Itoh et al., 2019 | PFDoDA | TSH | Linear regression (β) | TAb (-): 0.040 (-0.291, 0.371) | IDE |
|  |  |  |  | TAb (+): 0.542 (-0.450, 1.533) | IDE |
| Itoh et al., 2019 | PFTrDA | TSH | Linear regression (β) | TAb (-): 0.076 (-0.250, 0.402) | IDE |
|  |  |  |  | TAb (+): 0.283 (-0.743, 1.310) | IDE |
| Itoh et al., 2019 | PFUnDA | TSH | Linear regression (β) | TAb (-): -0.026 (-0.325, 0.272) | IDE |
|  |  |  |  | TAb (+): -0.407 (-1.570, 0.756) | IDE |
| Aimuzi et al., 2020 | PFBS | TSH | Linear regression (β) | TPOAb (-): 0.005 (-0.067, 0.077) | IDE |
|  |  |  |  | TPOAb (+): -0.071 (-0.226, 0.084) | IDE |
| Aimuzi et al., 2020 | PFHpA | TSH | Linear regression (β) | TPOAb (-): -0.011 (-0.077, 0.055) | IDE |
|  |  |  |  | TPOAb (+): -0.035 (-0.192, 0.122) | IDE |
| Preston et al., 2018 | NEtFOSAA | TSH | Multivariable linear regression (exp(β) - 1) × 100%) | TPOAb (-): 0.24 (-4.16, 4.83) | null |
|  |  |  |  | TPOAb (+): -4.87 (-15.9, 7.56) | null |
| Preston et al., 2018 | NMeFOSAA | TSH | Multivariable linear regression (exp(β) - 1) × 100%) | TPOAb (-): 0.30 (-6.92, 8.09) | null |
|  |  |  |  | TPOAb (+): -5.82 (-19.0, 9.58) | null |

**Abbreviations:** β, beta coefficient; fT3, free triiodothyronine; fT4, free thyroxine; IDE, indeterminate; T3, triiodothyronine; T4, thyroxine; TAb, thyroid antibody; TgAb, Thyroglobulin; TPOAb, thyroid peroxidase antibody; TSH, thyrotropin

**Bold**: statistically significant

# Tables present associations based on THs measured in maternal plasma or serum

**Per- and polyfluoroalkyl substances (PFAS) compound abbreviations**

| 1m-PFOS | perfluoro-1-methylheptanesulfonate |
| --- | --- |
| ∑3m+4m-PFOS | branched isomers of perfluorooctane sulfonate |
| 5m-PFOS | perfluoro-5-methylheptanesulfonate |
| 6:2 FTS | 6:2 fluorotelomer sulfonate |
| Cl-PFESA | chlorinated polyfluorinated ether sulfonates |
| FHUEA | 2H-perfluoro-2-octenoate |
| FOUEA | 2H-perfluoro-2-decenoate |
| FDUEA | 2H-perfluoro-2-dodecenoate |
| 6m-PFOS | perfluoro-6-methylheptylsulfonate |
| Br-PFOS | branched perfluorooctane sulfonate |
| iso-PFOS | perfluoroisopropyl |
| long-chain PFCAs | sum of perfluorooctanoate, perfluorononanoate, perfluorodecanoate, perfluoroundecanoate, perfluorododecanoate, perfluorotridecanoate |
| long-chain PFSAs | sum of perfluorohexanesulfonate and 1m-, 3m-, 4m-, 5m-, 6m- perfluorooctane sulfonate and L- perfluorooctane sulfonate |
| Σm-PFOA | represented by 6m-perfluorooctanoic acid |
| Σm-PFOS | sum of five branched perfluorooctane sulfonate isomers (1m-, 3m-, 4m-, 5m-, 6m-PFOS) |
| NEtFOSAA/Et-PFOSA-AcOH | 2-(N-ethyl-perfluorooctane sulfonamido) acetate |
| NMeFOSAA/Me-PFOSA-AcOH | 2-(N-Methyl-perfluorooctane sulfonamido) acetate |
| NEtFOSA | N-ethyl perfluorooctane sulfonamide |
| NMeFOSA | N-methyl perfluorooctane sulfonamide |
| PFBS | perfluorobutane sulfonate |
| ∑PFCs | sum of perfluorinated compounds |
| PFDA | perfluorodecanoic acid |
| PFDS | perfluorodecane sulfonic acid |
| PFDoDA | perfluorododecanoate |
| PFHpA | perfluoroheptanoate |
| PFHpS | perfluoroheptane sulfonate |
| PFHxA | perfluoroheptanoic acid |
| PFHxS | perfluorohexane sulfonic acid |
| PFNA | perfluorononanoic acid |
| PFOA | perfluorooctanoic acid |
| PFOS | perfluorooctane sulfonate |
| PFOSA | perfluorooctane sulfonamide |
| PFPeA | perfluoropentacoic acid |
| PFTA | perfluorotetradecanoate |
| PFTeDA | perfluorotetradecanoic acid |
| PFTrDA | perfluorotridecanoic acid |
| PFUA | perfluoroundecanoic acid |
| PFUnDA | perfluoroundecanoic acid |
| Short-chain PFCAs | sum of perfluorobutanoate, perfluoropentacoic acid, perfluorohexanoate, perfluoroheptanoate |
| Short-chain PFSAs | represented by perfluorobutane sulfonate |
| Total PFCAs | sum of perfluorooctanoic acid, perfluorononanoic acid, perfluorodecanoate, perfluoroundecanoate, perfluorododecanoate, perfluorotridecanoate |
| Total PFSAs | sum of perfluoroalkyl sulfonates |

**References**

1. Thomas LE, Pencina MJ: **Do Not Over (P) Value Your Research Article**. *JAMA Cardiol* 2016, **1**(9):1055.

2. Wasserstein RL, Lazar NA: **The ASA Statement on p-Values: Context, Process, and Purpose**. *The American Statistician* 2016, **70**(2):129-133.

3. Altman N, Krzywinski M: **Interpreting P values**. *Nature Methods* 2017, **14**(3):213-214.

4. Aguinis H, Vassar M, Wayant C: **On reporting and interpreting statistical significance and p values in medical research**. *BMJ Evid Based Med* 2019.

5. Aimuzi R, Luo K, Huang R, Huo X, Nian M, Ouyang F, Du Y, Feng L, Wang W, Zhang J: **Perfluoroalkyl and polyfluroalkyl substances and maternal thyroid hormones in early pregnancy**. *Environmental pollution (Barking, Essex : 1987)* 2020, **264**:114557.

6. Berg V, Nøst TH, Hansen S, Elverland A, Veyhe AS, Jorde R, Odland J, Sandanger TM: **Assessing the relationship between perfluoroalkyl substances, thyroid hormones and binding proteins in pregnant women; a longitudinal mixed effects approach**. *Environment international* 2015, **77**:63-69.

7. Chan E, Burstyn I, Cherry N, Bamforth F, Martin JW: **Perfluorinated acids and hypothyroxinemia in pregnant women**. *Environ Res* 2011, **111**(4):559-564.

8. Inoue K, Ritz B, Andersen SL, Ramlau-Hansen CH, Hoyer BB, Bech BH, Henriksen TB, Bonefeld-Jorgensen EC, Olsen J, Liew Z: **Perfluoroalkyl Substances and Maternal Thyroid Hormones in Early Pregnancy; Findings in the Danish National Birth Cohort**. *Environ Health Perspect* 2019, **127**(11):117002.

9. Itoh S, Araki A, Miyashita C, Yamazaki K, Goudarzi H, Minatoya M, Ait Bamai Y, Kobayashi S, Okada E, Kashino I *et al*: **Association between perfluoroalkyl substance exposure and thyroid hormone/thyroid antibody levels in maternal and cord blood: The Hokkaido Study**. *Environment international* 2019, **133**(Pt A):105139.

10. Jensen RC, Glintborg D, Timmermann CAG, Nielsen F, Kyhl HB, Andersen HR, Grandjean P, Jensen TK, Andersen M: **Perfluoroalkyl substances and glycemic status in pregnant Danish women: The Odense Child Cohort**. *Environment international* 2018, **116**:101-107.

11. Lebeaux RM, Doherty BT, Gallagher LG, Zoeller RT, Hoofnagle AN, Calafat AM, Karagas MR, Yolton K, Chen A, Lanphear BP *et al*: **Maternal serum perfluoroalkyl substance mixtures and thyroid hormone concentrations in maternal and cord sera: The HOME Study**. *Environ Res* 2020, **185**:109395.

12. Li J, Yao J, Xia W, Dai J, Liu H, Pan Y, Xu S, Lu S, Jin S, Li Y *et al*: **Association between exposure to per- and polyfluoroalkyl substances and blood glucose in pregnant women**. *International journal of hygiene and environmental health* 2020, **230**:113596.

13. Liu X, Zhang L, Chen L, Li J, Wang Y, Wang J, Meng G, Chi M, Zhao Y, Chen H *et al*: **Structure-based investigation on the association between perfluoroalkyl acids exposure and both gestational diabetes mellitus and glucose homeostasis in pregnant women**. *Environment international* 2019, **127**:85-93.

14. Matilla-Santander N, Valvi D, Lopez-Espinosa MJ, Manzano-Salgado CB, Ballester F, Ibarluzea J, Santa-Marina L, Schettgen T, Guxens M, Sunyer J *et al*: **Exposure to Perfluoroalkyl Substances and Metabolic Outcomes in Pregnant Women: Evidence from the Spanish INMA Birth Cohorts**. *Environ Health Perspect* 2017, **125**(11):117004.

15. Mehta SS, James-Todd T, Applebaum KM, Bellavia A, Coleman-Phox K, Adler N, Laraia B, Epel E, Parry E, Wang M *et al*: **Persistent organic pollutants and maternal glycemic outcomes in a diverse pregnancy cohort of overweight women**. *Environ Res* 2021, **193**:110551.

16. Preston EV, Webster TF, Oken E, Claus Henn B, McClean MD, Rifas-Shiman SL, Pearce EN, Braverman LE, Calafat AM, Ye X *et al*: **Maternal Plasma per- and Polyfluoroalkyl Substance Concentrations in Early Pregnancy and Maternal and Neonatal Thyroid Function in a Prospective Birth Cohort: Project Viva (USA)**. *Environ Health Perspect* 2018, **126**(2):027013.

17. Preston EV, Rifas-Shiman SL, Hivert MF, Zota AR, Sagiv SK, Calafat AM, Oken E, James-Todd T: **Associations of Per- and Polyfluoroalkyl Substances (PFAS) With Glucose Tolerance During Pregnancy in Project Viva**. *J Clin Endocrinol Metab* 2020, **105**(8).

18. Rahman ML, Zhang C, Smarr MM, Lee S, Honda M, Kannan K, Tekola-Ayele F, Buck Louis GM: **Persistent organic pollutants and gestational diabetes: A multi-center prospective cohort study of healthy US women**. *Environment international* 2019, **124**:249-258.

19. Reardon AJF, Khodayari Moez E, Dinu I, Goruk S, Field CJ, Kinniburgh DW, MacDonald AM, Martin JW: **Longitudinal analysis reveals early-pregnancy associations between perfluoroalkyl sulfonates and thyroid hormone status in a Canadian prospective birth cohort**. *Environment international* 2019, **129**:389-399.

20. Ren Y, Jin L, Yang F, Liang H, Zhang Z, Du J, Song X, Miao M, Yuan W: **Concentrations of perfluoroalkyl and polyfluoroalkyl substances and blood glucose in pregnant women**. *Environ Health* 2020, **19**(1):88.

21. Shapiro GD, Dodds L, Arbuckle TE, Ashley-Martin J, Ettinger AS, Fisher M, Taback S, Bouchard MF, Monnier P, Dallaire R *et al*: **Exposure to organophosphorus and organochlorine pesticides, perfluoroalkyl substances, and polychlorinated biphenyls in pregnancy and the association with impaired glucose tolerance and gestational diabetes mellitus: The MIREC Study**. *Environ Res* 2016, **147**:71-81.

22. Starling AP, Adgate JL, Hamman RF, Kechris K, Calafat AM, Ye X, Dabelea D: **Perfluoroalkyl Substances during Pregnancy and Offspring Weight and Adiposity at Birth: Examining Mediation by Maternal Fasting Glucose in the Healthy Start Study**. *Environ Health Perspect* 2017, **125**(6):067016.

23. Valvi D, Oulhote Y, Weihe P, Dalgård C, Bjerve KS, Steuerwald U, Grandjean P: **Gestational diabetes and offspring birth size at elevated environmental pollutant exposures**. *Environment international* 2017, **107**:205-215.

24. Wang Y, Starling AP, Haug LS, Eggesbo M, Becher G, Thomsen C, Travlos G, King D, Hoppin JA, Rogan WJ *et al*: **Association between perfluoroalkyl substances and thyroid stimulating hormone among pregnant women: a cross-sectional study**. *Environ Health* 2013, **12**(1):76.

25. Wang Y, Rogan WJ, Chen PC, Lien GW, Chen HY, Tseng YC, Longnecker MP, Wang SL: **Association between maternal serum perfluoroalkyl substances during pregnancy and maternal and cord thyroid hormones: Taiwan maternal and infant cohort study**. *Environ Health Perspect* 2014, **122**(5):529-534.

26. Wang H, Yang J, Du H, Xu L, Liu S, Yi J, Qian X, Chen Y, Jiang Q, He G: **Perfluoroalkyl substances, glucose homeostasis, and gestational diabetes mellitus in Chinese pregnant women: A repeat measurement-based prospective study**. *Environment international* 2018, **114**:12-20.

27. Wang Y, Zhang L, Teng Y, Zhang J, Yang L, Li J, Lai J, Zhao Y, Wu Y: **Association of serum levels of perfluoroalkyl substances with gestational diabetes mellitus and postpartum blood glucose**. *J Environ Sci (China)* 2018, **69**:5-11.

28. Webster GM, Venners SA, Mattman A, Martin JW: **Associations between perfluoroalkyl acids (PFASs) and maternal thyroid hormones in early pregnancy: a population-based cohort study**. *Environ Res* 2014, **133**:338-347.

29. Xiao C, Grandjean P, Valvi D, Nielsen F, Jensen TK, Weihe P, Oulhote Y: **Associations of Exposure to Perfluoroalkyl Substances With Thyroid Hormone Concentrations and Birth Size**. *J Clin Endocrinol Metab* 2020, **105**(3):735-745.

30. Xu H, Zhou Q, Zhang J, Chen X, Zhao H, Lu H, Ma B, Wang Z, Wu C, Ying C *et al*: **Exposure to elevated per- and polyfluoroalkyl substances in early pregnancy is related to increased risk of gestational diabetes mellitus: A nested case-control study in Shanghai, China**. *Environment international* 2020, **143**:105952.

31. Yang L, Li J, Lai J, Luan H, Cai Z, Wang Y, Zhao Y, Wu Y: **Placental Transfer of Perfluoroalkyl Substances and Associations with Thyroid Hormones: Beijing Prenatal Exposure Study**. *Scientific reports* 2016, **6**:21699.

32. Zhang C, Sundaram R, Maisog J, Calafat AM, Barr DB, Buck Louis GM: **A prospective study of prepregnancy serum concentrations of perfluorochemicals and the risk of gestational diabetes**. *Fertil Steril* 2015, **103**(1):184-189.
